# Supplementary material for: Shielding effects of myelin sheath on axolemma depolarization under transverse electric field stimulation
Source: PeerJ. 2018 Dec 3;6:e6020. doi: 10.7717/peerj.6020 (PMC6282940; doi:10.7717/peerj.6020)
Supplement: Supplemental Information 1 [file peerj-06-6020-s001.pdf]

```
/* Mathematica notebook for the transmembrane potential expression for an
  myelinated axon that is under a tranversal DC electric field exposure * /
    /* Step 1 : Solve equations (A5) - (A12) in Appendix * /

Solve[-E0 a + C0 / a == a A1 + C1 / a
  && S0 (-E0 - C0 / a^2) == (A1 - C1 / a^2) S1
  && b A1 + C1 / b == b A2 + C2 / b
  && S1 (A1 - C1 / b^2) == S2 (A2 - C2 / b^2)
  && c A2 + C2 / c == c A3 + C3 / c
  && S2 (A2 - C2 / c^2) == S3 (A3 - C3 / c^2)
  && d A3 + C3 / d == d A4
  && S3 (A3 - C3 / d^2) == S4 A4, {C0, A1, C1, A2, C2, A3, C3, A4}]
{{C0 →
   - ((a^4 b^2 c^2 E0 S0 S1 S2 S3 + a^2 b^4 c^2 E0 S0 S1 S2 S3 + a^4 c^4 E0 S0 S1 S2 S3 + a^2 b^2 c^4 E0 S0 S1 S2
          S3 + a^4 b^2 d^2 E0 S0 S1 S2 S3 + a^2 b^4 d^2 E0 S0 S1 S2 S3 + a^4 c^2 d^2 E0 S0 S1 S2 S3 +
        a^2 b^2 c^2 d^2 E0 S0 S1 S2 S3 - a^4 b^2 c^2 E0 S1^2 S2 S3 + a^2 b^4 c^2 E0 S1^2 S2 S3 -
        a^4 c^4 E0 S1^2 S2 S3 + a^2 b^2 c^4 E0 S1^2 S2 S3 - a^4 b^2 d^2 E0 S1^2 S2 S3 + a^2 b^4 d^2 E0 S1^2 S2 S3 -
        a^4 c^2 d^2 E0 S1^2 S2 S3 + a^2 b^2 c^2 d^2 E0 S1^2 S2 S3 + a^4 b^2 c^2 E0 S0 S2^2 S3 -
        a^2 b^4 c^2 E0 S0 S2^2 S3 - a^4 c^4 E0 S0 S2^2 S3 + a^2 b^2 c^4 E0 S0 S2^2 S3 + a^4 b^2 d^2 E0 S0 S2^2 S3 -
        a^2 b^4 d^2 E0 S0 S2^2 S3 - a^4 c^2 d^2 E0 S0 S2^2 S3 + a^2 b^2 c^2 d^2 E0 S0 S2^2 S3 -
        a^4 b^2 c^2 E0 S1 S2^2 S3 - a^2 b^4 c^2 E0 S1 S2^2 S3 + a^4 c^4 E0 S1 S2^2 S3 + a^2 b^2 c^4 E0 S1 S2^2 S3 -
        a^4 b^2 d^2 E0 S1 S2^2 S3 - a^2 b^4 d^2 E0 S1 S2^2 S3 + a^4 c^2 d^2 E0 S1 S2^2 S3 +
        a^2 b^2 c^2 d^2 E0 S1 S2^2 S3 + a^4 b^2 c^2 E0 S0 S1 S3^2 + a^2 b^4 c^2 E0 S0 S1 S3^2 - a^4 c^4 E0 S0 S1 S3^2 -
        a^2 b^2 c^4 E0 S0 S1 S3^2 - a^4 b^2 d^2 E0 S0 S1 S3^2 - a^2 b^4 d^2 E0 S0 S1 S3^2 + a^4 c^2 d^2 E0 S0 S1 S3^2 +
        a^2 b^2 c^2 d^2 E0 S0 S1 S3^2 - a^4 b^2 c^2 E0 S1^2 S3^2 + a^2 b^4 c^2 E0 S1^2 S3^2 + a^4 c^4 E0 S1^2 S3^2 -
        a^2 b^2 c^4 E0 S1^2 S3^2 + a^4 b^2 d^2 E0 S1^2 S3^2 - a^2 b^4 d^2 E0 S1^2 S3^2 - a^4 c^2 d^2 E0 S1^2 S3^2 +
        a^2 b^2 c^2 d^2 E0 S1^2 S3^2 + a^4 b^2 c^2 E0 S0 S2 S3^2 - a^2 b^4 c^2 E0 S0 S2 S3^2 + a^4 c^4 E0 S0 S2 S3^2 -
        a^2 b^2 c^4 E0 S0 S2 S3^2 - a^4 b^2 d^2 E0 S0 S2 S3^2 + a^2 b^4 d^2 E0 S0 S2 S3^2 - a^4 c^2 d^2 E0 S0 S2 S3^2 +
        a^2 b^2 c^2 d^2 E0 S0 S2 S3^2 - a^4 b^2 c^2 E0 S1 S2 S3^2 - a^2 b^4 c^2 E0 S1 S2 S3^2 - a^4 c^4 E0 S1 S2 S3^2 -
        a^2 b^2 c^4 E0 S1 S2 S3^2 + a^4 b^2 d^2 E0 S1 S2 S3^2 + a^2 b^4 d^2 E0 S1 S2 S3^2 + a^4 c^2 d^2 E0 S1 S2 S3^2 +
        a^2 b^2 c^2 d^2 E0 S1 S2 S3^2 + a^4 b^2 c^2 E0 S0 S1 S2 S4 + a^2 b^4 c^2 E0 S0 S1 S2 S4 +
        a^4 c^4 E0 S0 S1 S2 S4 + a^2 b^2 c^4 E0 S0 S1 S2 S4 - a^4 b^2 d^2 E0 S0 S1 S2 S4 -
        a^2 b^4 d^2 E0 S0 S1 S2 S4 - a^4 c^2 d^2 E0 S0 S1 S2 S4 - a^2 b^2 c^2 d^2 E0 S0 S1 S2 S4 -
        a^4 b^2 c^2 E0 S1^2 S2 S4 + a^2 b^4 c^2 E0 S1^2 S2 S4 - a^4 c^4 E0 S1^2 S2 S4 + a^2 b^2 c^4 E0 S1^2 S2 S4 +
        a^4 b^2 d^2 E0 S1^2 S2 S4 - a^2 b^4 d^2 E0 S1^2 S2 S4 + a^4 c^2 d^2 E0 S1^2 S2 S4 -
        a^2 b^2 c^2 d^2 E0 S1^2 S2 S4 + a^4 b^2 c^2 E0 S0 S2^2 S4 - a^2 b^4 c^2 E0 S0 S2^2 S4 - a^4 c^4 E0 S0 S2^2 S4 +
        a^2 b^2 c^4 E0 S0 S2^2 S4 - a^4 b^2 d^2 E0 S0 S2^2 S4 + a^2 b^4 d^2 E0 S0 S2^2 S4 + a^4 c^2 d^2 E0 S0 S2^2 S4 -
        a^2 b^2 c^2 d^2 E0 S0 S2^2 S4 - a^4 b^2 c^2 E0 S1 S2^2 S4 - a^2 b^4 c^2 E0 S1 S2^2 S4 +
        a^4 c^4 E0 S1 S2^2 S4 + a^2 b^2 c^4 E0 S1 S2^2 S4 + a^4 b^2 d^2 E0 S1 S2^2 S4 + a^2 b^4 d^2 E0 S1 S2^2 S4 -
        a^4 c^2 d^2 E0 S1 S2^2 S4 - a^2 b^2 c^2 d^2 E0 S1 S2^2 S4 + a^4 b^2 c^2 E0 S0 S1 S3 S4 +
        a^2 b^4 c^2 E0 S0 S1 S3 S4 - a^4 c^4 E0 S0 S1 S3 S4 - a^2 b^2 c^4 E0 S0 S1 S3 S4 +
        a^4 b^2 d^2 E0 S0 S1 S3 S4 + a^2 b^4 d^2 E0 S0 S1 S3 S4 - a^4 c^2 d^2 E0 S0 S1 S3 S4 -
        a^2 b^2 c^2 d^2 E0 S0 S1 S3 S4 - a^4 b^2 c^2 E0 S1^2 S3 S4 + a^2 b^4 c^2 E0 S1^2 S3 S4 +
```

$$a^4 c^4 E0\, S1^2 S3\, S4 - a^2 b^2 c^4 E0\, S1^2 S3\, S4 - a^4 b^2 d^2 E0\, S1^2 S3\, S4 + a^2 b^4 d^2 E0\, S1^2 S3\, S4 +$$
$$a^4 c^2 d^2 E0\, S1^2 S3\, S4 - a^2 b^2 c^2 d^2 E0\, S1^2 S3\, S4 + a^4 b^2 c^2 E0\, S0\, S2\, S3\, S4 -$$
$$a^2 b^4 c^2 E0\, S0\, S2\, S3\, S4 + a^4 c^4 E0\, S0\, S2\, S3\, S4 - a^2 b^2 c^4 E0\, S0\, S2\, S3\, S4 +$$
$$a^4 b^2 d^2 E0\, S0\, S2\, S3\, S4 - a^2 b^4 d^2 E0\, S0\, S2\, S3\, S4 + a^4 c^2 d^2 E0\, S0\, S2\, S3\, S4 -$$
$$a^2 b^2 c^2 d^2 E0\, S0\, S2\, S3\, S4 - a^4 b^2 c^2 E0\, S1\, S2\, S3\, S4 - a^2 b^4 c^2 E0\, S1\, S2\, S3\, S4 -$$
$$a^4 c^4 E0\, S1\, S2\, S3\, S4 - a^2 b^2 c^4 E0\, S1\, S2\, S3\, S4 - a^4 b^2 d^2 E0\, S1\, S2\, S3\, S4 -$$
$$a^2 b^4 d^2 E0\, S1\, S2\, S3\, S4 - a^4 c^2 d^2 E0\, S1\, S2\, S3\, S4 - a^2 b^2 c^2 d^2 E0\, S1\, S2\, S3\, S4\Big) \Big/$$
$$\Big(a^2 b^2 c^2 S0\, S1\, S2\, S3 + b^4 c^2 S0\, S1\, S2\, S3 + a^2 c^4 S0\, S1\, S2\, S3 + b^2 c^4 S0\, S1\, S2\, S3 +$$
$$a^2 b^2 d^2 S0\, S1\, S2\, S3 + b^4 d^2 S0\, S1\, S2\, S3 + a^2 c^2 d^2 S0\, S1\, S2\, S3 + b^2 c^2 d^2 S0\, S1\, S2\, S3 +$$
$$a^2 b^2 c^2 S1^2 S2\, S3 - b^4 c^2 S1^2 S2\, S3 + a^2 c^4 S1^2 S2\, S3 - b^2 c^4 S1^2 S2\, S3 + a^2 b^2 d^2 S1^2 S2\, S3 -$$
$$b^4 d^2 S1^2 S2\, S3 + a^2 c^2 d^2 S1^2 S2\, S3 - b^2 c^2 d^2 S1^2 S2\, S3 + a^2 b^2 c^2 S0\, S2^2 S3 -$$
$$b^4 c^2 S0\, S2^2 S3 - a^2 c^4 S0\, S2^2 S3 + b^2 c^4 S0\, S2^2 S3 + a^2 b^2 d^2 S0\, S2^2 S3 - b^4 d^2 S0\, S2^2 S3 -$$
$$a^2 c^2 d^2 S0\, S2^2 S3 + b^2 c^2 d^2 S0\, S2^2 S3 + a^2 b^2 c^2 S1\, S2^2 S3 + b^4 c^2 S1\, S2^2 S3 -$$
$$a^2 c^4 S1\, S2^2 S3 - b^2 c^4 S1\, S2^2 S3 + a^2 b^2 d^2 S1\, S2^2 S3 + b^4 d^2 S1\, S2^2 S3 - a^2 c^2 d^2 S1\, S2^2 S3 -$$
$$b^2 c^2 d^2 S1\, S2^2 S3 + a^2 b^2 c^2 S0\, S1\, S3^2 + b^4 c^2 S0\, S1\, S3^2 - a^2 c^4 S0\, S1\, S3^2 -$$
$$b^2 c^4 S0\, S1\, S3^2 - a^2 b^2 d^2 S0\, S1\, S3^2 - b^4 d^2 S0\, S1\, S3^2 + a^2 c^2 d^2 S0\, S1\, S3^2 +$$
$$b^2 c^2 d^2 S0\, S1\, S3^2 + a^2 b^2 c^2 S1^2 S3^2 - b^4 c^2 S1^2 S3^2 - a^2 c^4 S1^2 S3^2 + b^2 c^4 S1^2 S3^2 -$$
$$a^2 b^2 d^2 S1^2 S3^2 + b^4 d^2 S1^2 S3^2 + a^2 c^2 d^2 S1^2 S3^2 - b^2 c^2 d^2 S1^2 S3^2 + a^2 b^2 c^2 S0\, S2\, S3^2 -$$
$$b^4 c^2 S0\, S2\, S3^2 + a^2 c^4 S0\, S2\, S3^2 - b^2 c^4 S0\, S2\, S3^2 - a^2 b^2 d^2 S0\, S2\, S3^2 + b^4 d^2 S0\, S2\, S3^2 -$$
$$a^2 c^2 d^2 S0\, S2\, S3^2 + b^2 c^2 d^2 S0\, S2\, S3^2 + a^2 b^2 c^2 S1\, S2\, S3^2 + b^4 c^2 S1\, S2\, S3^2 +$$
$$a^2 c^4 S1\, S2\, S3^2 + b^2 c^4 S1\, S2\, S3^2 - a^2 b^2 d^2 S1\, S2\, S3^2 - b^4 d^2 S1\, S2\, S3^2 - a^2 c^2 d^2 S1\, S2\, S3^2 -$$
$$b^2 c^2 d^2 S1\, S2\, S3^2 + a^2 b^2 c^2 S0\, S1\, S2\, S4 + b^4 c^2 S0\, S1\, S2\, S4 + a^2 c^4 S0\, S1\, S2\, S4 +$$
$$b^2 c^4 S0\, S1\, S2\, S4 - a^2 b^2 d^2 S0\, S1\, S2\, S4 - b^4 d^2 S0\, S1\, S2\, S4 - a^2 c^2 d^2 S0\, S1\, S2\, S4 -$$
$$b^2 c^2 d^2 S0\, S1\, S2\, S4 + a^2 b^2 c^2 S1^2 S2\, S4 - b^4 c^2 S1^2 S2\, S4 + a^2 c^4 S1^2 S2\, S4 -$$
$$b^2 c^4 S1^2 S2\, S4 - a^2 b^2 d^2 S1^2 S2\, S4 + b^4 d^2 S1^2 S2\, S4 - a^2 c^2 d^2 S1^2 S2\, S4 +$$
$$b^2 c^2 d^2 S1^2 S2\, S4 + a^2 b^2 c^2 S0\, S2^2 S4 - b^4 c^2 S0\, S2^2 S4 - a^2 c^4 S0\, S2^2 S4 +$$
$$b^2 c^4 S0\, S2^2 S4 - a^2 b^2 d^2 S0\, S2^2 S4 + b^4 d^2 S0\, S2^2 S4 + a^2 c^2 d^2 S0\, S2^2 S4 -$$
$$b^2 c^2 d^2 S0\, S2^2 S4 + a^2 b^2 c^2 S1\, S2^2 S4 + b^4 c^2 S1\, S2^2 S4 - a^2 c^4 S1\, S2^2 S4 - b^2 c^4 S1\, S2^2 S4 -$$
$$a^2 b^2 d^2 S1\, S2^2 S4 - b^4 d^2 S1\, S2^2 S4 + a^2 c^2 d^2 S1\, S2^2 S4 + b^2 c^2 d^2 S1\, S2^2 S4 +$$
$$a^2 b^2 c^2 S0\, S1\, S3\, S4 + b^4 c^2 S0\, S1\, S3\, S4 - a^2 c^4 S0\, S1\, S3\, S4 - b^2 c^4 S0\, S1\, S3\, S4 +$$
$$a^2 b^2 d^2 S0\, S1\, S3\, S4 + b^4 d^2 S0\, S1\, S3\, S4 - a^2 c^2 d^2 S0\, S1\, S3\, S4 - b^2 c^2 d^2 S0\, S1\, S3\, S4 +$$
$$a^2 b^2 c^2 S1^2 S3\, S4 - b^4 c^2 S1^2 S3\, S4 - a^2 c^4 S1^2 S3\, S4 + b^2 c^4 S1^2 S3\, S4 +$$
$$a^2 b^2 d^2 S1^2 S3\, S4 - b^4 d^2 S1^2 S3\, S4 - a^2 c^2 d^2 S1^2 S3\, S4 + b^2 c^2 d^2 S1^2 S3\, S4 +$$
$$a^2 b^2 c^2 S0\, S2\, S3\, S4 - b^4 c^2 S0\, S2\, S3\, S4 + a^2 c^4 S0\, S2\, S3\, S4 - b^2 c^4 S0\, S2\, S3\, S4 +$$
$$a^2 b^2 d^2 S0\, S2\, S3\, S4 - b^4 d^2 S0\, S2\, S3\, S4 + a^2 c^2 d^2 S0\, S2\, S3\, S4 - b^2 c^2 d^2 S0\, S2\, S3\, S4 +$$
$$a^2 b^2 c^2 S1\, S2\, S3\, S4 + b^4 c^2 S1\, S2\, S3\, S4 + a^2 c^4 S1\, S2\, S3\, S4 + b^2 c^4 S1\, S2\, S3\, S4 +$$
$$a^2 b^2 d^2 S1\, S2\, S3\, S4 + b^4 d^2 S1\, S2\, S3\, S4 + a^2 c^2 d^2 S1\, S2\, S3\, S4 + b^2 c^2 d^2 S1\, S2\, S3\, S4\Big)\Big),$$

$$A1 \to -\Bigg(\Bigg(2\, E0\, S0 \Bigg(-\frac{1}{b^3} 2\, S1\, S2 \Bigg(-\frac{2\, S2\, S3 \left(\frac{S3}{d}+\frac{S4}{d}\right)}{c^3} - \left(-\frac{S2}{c^3}+\frac{S3}{c^3}\right)$$
$$\left(S3 \left(\frac{S3}{d}+\frac{S4}{d}\right) + \frac{S3\, (-d\, S3 + d\, S4)}{c^2}\right)\Bigg)\Bigg) - \left(-\frac{S1}{b^3}+\frac{S2}{b^3}\right)$$

$$\left(S2 \left(-\frac{2\ S2\ S3 \left(\frac{S3}{d}+\frac{S4}{d}\right)}{c^3} - \left(-\frac{S2}{c^3}+\frac{S3}{c^3}\right) \left(S3 \left(\frac{S3}{d}+\frac{S4}{d}\right) + \frac{S3\ (-d\ S3 + d\ S4)}{c^2}\right)\right) + \frac{1}{b^2} S2\right.$$

$$\left.\left(-\frac{2\ S3 \left(-\frac{S2\ S3}{d} - \frac{S2\ S4}{d}\right)}{c} - \left(\frac{S2}{c}+\frac{S3}{c}\right) \left(S3 \left(\frac{S3}{d}+\frac{S4}{d}\right) + \frac{S3\ (-d\ S3 + d\ S4)}{c^2}\right)\right)))))\right) \Big/$$

$$\left(a \left(-\left(-\frac{S0}{a} - \frac{S1}{a}\right) \left(-\frac{1}{b^3} 2\ S1\ S2 \left(-\frac{2\ S2\ S3 \left(\frac{S3}{d}+\frac{S4}{d}\right)}{c^3} - \left(-\frac{S2}{c^3}+\frac{S3}{c^3}\right)\right.\right.\right.\right.$$

$$\left.\left(S3 \left(\frac{S3}{d}+\frac{S4}{d}\right) + \frac{S3\ (-d\ S3 + d\ S4)}{c^2}\right)\right) - \left(-\frac{S1}{b^3}+\frac{S2}{b^3}\right)$$

$$\left(S2 \left(-\frac{2\ S2\ S3 \left(\frac{S3}{d}+\frac{S4}{d}\right)}{c^3} - \left(-\frac{S2}{c^3}+\frac{S3}{c^3}\right) \left(S3 \left(\frac{S3}{d}+\frac{S4}{d}\right) + \frac{S3\ (-d\ S3 + d\ S4)}{c^2}\right)\right) + \right.$$

$$\frac{1}{b^2} S2 \left(-\frac{2\ S3 \left(-\frac{S2\ S3}{d} - \frac{S2\ S4}{d}\right)}{c} - \left(\frac{S2}{c}+\frac{S3}{c}\right)\right.$$

$$\left.\left(S3 \left(\frac{S3}{d}+\frac{S4}{d}\right) + \frac{S3\ (-d\ S3 + d\ S4)}{c^2}\right)))\right) + \left(-\frac{S0}{a^3}+\frac{S1}{a^3}\right)$$

$$\left(\frac{1}{b} 2\ S1\ S2 \left(-\frac{2\ S2\ S3 \left(\frac{S3}{d}+\frac{S4}{d}\right)}{c^3} - \left(-\frac{S2}{c^3}+\frac{S3}{c^3}\right) \left(S3 \left(\frac{S3}{d}+\frac{S4}{d}\right) + \frac{S3\ (-d\ S3 + d\ S4)}{c^2}\right)\right) - \right.$$

$$\left(\frac{S1}{b}+\frac{S2}{b}\right) \left(S2 \left(-\frac{2\ S2\ S3 \left(\frac{S3}{d}+\frac{S4}{d}\right)}{c^3} - \left(-\frac{S2}{c^3}+\frac{S3}{c^3}\right)\right.\right.$$

$$\left.\left(S3 \left(\frac{S3}{d}+\frac{S4}{d}\right) + \frac{S3\ (-d\ S3 + d\ S4)}{c^2}\right)\right) + \frac{1}{b^2} S2 \left(-\frac{2\ S3 \left(-\frac{S2\ S3}{d} - \frac{S2\ S4}{d}\right)}{c} - \right.$$

$$\left.\left(\frac{S2}{c}+\frac{S3}{c}\right) \left(S3 \left(\frac{S3}{d}+\frac{S4}{d}\right) + \frac{S3\ (-d\ S3 + d\ S4)}{c^2}\right)))))))\right),$$

$$C1 \to -\left(\left(2\ E0\ S0\ \left(a^2 b^4 c^2\ S1\ S2\ S3 + a^2 b^2 c^4\ S1\ S2\ S3 + a^2 b^4 d^2\ S1\ S2\ S3 + a^2 b^2 c^2 d^2\ S1\ S2\ S3 -\right.\right.\right.$$
$$a^2 b^4 c^2\ S2^2\ S3 + a^2 b^2 c^4\ S2^2\ S3 - a^2 b^4 d^2\ S2^2\ S3 + a^2 b^2 c^2 d^2\ S2^2\ S3 +$$
$$a^2 b^4 c^2\ S1\ S3^2 - a^2 b^2 c^4\ S1\ S3^2 - a^2 b^4 d^2\ S1\ S3^2 + a^2 b^2 c^2 d^2\ S1\ S3^2 -$$
$$a^2 b^4 c^2\ S2\ S3^2 - a^2 b^2 c^4\ S2\ S3^2 + a^2 b^4 d^2\ S2\ S3^2 + a^2 b^2 c^2 d^2\ S2\ S3^2 +$$
$$a^2 b^4 c^2\ S1\ S2\ S4 + a^2 b^2 c^4\ S1\ S2\ S4 - a^2 b^4 d^2\ S1\ S2\ S4 - a^2 b^2 c^2 d^2\ S1\ S2\ S4 -$$
$$a^2 b^4 c^2\ S2^2\ S4 + a^2 b^2 c^4\ S2^2\ S4 + a^2 b^4 d^2\ S2^2\ S4 - a^2 b^2 c^2 d^2\ S2^2\ S4 +$$
$$a^2 b^4 c^2\ S1\ S3\ S4 - a^2 b^2 c^4\ S1\ S3\ S4 + a^2 b^4 d^2\ S1\ S3\ S4 - a^2 b^2 c^2 d^2\ S1\ S3\ S4 -$$
$$\left.\left. a^2 b^4 c^2\ S2\ S3\ S4 - a^2 b^2 c^4\ S2\ S3\ S4 - a^2 b^4 d^2\ S2\ S3\ S4 - a^2 b^2 c^2 d^2\ S2\ S3\ S4\right)\right) \Big/$$
$$\left(a^2 b^2 c^2\ S0\ S1\ S2\ S3 + b^4 c^2\ S0\ S1\ S2\ S3 + a^2 c^4\ S0\ S1\ S2\ S3 + b^2 c^4\ S0\ S1\ S2\ S3 +\right.$$
$$a^2 b^2 d^2\ S0\ S1\ S2\ S3 + b^4 d^2\ S0\ S1\ S2\ S3 + a^2 c^2 d^2\ S0\ S1\ S2\ S3 + b^2 c^2 d^2\ S0\ S1\ S2\ S3 +$$
$$a^2 b^2 c^2\ S1^2\ S2\ S3 - b^4 c^2\ S1^2\ S2\ S3 + a^2 c^4\ S1^2\ S2\ S3 - b^2 c^4\ S1^2\ S2\ S3 +$$

$a^2 b^2 d^2 S1^2 S2 S3 - b^4 d^2 S1^2 S2 S3 + a^2 c^2 d^2 S1^2 S2 S3 - b^2 c^2 d^2 S1^2 S2 S3 +$
$a^2 b^2 c^2 S0 S2^2 S3 - b^4 c^2 S0 S2^2 S3 - a^2 c^4 S0 S2^2 S3 + b^2 c^4 S0 S2^2 S3 +$
$a^2 b^2 d^2 S0 S2^2 S3 - b^4 d^2 S0 S2^2 S3 - a^2 c^2 d^2 S0 S2^2 S3 + b^2 c^2 d^2 S0 S2^2 S3 +$
$a^2 b^2 c^2 S1 S2^2 S3 + b^4 c^2 S1 S2^2 S3 - a^2 c^4 S1 S2^2 S3 - b^2 c^4 S1 S2^2 S3 +$
$a^2 b^2 d^2 S1 S2^2 S3 + b^4 d^2 S1 S2^2 S3 - a^2 c^2 d^2 S1 S2^2 S3 - b^2 c^2 d^2 S1 S2^2 S3 +$
$a^2 b^2 c^2 S0 S1 S3^2 + b^4 c^2 S0 S1 S3^2 - a^2 c^4 S0 S1 S3^2 - b^2 c^4 S0 S1 S3^2 -$
$a^2 b^2 d^2 S0 S1 S3^2 - b^4 d^2 S0 S1 S3^2 + a^2 c^2 d^2 S0 S1 S3^2 + b^2 c^2 d^2 S0 S1 S3^2 +$
$a^2 b^2 c^2 S1^2 S3^2 - b^4 c^2 S1^2 S3^2 - a^2 c^4 S1^2 S3^2 + b^2 c^4 S1^2 S3^2 - a^2 b^2 d^2 S1^2 S3^2 +$
$b^4 d^2 S1^2 S3^2 + a^2 c^2 d^2 S1^2 S3^2 - b^2 c^2 d^2 S1^2 S3^2 + a^2 b^2 c^2 S0 S2 S3^2 -$
$b^4 c^2 S0 S2 S3^2 + a^2 c^4 S0 S2 S3^2 - b^2 c^4 S0 S2 S3^2 - a^2 b^2 d^2 S0 S2 S3^2 +$
$b^4 d^2 S0 S2 S3^2 - a^2 c^2 d^2 S0 S2 S3^2 + b^2 c^2 d^2 S0 S2 S3^2 + a^2 b^2 c^2 S1 S2 S3^2 +$
$b^4 c^2 S1 S2 S3^2 + a^2 c^4 S1 S2 S3^2 + b^2 c^4 S1 S2 S3^2 - a^2 b^2 d^2 S1 S2 S3^2 -$
$b^4 d^2 S1 S2 S3^2 - a^2 c^2 d^2 S1 S2 S3^2 - b^2 c^2 d^2 S1 S2 S3^2 + a^2 b^2 c^2 S0 S1 S2 S4 +$
$b^4 c^2 S0 S1 S2 S4 + a^2 c^4 S0 S1 S2 S4 + b^2 c^4 S0 S1 S2 S4 - a^2 b^2 d^2 S0 S1 S2 S4 -$
$b^4 d^2 S0 S1 S2 S4 - a^2 c^2 d^2 S0 S1 S2 S4 - b^2 c^2 d^2 S0 S1 S2 S4 + a^2 b^2 c^2 S1^2 S2 S4 -$
$b^4 c^2 S1^2 S2 S4 + a^2 c^4 S1^2 S2 S4 - b^2 c^4 S1^2 S2 S4 - a^2 b^2 d^2 S1^2 S2 S4 +$
$b^4 d^2 S1^2 S2 S4 - a^2 c^2 d^2 S1^2 S2 S4 + b^2 c^2 d^2 S1^2 S2 S4 + a^2 b^2 c^2 S0 S2^2 S4 -$
$b^4 c^2 S0 S2^2 S4 - a^2 c^4 S0 S2^2 S4 + b^2 c^4 S0 S2^2 S4 - a^2 b^2 d^2 S0 S2^2 S4 +$
$b^4 d^2 S0 S2^2 S4 + a^2 c^2 d^2 S0 S2^2 S4 - b^2 c^2 d^2 S0 S2^2 S4 + a^2 b^2 c^2 S1 S2^2 S4 +$
$b^4 c^2 S1 S2^2 S4 - a^2 c^4 S1 S2^2 S4 - b^2 c^4 S1 S2^2 S4 - a^2 b^2 d^2 S1 S2^2 S4 -$
$b^4 d^2 S1 S2^2 S4 + a^2 c^2 d^2 S1 S2^2 S4 + b^2 c^2 d^2 S1 S2^2 S4 + a^2 b^2 c^2 S0 S1 S3 S4 +$
$b^4 c^2 S0 S1 S3 S4 - a^2 c^4 S0 S1 S3 S4 - b^2 c^4 S0 S1 S3 S4 + a^2 b^2 d^2 S0 S1 S3 S4 +$
$b^4 d^2 S0 S1 S3 S4 - a^2 c^2 d^2 S0 S1 S3 S4 - b^2 c^2 d^2 S0 S1 S3 S4 + a^2 b^2 c^2 S1^2 S3 S4 -$
$b^4 c^2 S1^2 S3 S4 - a^2 c^4 S1^2 S3 S4 + b^2 c^4 S1^2 S3 S4 + a^2 b^2 d^2 S1^2 S3 S4 -$
$b^4 d^2 S1^2 S3 S4 - a^2 c^2 d^2 S1^2 S3 S4 + b^2 c^2 d^2 S1^2 S3 S4 + a^2 b^2 c^2 S0 S2 S3 S4 -$
$b^4 c^2 S0 S2 S3 S4 + a^2 c^4 S0 S2 S3 S4 - b^2 c^4 S0 S2 S3 S4 + a^2 b^2 d^2 S0 S2 S3 S4 -$
$b^4 d^2 S0 S2 S3 S4 + a^2 c^2 d^2 S0 S2 S3 S4 - b^2 c^2 d^2 S0 S2 S3 S4 + a^2 b^2 c^2 S1 S2 S3 S4 +$
$b^4 c^2 S1 S2 S3 S4 + a^2 c^4 S1 S2 S3 S4 + b^2 c^4 S1 S2 S3 S4 + a^2 b^2 d^2 S1 S2 S3 S4 +$
$b^4 d^2 S1 S2 S3 S4 + a^2 c^2 d^2 S1 S2 S3 S4 + b^2 c^2 d^2 S1 S2 S3 S4)),$

$A2 \rightarrow -((4 b^2 E0 S0 (a^2 c^2 S1 S2 S3 + a^2 d^2 S1 S2 S3 + a^2 c^2 S1 S3^2 - a^2 d^2 S1 S3^2 +$
$a^2 c^2 S1 S2 S4 - a^2 d^2 S1 S2 S4 + a^2 c^2 S1 S3 S4 + a^2 d^2 S1 S3 S4)) /$
$(a^2 b^2 c^2 S0 S1 S2 S3 + b^4 c^2 S0 S1 S2 S3 + a^2 c^4 S0 S1 S2 S3 + b^2 c^4 S0 S1 S2 S3 +$
$a^2 b^2 d^2 S0 S1 S2 S3 + b^4 d^2 S0 S1 S2 S3 + a^2 c^2 d^2 S0 S1 S2 S3 + b^2 c^2 d^2 S0 S1 S2 S3 +$
$a^2 b^2 c^2 S1^2 S2 S3 - b^4 c^2 S1^2 S2 S3 + a^2 c^4 S1^2 S2 S3 - b^2 c^4 S1^2 S2 S3 +$
$a^2 b^2 d^2 S1^2 S2 S3 - b^4 d^2 S1^2 S2 S3 + a^2 c^2 d^2 S1^2 S2 S3 - b^2 c^2 d^2 S1^2 S2 S3 +$
$a^2 b^2 c^2 S0 S2^2 S3 - b^4 c^2 S0 S2^2 S3 - a^2 c^4 S0 S2^2 S3 + b^2 c^4 S0 S2^2 S3 +$
$a^2 b^2 d^2 S0 S2^2 S3 - b^4 d^2 S0 S2^2 S3 - a^2 c^2 d^2 S0 S2^2 S3 + b^2 c^2 d^2 S0 S2^2 S3 +$
$a^2 b^2 c^2 S1 S2^2 S3 + b^4 c^2 S1 S2^2 S3 - a^2 c^4 S1 S2^2 S3 - b^2 c^4 S1 S2^2 S3 +$
$a^2 b^2 d^2 S1 S2^2 S3 + b^4 d^2 S1 S2^2 S3 - a^2 c^2 d^2 S1 S2^2 S3 - b^2 c^2 d^2 S1 S2^2 S3 +$
$a^2 b^2 c^2 S0 S1 S3^2 + b^4 c^2 S0 S1 S3^2 - a^2 c^4 S0 S1 S3^2 - b^2 c^4 S0 S1 S3^2 -$
$a^2 b^2 d^2 S0 S1 S3^2 - b^4 d^2 S0 S1 S3^2 + a^2 c^2 d^2 S0 S1 S3^2 + b^2 c^2 d^2 S0 S1 S3^2 +$
$a^2 b^2 c^2 S1^2 S3^2 - b^4 c^2 S1^2 S3^2 - a^2 c^4 S1^2 S3^2 + b^2 c^4 S1^2 S3^2 - a^2 b^2 d^2 S1^2 S3^2 +$
$b^4 d^2 S1^2 S3^2 + a^2 c^2 d^2 S1^2 S3^2 - b^2 c^2 d^2 S1^2 S3^2 + a^2 b^2 c^2 S0 S2 S3^2 -$

$b^4 c^2 S0\,S2\,S3^2 + a^2 c^4 S0\,S2\,S3^2 - b^2 c^4 S0\,S2\,S3^2 - a^2 b^2 d^2 S0\,S2\,S3^2 +$
$b^4 d^2 S0\,S2\,S3^2 - a^2 c^2 d^2 S0\,S2\,S3^2 + b^2 c^2 d^2 S0\,S2\,S3^2 + a^2 b^2 c^2 S1\,S2\,S3^2 +$
$b^4 c^2 S1\,S2\,S3^2 + a^2 c^4 S1\,S2\,S3^2 + b^2 c^4 S1\,S2\,S3^2 - a^2 b^2 d^2 S1\,S2\,S3^2 -$
$b^4 d^2 S1\,S2\,S3^2 - a^2 c^2 d^2 S1\,S2\,S3^2 - b^2 c^2 d^2 S1\,S2\,S3^2 + a^2 b^2 c^2 S0\,S1\,S2\,S4 +$
$b^4 c^2 S0\,S1\,S2\,S4 + a^2 c^4 S0\,S1\,S2\,S4 + b^2 c^4 S0\,S1\,S2\,S4 - a^2 b^2 d^2 S0\,S1\,S2\,S4 -$
$b^4 d^2 S0\,S1\,S2\,S4 - a^2 c^2 d^2 S0\,S1\,S2\,S4 - b^2 c^2 d^2 S0\,S1\,S2\,S4 + a^2 b^2 c^2 S1^2 S2\,S4 -$
$b^4 c^2 S1^2 S2\,S4 + a^2 c^4 S1^2 S2\,S4 - b^2 c^4 S1^2 S2\,S4 - a^2 b^2 d^2 S1^2 S2\,S4 +$
$b^4 d^2 S1^2 S2\,S4 - a^2 c^2 d^2 S1^2 S2\,S4 + b^2 c^2 d^2 S1^2 S2\,S4 + a^2 b^2 c^2 S0\,S2^2 S4 -$
$b^4 c^2 S0\,S2^2 S4 - a^2 c^4 S0\,S2^2 S4 + b^2 c^4 S0\,S2^2 S4 - a^2 b^2 d^2 S0\,S2^2 S4 +$
$b^4 d^2 S0\,S2^2 S4 + a^2 c^2 d^2 S0\,S2^2 S4 - b^2 c^2 d^2 S0\,S2^2 S4 + a^2 b^2 c^2 S1\,S2^2 S4 +$
$b^4 c^2 S1\,S2^2 S4 - a^2 c^4 S1\,S2^2 S4 - b^2 c^4 S1\,S2^2 S4 - a^2 b^2 d^2 S1\,S2^2 S4 -$
$b^4 d^2 S1\,S2^2 S4 + a^2 c^2 d^2 S1\,S2^2 S4 + b^2 c^2 d^2 S1\,S2^2 S4 + a^2 b^2 c^2 S0\,S1\,S3\,S4 +$
$b^4 c^2 S0\,S1\,S3\,S4 - a^2 c^4 S0\,S1\,S3\,S4 - b^2 c^4 S0\,S1\,S3\,S4 + a^2 b^2 d^2 S0\,S1\,S3\,S4 +$
$b^4 d^2 S0\,S1\,S3\,S4 - a^2 c^2 d^2 S0\,S1\,S3\,S4 - b^2 c^2 d^2 S0\,S1\,S3\,S4 + a^2 b^2 c^2 S1^2 S3\,S4 -$
$b^4 c^2 S1^2 S3\,S4 - a^2 c^4 S1^2 S3\,S4 + b^2 c^4 S1^2 S3\,S4 + a^2 b^2 d^2 S1^2 S3\,S4 -$
$b^4 d^2 S1^2 S3\,S4 - a^2 c^2 d^2 S1^2 S3\,S4 + b^2 c^2 d^2 S1^2 S3\,S4 + a^2 b^2 c^2 S0\,S2\,S3\,S4 -$
$b^4 c^2 S0\,S2\,S3\,S4 + a^2 c^4 S0\,S2\,S3\,S4 - b^2 c^4 S0\,S2\,S3\,S4 + a^2 b^2 d^2 S0\,S2\,S3\,S4 -$
$b^4 d^2 S0\,S2\,S3\,S4 + a^2 c^2 d^2 S0\,S2\,S3\,S4 - b^2 c^2 d^2 S0\,S2\,S3\,S4 + a^2 b^2 c^2 S1\,S2\,S3\,S4 +$
$b^4 c^2 S1\,S2\,S3\,S4 + a^2 c^4 S1\,S2\,S3\,S4 + b^2 c^4 S1\,S2\,S3\,S4 + a^2 b^2 d^2 S1\,S2\,S3\,S4 +$
$b^4 d^2 S1\,S2\,S3\,S4 + a^2 c^2 d^2 S1\,S2\,S3\,S4 + b^2 c^2 d^2 S1\,S2\,S3\,S4)),$

$C2 \to -((4\,E0\,S0\,(a^2 b^2 c^4 S1\,S2\,S3 + a^2 b^2 c^2 d^2 S1\,S2\,S3 - a^2 b^2 c^4 S1\,S3^2 + a^2 b^2 c^2 d^2 S1\,S3^2 +$
$a^2 b^2 c^4 S1\,S2\,S4 - a^2 b^2 c^2 d^2 S1\,S2\,S4 - a^2 b^2 c^4 S1\,S3\,S4 - a^2 b^2 c^2 d^2 S1\,S3\,S4)) /$
$(a^2 b^2 c^2 S0\,S1\,S2\,S3 + b^4 c^2 S0\,S1\,S2\,S3 + a^2 c^4 S0\,S1\,S2\,S3 + b^2 c^4 S0\,S1\,S2\,S3 +$
$a^2 b^2 d^2 S0\,S1\,S2\,S3 + b^4 d^2 S0\,S1\,S2\,S3 + a^2 c^2 d^2 S0\,S1\,S2\,S3 + b^2 c^2 d^2 S0\,S1\,S2\,S3 +$
$a^2 b^2 c^2 S1^2 S2\,S3 - b^4 c^2 S1^2 S2\,S3 + a^2 c^4 S1^2 S2\,S3 - b^2 c^4 S1^2 S2\,S3 +$
$a^2 b^2 d^2 S1^2 S2\,S3 - b^4 d^2 S1^2 S2\,S3 + a^2 c^2 d^2 S1^2 S2\,S3 - b^2 c^2 d^2 S1^2 S2\,S3 +$
$a^2 b^2 c^2 S0\,S2^2 S3 - b^4 c^2 S0\,S2^2 S3 - a^2 c^4 S0\,S2^2 S3 + b^2 c^4 S0\,S2^2 S3 +$
$a^2 b^2 d^2 S0\,S2^2 S3 - b^4 d^2 S0\,S2^2 S3 - a^2 c^2 d^2 S0\,S2^2 S3 + b^2 c^2 d^2 S0\,S2^2 S3 +$
$a^2 b^2 c^2 S1\,S2^2 S3 + b^4 c^2 S1\,S2^2 S3 - a^2 c^4 S1\,S2^2 S3 - b^2 c^4 S1\,S2^2 S3 +$
$a^2 b^2 d^2 S1\,S2^2 S3 + b^4 d^2 S1\,S2^2 S3 - a^2 c^2 d^2 S1\,S2^2 S3 - b^2 c^2 d^2 S1\,S2^2 S3 +$
$a^2 b^2 c^2 S0\,S1\,S3^2 + b^4 c^2 S0\,S1\,S3^2 - a^2 c^4 S0\,S1\,S3^2 - b^2 c^4 S0\,S1\,S3^2 -$
$a^2 b^2 d^2 S0\,S1\,S3^2 - b^4 d^2 S0\,S1\,S3^2 + a^2 c^2 d^2 S0\,S1\,S3^2 + b^2 c^2 d^2 S0\,S1\,S3^2 +$
$a^2 b^2 c^2 S1^2 S3^2 - b^4 c^2 S1^2 S3^2 - a^2 c^4 S1^2 S3^2 + b^2 c^4 S1^2 S3^2 - a^2 b^2 d^2 S1^2 S3^2 +$
$b^4 d^2 S1^2 S3^2 + a^2 c^2 d^2 S1^2 S3^2 - b^2 c^2 d^2 S1^2 S3^2 + a^2 b^2 c^2 S0\,S2\,S3^2 -$
$b^4 c^2 S0\,S2\,S3^2 + a^2 c^4 S0\,S2\,S3^2 - b^2 c^4 S0\,S2\,S3^2 - a^2 b^2 d^2 S0\,S2\,S3^2 +$
$b^4 d^2 S0\,S2\,S3^2 - a^2 c^2 d^2 S0\,S2\,S3^2 + b^2 c^2 d^2 S0\,S2\,S3^2 + a^2 b^2 c^2 S1\,S2\,S3^2 +$
$b^4 c^2 S1\,S2\,S3^2 + a^2 c^4 S1\,S2\,S3^2 + b^2 c^4 S1\,S2\,S3^2 - a^2 b^2 d^2 S1\,S2\,S3^2 -$
$b^4 d^2 S1\,S2\,S3^2 - a^2 c^2 d^2 S1\,S2\,S3^2 - b^2 c^2 d^2 S1\,S2\,S3^2 + a^2 b^2 c^2 S0\,S1\,S2\,S4 +$
$b^4 c^2 S0\,S1\,S2\,S4 + a^2 c^4 S0\,S1\,S2\,S4 + b^2 c^4 S0\,S1\,S2\,S4 - a^2 b^2 d^2 S0\,S1\,S2\,S4 -$
$b^4 d^2 S0\,S1\,S2\,S4 - a^2 c^2 d^2 S0\,S1\,S2\,S4 - b^2 c^2 d^2 S0\,S1\,S2\,S4 + a^2 b^2 c^2 S1^2 S2\,S4 -$
$b^4 c^2 S1^2 S2\,S4 + a^2 c^4 S1^2 S2\,S4 - b^2 c^4 S1^2 S2\,S4 - a^2 b^2 d^2 S1^2 S2\,S4 +$
$b^4 d^2 S1^2 S2\,S4 - a^2 c^2 d^2 S1^2 S2\,S4 + b^2 c^2 d^2 S1^2 S2\,S4 + a^2 b^2 c^2 S0\,S2^2 S4 -$
$b^4 c^2 S0\,S2^2 S4 - a^2 c^4 S0\,S2^2 S4 + b^2 c^4 S0\,S2^2 S4 - a^2 b^2 d^2 S0\,S2^2 S4 +$

$b^4 d^2 S0 S2^2 S4 + a^2 c^2 d^2 S0 S2^2 S4 - b^2 c^2 d^2 S0 S2^2 S4 + a^2 b^2 c^2 S1 S2^2 S4 +$
$b^4 c^2 S1 S2^2 S4 - a^2 c^4 S1 S2^2 S4 - b^2 c^4 S1 S2^2 S4 - a^2 b^2 d^2 S1 S2^2 S4 -$
$b^4 d^2 S1 S2^2 S4 + a^2 c^2 d^2 S1 S2^2 S4 + b^2 c^2 d^2 S1 S2^2 S4 + a^2 b^2 c^2 S0 S1 S3 S4 +$
$b^4 c^2 S0 S1 S3 S4 - a^2 c^4 S0 S1 S3 S4 - b^2 c^4 S0 S1 S3 S4 + a^2 b^2 d^2 S0 S1 S3 S4 +$
$b^4 d^2 S0 S1 S3 S4 - a^2 c^2 d^2 S0 S1 S3 S4 - b^2 c^2 d^2 S0 S1 S3 S4 + a^2 b^2 c^2 S1^2 S3 S4 -$
$b^4 c^2 S1^2 S3 S4 - a^2 c^4 S1^2 S3 S4 + b^2 c^4 S1^2 S3 S4 + a^2 b^2 d^2 S1^2 S3 S4 -$
$b^4 d^2 S1^2 S3 S4 - a^2 c^2 d^2 S1^2 S3 S4 + b^2 c^2 d^2 S1^2 S3 S4 + a^2 b^2 c^2 S0 S2 S3 S4 -$
$b^4 c^2 S0 S2 S3 S4 + a^2 c^4 S0 S2 S3 S4 - b^2 c^4 S0 S2 S3 S4 + a^2 b^2 d^2 S0 S2 S3 S4 -$
$b^4 d^2 S0 S2 S3 S4 + a^2 c^2 d^2 S0 S2 S3 S4 - b^2 c^2 d^2 S0 S2 S3 S4 + a^2 b^2 c^2 S1 S2 S3 S4 +$
$b^4 c^2 S1 S2 S3 S4 + a^2 c^4 S1 S2 S3 S4 + b^2 c^4 S1 S2 S3 S4 + a^2 b^2 d^2 S1 S2 S3 S4 +$
$b^4 d^2 S1 S2 S3 S4 + a^2 c^2 d^2 S1 S2 S3 S4 + b^2 c^2 d^2 S1 S2 S3 S4)),$

$A3 \to -((8 b^2 c^2 E0 S0 S2 (a^2 S1 S3 + a^2 S1 S4)) / (a^2 b^2 c^2 S0 S1 S2 S3 + b^4 c^2 S0 S1 S2 S3 +$
$a^2 c^4 S0 S1 S2 S3 + b^2 c^4 S0 S1 S2 S3 + a^2 b^2 d^2 S0 S1 S2 S3 + b^4 d^2 S0 S1 S2 S3 +$
$a^2 c^2 d^2 S0 S1 S2 S3 + b^2 c^2 d^2 S0 S1 S2 S3 + a^2 b^2 c^2 S1^2 S2 S3 - b^4 c^2 S1^2 S2 S3 +$
$a^2 c^4 S1^2 S2 S3 - b^2 c^4 S1^2 S2 S3 + a^2 b^2 d^2 S1^2 S2 S3 - b^4 d^2 S1^2 S2 S3 +$
$a^2 c^2 d^2 S1^2 S2 S3 - b^2 c^2 d^2 S1^2 S2 S3 + a^2 b^2 c^2 S0 S2^2 S3 - b^4 c^2 S0 S2^2 S3 -$
$a^2 c^4 S0 S2^2 S3 + b^2 c^4 S0 S2^2 S3 + a^2 b^2 d^2 S0 S2^2 S3 - b^4 d^2 S0 S2^2 S3 -$
$a^2 c^2 d^2 S0 S2^2 S3 + b^2 c^2 d^2 S0 S2^2 S3 + a^2 b^2 c^2 S1 S2^2 S3 + b^4 c^2 S1 S2^2 S3 -$
$a^2 c^4 S1 S2^2 S3 - b^2 c^4 S1 S2^2 S3 + a^2 b^2 d^2 S1 S2^2 S3 + b^4 d^2 S1 S2^2 S3 -$
$a^2 c^2 d^2 S1 S2^2 S3 - b^2 c^2 d^2 S1 S2^2 S3 + a^2 b^2 c^2 S0 S1 S3^2 + b^4 c^2 S0 S1 S3^2 -$
$a^2 c^4 S0 S1 S3^2 - b^2 c^4 S0 S1 S3^2 - a^2 b^2 d^2 S0 S1 S3^2 - b^4 d^2 S0 S1 S3^2 +$
$a^2 c^2 d^2 S0 S1 S3^2 + b^2 c^2 d^2 S0 S1 S3^2 + a^2 b^2 c^2 S1^2 S3^2 - b^4 c^2 S1^2 S3^2 -$
$a^2 c^4 S1^2 S3^2 + b^2 c^4 S1^2 S3^2 - a^2 b^2 d^2 S1^2 S3^2 + b^4 d^2 S1^2 S3^2 + a^2 c^2 d^2 S1^2 S3^2 -$
$b^2 c^2 d^2 S1^2 S3^2 + a^2 b^2 c^2 S0 S2 S3^2 - b^4 c^2 S0 S2 S3^2 + a^2 c^4 S0 S2 S3^2 -$
$b^2 c^4 S0 S2 S3^2 - a^2 b^2 d^2 S0 S2 S3^2 + b^4 d^2 S0 S2 S3^2 - a^2 c^2 d^2 S0 S2 S3^2 +$
$b^2 c^2 d^2 S0 S2 S3^2 + a^2 b^2 c^2 S1 S2 S3^2 + b^4 c^2 S1 S2 S3^2 + a^2 c^4 S1 S2 S3^2 +$
$b^2 c^4 S1 S2 S3^2 - a^2 b^2 d^2 S1 S2 S3^2 - b^4 d^2 S1 S2 S3^2 - a^2 c^2 d^2 S1 S2 S3^2 -$
$b^2 c^2 d^2 S1 S2 S3^2 + a^2 b^2 c^2 S0 S1 S2 S4 + b^4 c^2 S0 S1 S2 S4 + a^2 c^4 S0 S1 S2 S4 +$
$b^2 c^4 S0 S1 S2 S4 - a^2 b^2 d^2 S0 S1 S2 S4 - b^4 d^2 S0 S1 S2 S4 - a^2 c^2 d^2 S0 S1 S2 S4 -$
$b^2 c^2 d^2 S0 S1 S2 S4 + a^2 b^2 c^2 S1^2 S2 S4 - b^4 c^2 S1^2 S2 S4 + a^2 c^4 S1^2 S2 S4 -$
$b^2 c^4 S1^2 S2 S4 - a^2 b^2 d^2 S1^2 S2 S4 + b^4 d^2 S1^2 S2 S4 - a^2 c^2 d^2 S1^2 S2 S4 +$
$b^2 c^2 d^2 S1^2 S2 S4 + a^2 b^2 c^2 S0 S2^2 S4 - b^4 c^2 S0 S2^2 S4 - a^2 c^4 S0 S2^2 S4 +$
$b^2 c^4 S0 S2^2 S4 - a^2 b^2 d^2 S0 S2^2 S4 + b^4 d^2 S0 S2^2 S4 + a^2 c^2 d^2 S0 S2^2 S4 -$
$b^2 c^2 d^2 S0 S2^2 S4 + a^2 b^2 c^2 S1 S2^2 S4 + b^4 c^2 S1 S2^2 S4 - a^2 c^4 S1 S2^2 S4 - b^2 c^4 S1 S2^2 S4 -$
$a^2 b^2 d^2 S1 S2^2 S4 - b^4 d^2 S1 S2^2 S4 + a^2 c^2 d^2 S1 S2^2 S4 + b^2 c^2 d^2 S1 S2^2 S4 +$
$a^2 b^2 c^2 S0 S1 S3 S4 + b^4 c^2 S0 S1 S3 S4 - a^2 c^4 S0 S1 S3 S4 - b^2 c^4 S0 S1 S3 S4 +$
$a^2 b^2 d^2 S0 S1 S3 S4 + b^4 d^2 S0 S1 S3 S4 - a^2 c^2 d^2 S0 S1 S3 S4 - b^2 c^2 d^2 S0 S1 S3 S4 +$
$a^2 b^2 c^2 S1^2 S3 S4 - b^4 c^2 S1^2 S3 S4 - a^2 c^4 S1^2 S3 S4 + b^2 c^4 S1^2 S3 S4 +$
$a^2 b^2 d^2 S1^2 S3 S4 - b^4 d^2 S1^2 S3 S4 - a^2 c^2 d^2 S1^2 S3 S4 + b^2 c^2 d^2 S1^2 S3 S4 +$
$a^2 b^2 c^2 S0 S2 S3 S4 - b^4 c^2 S0 S2 S3 S4 + a^2 c^4 S0 S2 S3 S4 - b^2 c^4 S0 S2 S3 S4 +$
$a^2 b^2 d^2 S0 S2 S3 S4 - b^4 d^2 S0 S2 S3 S4 + a^2 c^2 d^2 S0 S2 S3 S4 - b^2 c^2 d^2 S0 S2 S3 S4 +$
$a^2 b^2 c^2 S1 S2 S3 S4 + b^4 c^2 S1 S2 S3 S4 + a^2 c^4 S1 S2 S3 S4 + b^2 c^4 S1 S2 S3 S4 +$
$a^2 b^2 d^2 S1 S2 S3 S4 + b^4 d^2 S1 S2 S3 S4 + a^2 c^2 d^2 S1 S2 S3 S4 + b^2 c^2 d^2 S1 S2 S3 S4)),$

$$
\begin{array}{l}
C3 \rightarrow -((8 b^2 E0 S0 S2 (a^2 c^2 d^2 S1 S3 - a^2 c^2 d^2 S1 S4)) / \\
(a^2 b^2 c^2 S0 S1 S2 S3 + b^4 c^2 S0 S1 S2 S3 + a^2 c^4 S0 S1 S2 S3 + b^2 c^4 S0 S1 S2 S3 + \\
a^2 b^2 d^2 S0 S1 S2 S3 + b^4 d^2 S0 S1 S2 S3 + a^2 c^2 d^2 S0 S1 S2 S3 + b^2 c^2 d^2 S0 S1 S2 S3 + \\
a^2 b^2 c^2 S1^2 S2 S3 - b^4 c^2 S1^2 S2 S3 + a^2 c^4 S1^2 S2 S3 - b^2 c^4 S1^2 S2 S3 + \\
a^2 b^2 d^2 S1^2 S2 S3 - b^4 d^2 S1^2 S2 S3 + a^2 c^2 d^2 S1^2 S2 S3 - b^2 c^2 d^2 S1^2 S2 S3 + \\
a^2 b^2 c^2 S0 S2^2 S3 - b^4 c^2 S0 S2^2 S3 - a^2 c^4 S0 S2^2 S3 + b^2 c^4 S0 S2^2 S3 + \\
a^2 b^2 d^2 S0 S2^2 S3 - b^4 d^2 S0 S2^2 S3 - a^2 c^2 d^2 S0 S2^2 S3 + b^2 c^2 d^2 S0 S2^2 S3 + \\
a^2 b^2 c^2 S1 S2^2 S3 + b^4 c^2 S1 S2^2 S3 - a^2 c^4 S1 S2^2 S3 - b^2 c^4 S1 S2^2 S3 + \\
a^2 b^2 d^2 S1 S2^2 S3 + b^4 d^2 S1 S2^2 S3 - a^2 c^2 d^2 S1 S2^2 S3 - b^2 c^2 d^2 S1 S2^2 S3 + \\
a^2 b^2 c^2 S0 S1 S3^2 + b^4 c^2 S0 S1 S3^2 - a^2 c^4 S0 S1 S3^2 - b^2 c^4 S0 S1 S3^2 - \\
a^2 b^2 d^2 S0 S1 S3^2 - b^4 d^2 S0 S1 S3^2 + a^2 c^2 d^2 S0 S1 S3^2 + b^2 c^2 d^2 S0 S1 S3^2 + \\
a^2 b^2 c^2 S1^2 S3^2 - b^4 c^2 S1^2 S3^2 - a^2 c^4 S1^2 S3^2 + b^2 c^4 S1^2 S3^2 - a^2 b^2 d^2 S1^2 S3^2 + \\
b^4 d^2 S1^2 S3^2 + a^2 c^2 d^2 S1^2 S3^2 - b^2 c^2 d^2 S1^2 S3^2 + a^2 b^2 c^2 S0 S2 S3^2 - \\
b^4 c^2 S0 S2 S3^2 + a^2 c^4 S0 S2 S3^2 - b^2 c^4 S0 S2 S3^2 - a^2 b^2 d^2 S0 S2 S3^2 + \\
b^4 d^2 S0 S2 S3^2 - a^2 c^2 d^2 S0 S2 S3^2 + b^2 c^2 d^2 S0 S2 S3^2 + a^2 b^2 c^2 S1 S2 S3^2 + \\
b^4 c^2 S1 S2 S3^2 + a^2 c^4 S1 S2 S3^2 + b^2 c^4 S1 S2 S3^2 - a^2 b^2 d^2 S1 S2 S3^2 - \\
b^4 d^2 S1 S2 S3^2 - a^2 c^2 d^2 S1 S2 S3^2 - b^2 c^2 d^2 S1 S2 S3^2 + a^2 b^2 c^2 S0 S1 S2 S4 + \\
b^4 c^2 S0 S1 S2 S4 + a^2 c^4 S0 S1 S2 S4 + b^2 c^4 S0 S1 S2 S4 - a^2 b^2 d^2 S0 S1 S2 S4 - \\
b^4 d^2 S0 S1 S2 S4 - a^2 c^2 d^2 S0 S1 S2 S4 - b^2 c^2 d^2 S0 S1 S2 S4 + a^2 b^2 c^2 S1^2 S2 S4 - \\
b^4 c^2 S1^2 S2 S4 + a^2 c^4 S1^2 S2 S4 - b^2 c^4 S1^2 S2 S4 - a^2 b^2 d^2 S1^2 S2 S4 + \\
b^4 d^2 S1^2 S2 S4 - a^2 c^2 d^2 S1^2 S2 S4 + b^2 c^2 d^2 S1^2 S2 S4 + a^2 b^2 c^2 S0 S2^2 S4 - \\
b^4 c^2 S0 S2^2 S4 - a^2 c^4 S0 S2^2 S4 + b^2 c^4 S0 S2^2 S4 - a^2 b^2 d^2 S0 S2^2 S4 + \\
b^4 d^2 S0 S2^2 S4 + a^2 c^2 d^2 S0 S2^2 S4 - b^2 c^2 d^2 S0 S2^2 S4 + a^2 b^2 c^2 S1 S2^2 S4 + \\
b^4 c^2 S1 S2^2 S4 - a^2 c^4 S1 S2^2 S4 - b^2 c^4 S1 S2^2 S4 - a^2 b^2 d^2 S1 S2^2 S4 - \\
b^4 d^2 S1 S2^2 S4 + a^2 c^2 d^2 S1 S2^2 S4 + b^2 c^2 d^2 S1 S2^2 S4 + a^2 b^2 c^2 S0 S1 S3 S4 + \\
b^4 c^2 S0 S1 S3 S4 - a^2 c^4 S0 S1 S3 S4 - b^2 c^4 S0 S1 S3 S4 + a^2 b^2 d^2 S0 S1 S3 S4 + \\
b^4 d^2 S0 S1 S3 S4 - a^2 c^2 d^2 S0 S1 S3 S4 - b^2 c^2 d^2 S0 S1 S3 S4 + a^2 b^2 c^2 S1^2 S3 S4 - \\
b^4 c^2 S1^2 S3 S4 - a^2 c^4 S1^2 S3 S4 + b^2 c^4 S1^2 S3 S4 + a^2 b^2 d^2 S1^2 S3 S4 - \\
b^4 d^2 S1^2 S3 S4 - a^2 c^2 d^2 S1^2 S3 S4 + b^2 c^2 d^2 S1^2 S3 S4 + a^2 b^2 c^2 S0 S2 S3 S4 - \\
b^4 c^2 S0 S2 S3 S4 + a^2 c^4 S0 S2 S3 S4 - b^2 c^4 S0 S2 S3 S4 + a^2 b^2 d^2 S0 S2 S3 S4 - \\
b^4 d^2 S0 S2 S3 S4 + a^2 c^2 d^2 S0 S2 S3 S4 - b^2 c^2 d^2 S0 S2 S3 S4 + a^2 b^2 c^2 S1 S2 S3 S4 + \\
b^4 c^2 S1 S2 S3 S4 + a^2 c^4 S1 S2 S3 S4 + b^2 c^4 S1 S2 S3 S4 + a^2 b^2 d^2 S1 S2 S3 S4 + \\
b^4 d^2 S1 S2 S3 S4 + a^2 c^2 d^2 S1 S2 S3 S4 + b^2 c^2 d^2 S1 S2 S3 S4)), \\
A4 \rightarrow -((16 a^2 b^2 c^2 E0 S0 S1 S2 S3) / (a^2 b^2 c^2 S0 S1 S2 S3 + b^4 c^2 S0 S1 S2 S3 + \\
a^2 c^4 S0 S1 S2 S3 + b^2 c^4 S0 S1 S2 S3 + a^2 b^2 d^2 S0 S1 S2 S3 + b^4 d^2 S0 S1 S2 S3 + \\
a^2 c^2 d^2 S0 S1 S2 S3 + b^2 c^2 d^2 S0 S1 S2 S3 + a^2 b^2 c^2 S1^2 S2 S3 - b^4 c^2 S1^2 S2 S3 + \\
a^2 c^4 S1^2 S2 S3 - b^2 c^4 S1^2 S2 S3 + a^2 b^2 d^2 S1^2 S2 S3 - b^4 d^2 S1^2 S2 S3 + \\
a^2 c^2 d^2 S1^2 S2 S3 - b^2 c^2 d^2 S1^2 S2 S3 + a^2 b^2 c^2 S0 S2^2 S3 - b^4 c^2 S0 S2^2 S3 - \\
a^2 c^4 S0 S2^2 S3 + b^2 c^4 S0 S2^2 S3 + a^2 b^2 d^2 S0 S2^2 S3 - b^4 d^2 S0 S2^2 S3 - \\
a^2 c^2 d^2 S0 S2^2 S3 + b^2 c^2 d^2 S0 S2^2 S3 + a^2 b^2 c^2 S1 S2^2 S3 + b^4 c^2 S1 S2^2 S3 - \\
a^2 c^4 S1 S2^2 S3 - b^2 c^4 S1 S2^2 S3 + a^2 b^2 d^2 S1 S2^2 S3 + b^4 d^2 S1 S2^2 S3 - \\
a^2 c^2 d^2 S1 S2^2 S3 - b^2 c^2 d^2 S1 S2^2 S3 + a^2 b^2 c^2 S0 S1 S3^2 + b^4 c^2 S0 S1 S3^2 - \\
a^2 c^4 S0 S1 S3^2 - b^2 c^4 S0 S1 S3^2 - a^2 b^2 d^2 S0 S1 S3^2 - b^4 d^2 S0 S1 S3^2 +
\end{array}
$$

$a^2 c^2 d^2 S0 S1 S3^2 + b^2 c^2 d^2 S0 S1 S3^2 + a^2 b^2 c^2 S1^2 S3^2 - b^4 c^2 S1^2 S3^2 -$
$a^2 c^4 S1^2 S3^2 + b^2 c^4 S1^2 S3^2 - a^2 b^2 d^2 S1^2 S3^2 + b^4 d^2 S1^2 S3^2 + a^2 c^2 d^2 S1^2 S3^2 -$
$b^2 c^2 d^2 S1^2 S3^2 + a^2 b^2 c^2 S0 S2 S3^2 - b^4 c^2 S0 S2 S3^2 + a^2 c^4 S0 S2 S3^2 -$
$b^2 c^4 S0 S2 S3^2 - a^2 b^2 d^2 S0 S2 S3^2 + b^4 d^2 S0 S2 S3^2 - a^2 c^2 d^2 S0 S2 S3^2 +$
$b^2 c^2 d^2 S0 S2 S3^2 + a^2 b^2 c^2 S1 S2 S3^2 + b^4 c^2 S1 S2 S3^2 + a^2 c^4 S1 S2 S3^2 +$
$b^2 c^4 S1 S2 S3^2 - a^2 b^2 d^2 S1 S2 S3^2 - b^4 d^2 S1 S2 S3^2 - a^2 c^2 d^2 S1 S2 S3^2 -$
$b^2 c^2 d^2 S1 S2 S3^2 + a^2 b^2 c^2 S0 S1 S2 S4 + b^4 c^2 S0 S1 S2 S4 + a^2 c^4 S0 S1 S2 S4 +$
$b^2 c^4 S0 S1 S2 S4 - a^2 b^2 d^2 S0 S1 S2 S4 - b^4 d^2 S0 S1 S2 S4 - a^2 c^2 d^2 S0 S1 S2 S4 -$
$b^2 c^2 d^2 S0 S1 S2 S4 + a^2 b^2 c^2 S1^2 S2 S4 - b^4 c^2 S1^2 S2 S4 + a^2 c^4 S1^2 S2 S4 -$
$b^2 c^4 S1^2 S2 S4 - a^2 b^2 d^2 S1^2 S2 S4 + b^4 d^2 S1^2 S2 S4 - a^2 c^2 d^2 S1^2 S2 S4 +$
$b^2 c^2 d^2 S1^2 S2 S4 + a^2 b^2 c^2 S0 S2^2 S4 - b^4 c^2 S0 S2^2 S4 - a^2 c^4 S0 S2^2 S4 +$
$b^2 c^4 S0 S2^2 S4 - a^2 b^2 d^2 S0 S2^2 S4 + b^4 d^2 S0 S2^2 S4 + a^2 c^2 d^2 S0 S2^2 S4 -$
$b^2 c^2 d^2 S0 S2^2 S4 + a^2 b^2 c^2 S1 S2^2 S4 + b^4 c^2 S1 S2^2 S4 - a^2 c^4 S1 S2^2 S4 - b^2 c^4 S1 S2^2 S4 -$
$a^2 b^2 d^2 S1 S2^2 S4 - b^4 d^2 S1 S2^2 S4 + a^2 c^2 d^2 S1 S2^2 S4 + b^2 c^2 d^2 S1 S2^2 S4 +$
$a^2 b^2 c^2 S0 S1 S3 S4 + b^4 c^2 S0 S1 S3 S4 - a^2 c^4 S0 S1 S3 S4 - b^2 c^4 S0 S1 S3 S4 +$
$a^2 b^2 d^2 S0 S1 S3 S4 + b^4 d^2 S0 S1 S3 S4 - a^2 c^2 d^2 S0 S1 S3 S4 - b^2 c^2 d^2 S0 S1 S3 S4 +$
$a^2 b^2 c^2 S1^2 S3 S4 - b^4 c^2 S1^2 S3 S4 - a^2 c^4 S1^2 S3 S4 + b^2 c^4 S1^2 S3 S4 +$
$a^2 b^2 d^2 S1^2 S3 S4 - b^4 d^2 S1^2 S3 S4 - a^2 c^2 d^2 S1^2 S3 S4 + b^2 c^2 d^2 S1^2 S3 S4 +$
$a^2 b^2 c^2 S0 S2 S3 S4 - b^4 c^2 S0 S2 S3 S4 + a^2 c^4 S0 S2 S3 S4 - b^2 c^4 S0 S2 S3 S4 +$
$a^2 b^2 d^2 S0 S2 S3 S4 - b^4 d^2 S0 S2 S3 S4 + a^2 c^2 d^2 S0 S2 S3 S4 - b^2 c^2 d^2 S0 S2 S3 S4 +$
$a^2 b^2 c^2 S1 S2 S3 S4 + b^4 c^2 S1 S2 S3 S4 + a^2 c^4 S1 S2 S3 S4 + b^2 c^4 S1 S2 S3 S4 +$
$a^2 b^2 d^2 S1 S2 S3 S4 + b^4 d^2 S1 S2 S3 S4 + a^2 c^2 d^2 S1 S2 S3 S4 + b^2 c^2 d^2 S1 S2 S3 S4))\}\}$

C0 =

$-((a^4 b^2 c^2 E0 S0 S1 S2 S3 + a^2 b^4 c^2 E0 S0 S1 S2 S3 + a^4 c^4 E0 S0 S1 S2 S3 + a^2 b^2 c^4 E0 S0 S1 S2 S3 +$
$a^4 b^2 d^2 E0 S0 S1 S2 S3 + a^2 b^4 d^2 E0 S0 S1 S2 S3 + a^4 c^2 d^2 E0 S0 S1 S2 S3 +$
$a^2 b^2 c^2 d^2 E0 S0 S1 S2 S3 - a^4 b^2 c^2 E0 S1^2 S2 S3 + a^2 b^4 c^2 E0 S1^2 S2 S3 - a^4 c^4 E0 S1^2 S2 S3 +$
$a^2 b^2 c^4 E0 S1^2 S2 S3 - a^4 b^2 d^2 E0 S1^2 S2 S3 + a^2 b^4 d^2 E0 S1^2 S2 S3 - a^4 c^2 d^2 E0 S1^2 S2 S3 +$
$a^2 b^2 c^2 d^2 E0 S1^2 S2 S3 + a^4 b^2 c^2 E0 S0 S2^2 S3 - a^2 b^4 c^2 E0 S0 S2^2 S3 - a^4 c^4 E0 S0 S2^2 S3 +$
$a^2 b^2 c^4 E0 S0 S2^2 S3 + a^4 b^2 d^2 E0 S0 S2^2 S3 - a^2 b^4 d^2 E0 S0 S2^2 S3 - a^4 c^2 d^2 E0 S0 S2^2 S3 +$
$a^2 b^2 c^2 d^2 E0 S0 S2^2 S3 - a^4 b^2 c^2 E0 S1 S2^2 S3 - a^2 b^4 c^2 E0 S1 S2^2 S3 + a^4 c^4 E0 S1 S2^2 S3 +$
$a^2 b^2 c^4 E0 S1 S2^2 S3 - a^4 b^2 d^2 E0 S1 S2^2 S3 - a^2 b^4 d^2 E0 S1 S2^2 S3 + a^4 c^2 d^2 E0 S1 S2^2 S3 +$
$a^2 b^2 c^2 d^2 E0 S1 S2^2 S3 + a^4 b^2 c^2 E0 S0 S1 S3^2 + a^2 b^4 c^2 E0 S0 S1 S3^2 - a^4 c^4 E0 S0 S1 S3^2 -$
$a^2 b^2 c^4 E0 S0 S1 S3^2 - a^4 b^2 d^2 E0 S0 S1 S3^2 - a^2 b^4 d^2 E0 S0 S1 S3^2 + a^4 c^2 d^2 E0 S0 S1 S3^2 +$
$a^2 b^2 c^2 d^2 E0 S0 S1 S3^2 - a^4 b^2 c^2 E0 S1^2 S3^2 + a^2 b^4 c^2 E0 S1^2 S3^2 + a^4 c^4 E0 S1^2 S3^2 -$
$a^2 b^2 c^4 E0 S1^2 S3^2 + a^4 b^2 d^2 E0 S1^2 S3^2 - a^2 b^4 d^2 E0 S1^2 S3^2 - a^4 c^2 d^2 E0 S1^2 S3^2 +$
$a^2 b^2 c^2 d^2 E0 S1^2 S3^2 + a^4 b^2 c^2 E0 S0 S2 S3^2 - a^2 b^4 c^2 E0 S0 S2 S3^2 + a^4 c^4 E0 S0 S2 S3^2 -$
$a^2 b^2 c^4 E0 S0 S2 S3^2 - a^4 b^2 d^2 E0 S0 S2 S3^2 + a^2 b^4 d^2 E0 S0 S2 S3^2 - a^4 c^2 d^2 E0 S0 S2 S3^2 +$
$a^2 b^2 c^2 d^2 E0 S0 S2 S3^2 - a^4 b^2 c^2 E0 S1 S2 S3^2 - a^2 b^4 c^2 E0 S1 S2 S3^2 -$
$a^4 c^4 E0 S1 S2 S3^2 - a^2 b^2 c^4 E0 S1 S2 S3^2 + a^4 b^2 d^2 E0 S1 S2 S3^2 + a^2 b^4 d^2 E0 S1 S2 S3^2 +$
$a^4 c^2 d^2 E0 S1 S2 S3^2 + a^2 b^2 c^2 d^2 E0 S1 S2 S3^2 + a^4 b^2 c^2 E0 S0 S1 S2 S4 +$
$a^2 b^4 c^2 E0 S0 S1 S2 S4 + a^4 c^4 E0 S0 S1 S2 S4 + a^2 b^2 c^4 E0 S0 S1 S2 S4 -$
$a^4 b^2 d^2 E0 S0 S1 S2 S4 - a^2 b^4 d^2 E0 S0 S1 S2 S4 - a^4 c^2 d^2 E0 S0 S1 S2 S4 -$

$a^2 b^2 c^2 d^2 E0\ S0\ S1\ S2\ S4 - a^4 b^2 c^2 E0\ S1^2\ S2\ S4 + a^2 b^4 c^2 E0\ S1^2\ S2\ S4 -$
$a^4 c^4 E0\ S1^2\ S2\ S4 + a^2 b^2 c^4 E0\ S1^2\ S2\ S4 + a^4 b^2 d^2 E0\ S1^2\ S2\ S4 - a^2 b^4 d^2 E0\ S1^2\ S2\ S4 +$
$a^4 c^2 d^2 E0\ S1^2\ S2\ S4 - a^2 b^2 c^2 d^2 E0\ S1^2\ S2\ S4 + a^4 b^2 c^2 E0\ S0\ S2^2\ S4 - a^2 b^4 c^2 E0\ S0\ S2^2\ S4 -$
$a^4 c^4 E0\ S0\ S2^2\ S4 + a^2 b^2 c^4 E0\ S0\ S2^2\ S4 - a^4 b^2 d^2 E0\ S0\ S2^2\ S4 + a^2 b^4 d^2 E0\ S0\ S2^2\ S4 +$
$a^4 c^2 d^2 E0\ S0\ S2^2\ S4 - a^2 b^2 c^2 d^2 E0\ S0\ S2^2\ S4 - a^4 b^2 c^2 E0\ S1\ S2^2\ S4 - a^2 b^4 c^2 E0\ S1\ S2^2\ S4 +$
$a^4 c^4 E0\ S1\ S2^2\ S4 + a^2 b^2 c^4 E0\ S1\ S2^2\ S4 + a^4 b^2 d^2 E0\ S1\ S2^2\ S4 + a^2 b^4 d^2 E0\ S1\ S2^2\ S4 -$
$a^4 c^2 d^2 E0\ S1\ S2^2\ S4 - a^2 b^2 c^2 d^2 E0\ S1\ S2^2\ S4 + a^4 b^2 c^2 E0\ S0\ S1\ S3\ S4 +$
$a^2 b^4 c^2 E0\ S0\ S1\ S3\ S4 - a^4 c^4 E0\ S0\ S1\ S3\ S4 - a^2 b^2 c^4 E0\ S0\ S1\ S3\ S4 +$
$a^4 b^2 d^2 E0\ S0\ S1\ S3\ S4 + a^2 b^4 d^2 E0\ S0\ S1\ S3\ S4 - a^4 c^2 d^2 E0\ S0\ S1\ S3\ S4 -$
$a^2 b^2 c^2 d^2 E0\ S0\ S1\ S3\ S4 - a^4 b^2 c^2 E0\ S1^2\ S3\ S4 + a^2 b^4 c^2 E0\ S1^2\ S3\ S4 +$
$a^4 c^4 E0\ S1^2\ S3\ S4 - a^2 b^2 c^4 E0\ S1^2\ S3\ S4 - a^4 b^2 d^2 E0\ S1^2\ S3\ S4 + a^2 b^4 d^2 E0\ S1^2\ S3\ S4 +$
$a^4 c^2 d^2 E0\ S1^2\ S3\ S4 - a^2 b^2 c^2 d^2 E0\ S1^2\ S3\ S4 + a^4 b^2 c^2 E0\ S0\ S2\ S3\ S4 -$
$a^2 b^4 c^2 E0\ S0\ S2\ S3\ S4 + a^4 c^4 E0\ S0\ S2\ S3\ S4 - a^2 b^2 c^4 E0\ S0\ S2\ S3\ S4 +$
$a^4 b^2 d^2 E0\ S0\ S2\ S3\ S4 - a^2 b^4 d^2 E0\ S0\ S2\ S3\ S4 + a^4 c^2 d^2 E0\ S0\ S2\ S3\ S4 -$
$a^2 b^2 c^2 d^2 E0\ S0\ S2\ S3\ S4 - a^4 b^2 c^2 E0\ S1\ S2\ S3\ S4 - a^2 b^4 c^2 E0\ S1\ S2\ S3\ S4 -$
$a^4 c^4 E0\ S1\ S2\ S3\ S4 - a^2 b^2 c^4 E0\ S1\ S2\ S3\ S4 - a^4 b^2 d^2 E0\ S1\ S2\ S3\ S4 -$
$a^2 b^4 d^2 E0\ S1\ S2\ S3\ S4 - a^4 c^2 d^2 E0\ S1\ S2\ S3\ S4 - a^2 b^2 c^2 d^2 E0\ S1\ S2\ S3\ S4) /$
$(a^2 b^2 c^2 S0\ S1\ S2\ S3 + b^4 c^2 S0\ S1\ S2\ S3 + a^2 c^4 S0\ S1\ S2\ S3 + b^2 c^4 S0\ S1\ S2\ S3 +$
$a^2 b^2 d^2 S0\ S1\ S2\ S3 + b^4 d^2 S0\ S1\ S2\ S3 + a^2 c^2 d^2 S0\ S1\ S2\ S3 + b^2 c^2 d^2 S0\ S1\ S2\ S3 +$
$a^2 b^2 c^2 S1^2\ S2\ S3 - b^4 c^2 S1^2\ S2\ S3 + a^2 c^4 S1^2\ S2\ S3 - b^2 c^4 S1^2\ S2\ S3 + a^2 b^2 d^2 S1^2\ S2\ S3 -$
$b^4 d^2 S1^2\ S2\ S3 + a^2 c^2 d^2 S1^2\ S2\ S3 - b^2 c^2 d^2 S1^2\ S2\ S3 + a^2 b^2 c^2 S0\ S2^2\ S3 -$
$b^4 c^2 S0\ S2^2\ S3 - a^2 c^4 S0\ S2^2\ S3 + b^2 c^4 S0\ S2^2\ S3 + a^2 b^2 d^2 S0\ S2^2\ S3 - b^4 d^2 S0\ S2^2\ S3 -$
$a^2 c^2 d^2 S0\ S2^2\ S3 + b^2 c^2 d^2 S0\ S2^2\ S3 + a^2 b^2 c^2 S1\ S2^2\ S3 + b^4 c^2 S1\ S2^2\ S3 -$
$a^2 c^4 S1\ S2^2\ S3 - b^2 c^4 S1\ S2^2\ S3 + a^2 b^2 d^2 S1\ S2^2\ S3 + b^4 d^2 S1\ S2^2\ S3 - a^2 c^2 d^2 S1\ S2^2\ S3 -$
$b^2 c^2 d^2 S1\ S2^2\ S3 + a^2 b^2 c^2 S0\ S1\ S3^2 + b^4 c^2 S0\ S1\ S3^2 - a^2 c^4 S0\ S1\ S3^2 - b^2 c^4 S0\ S1\ S3^2 -$
$a^2 b^2 d^2 S0\ S1\ S3^2 - b^4 d^2 S0\ S1\ S3^2 + a^2 c^2 d^2 S0\ S1\ S3^2 + b^2 c^2 d^2 S0\ S1\ S3^2 +$
$a^2 b^2 c^2 S1^2\ S3^2 - b^4 c^2 S1^2\ S3^2 - a^2 c^4 S1^2\ S3^2 + b^2 c^4 S1^2\ S3^2 - a^2 b^2 d^2 S1^2\ S3^2 +$
$b^4 d^2 S1^2\ S3^2 + a^2 c^2 d^2 S1^2\ S3^2 - b^2 c^2 d^2 S1^2\ S3^2 + a^2 b^2 c^2 S0\ S2\ S3^2 - b^4 c^2 S0\ S2\ S3^2 +$
$a^2 c^4 S0\ S2\ S3^2 - b^2 c^4 S0\ S2\ S3^2 - a^2 b^2 d^2 S0\ S2\ S3^2 + b^4 d^2 S0\ S2\ S3^2 - a^2 c^2 d^2 S0\ S2\ S3^2 +$
$b^2 c^2 d^2 S0\ S2\ S3^2 + a^2 b^2 c^2 S1\ S2\ S3^2 + b^4 c^2 S1\ S2\ S3^2 + a^2 c^4 S1\ S2\ S3^2 + b^2 c^4 S1\ S2\ S3^2 -$
$a^2 b^2 d^2 S1\ S2\ S3^2 - b^4 d^2 S1\ S2\ S3^2 - a^2 c^2 d^2 S1\ S2\ S3^2 - b^2 c^2 d^2 S1\ S2\ S3^2 +$
$a^2 b^2 c^2 S0\ S1\ S2\ S4 + b^4 c^2 S0\ S1\ S2\ S4 + a^2 c^4 S0\ S1\ S2\ S4 + b^2 c^4 S0\ S1\ S2\ S4 -$
$a^2 b^2 d^2 S0\ S1\ S2\ S4 - b^4 d^2 S0\ S1\ S2\ S4 - a^2 c^2 d^2 S0\ S1\ S2\ S4 - b^2 c^2 d^2 S0\ S1\ S2\ S4 +$
$a^2 b^2 c^2 S1^2\ S2\ S4 - b^4 c^2 S1^2\ S2\ S4 + a^2 c^4 S1^2\ S2\ S4 - b^2 c^4 S1^2\ S2\ S4 - a^2 b^2 d^2 S1^2\ S2\ S4 +$
$b^4 d^2 S1^2\ S2\ S4 - a^2 c^2 d^2 S1^2\ S2\ S4 + b^2 c^2 d^2 S1^2\ S2\ S4 + a^2 b^2 c^2 S0\ S2^2\ S4 -$
$b^4 c^2 S0\ S2^2\ S4 - a^2 c^4 S0\ S2^2\ S4 + b^2 c^4 S0\ S2^2\ S4 - a^2 b^2 d^2 S0\ S2^2\ S4 + b^4 d^2 S0\ S2^2\ S4 +$
$a^2 c^2 d^2 S0\ S2^2\ S4 - b^2 c^2 d^2 S0\ S2^2\ S4 + a^2 b^2 c^2 S1\ S2^2\ S4 + b^4 c^2 S1\ S2^2\ S4 -$
$a^2 c^4 S1\ S2^2\ S4 - b^2 c^4 S1\ S2^2\ S4 - a^2 b^2 d^2 S1\ S2^2\ S4 - b^4 d^2 S1\ S2^2\ S4 + a^2 c^2 d^2 S1\ S2^2\ S4 +$
$b^2 c^2 d^2 S1\ S2^2\ S4 + a^2 b^2 c^2 S0\ S1\ S3\ S4 + b^4 c^2 S0\ S1\ S3\ S4 - a^2 c^4 S0\ S1\ S3\ S4 -$
$b^2 c^4 S0\ S1\ S3\ S4 + a^2 b^2 d^2 S0\ S1\ S3\ S4 + b^4 d^2 S0\ S1\ S3\ S4 - a^2 c^2 d^2 S0\ S1\ S3\ S4 -$
$b^2 c^2 d^2 S0\ S1\ S3\ S4 + a^2 b^2 c^2 S1^2\ S3\ S4 - b^4 c^2 S1^2\ S3\ S4 - a^2 c^4 S1^2\ S3\ S4 + b^2 c^4 S1^2\ S3\ S4 +$
$a^2 b^2 d^2 S1^2\ S3\ S4 - b^4 d^2 S1^2\ S3\ S4 - a^2 c^2 d^2 S1^2\ S3\ S4 + b^2 c^2 d^2 S1^2\ S3\ S4 +$
$a^2 b^2 c^2 S0\ S2\ S3\ S4 - b^4 c^2 S0\ S2\ S3\ S4 + a^2 c^4 S0\ S2\ S3\ S4 - b^2 c^4 S0\ S2\ S3\ S4 +$

$$a^2 b^2 d^2 S0\, S2\, S3\, S4 - b^4 d^2 S0\, S2\, S3\, S4 + a^2 c^2 d^2 S0\, S2\, S3\, S4 - b^2 c^2 d^2 S0\, S2\, S3\, S4 +$$
$$a^2 b^2 c^2 S1\, S2\, S3\, S4 + b^4 c^2 S1\, S2\, S3\, S4 + a^2 c^4 S1\, S2\, S3\, S4 + b^2 c^4 S1\, S2\, S3\, S4 +$$
$$a^2 b^2 d^2 S1\, S2\, S3\, S4 + b^4 d^2 S1\, S2\, S3\, S4 + a^2 c^2 d^2 S1\, S2\, S3\, S4 + b^2 c^2 d^2 S1\, S2\, S3\, S4))$$
$$-((a^4 b^2 c^2 E0\, S0\, S1\, S2\, S3 + a^2 b^4 c^2 E0\, S0\, S1\, S2\, S3 + a^4 c^4 E0\, S0\, S1\, S2\, S3 + a^2 b^2 c^4 E0\, S0\, S1\, S2\, S3 +$$
$$a^4 b^2 d^2 E0\, S0\, S1\, S2\, S3 + a^2 b^4 d^2 E0\, S0\, S1\, S2\, S3 + a^4 c^2 d^2 E0\, S0\, S1\, S2\, S3 +$$
$$a^2 b^2 c^2 d^2 E0\, S0\, S1\, S2\, S3 - a^4 b^2 c^2 E0\, S1^2 S2\, S3 + a^2 b^4 c^2 E0\, S1^2 S2\, S3 - a^4 c^4 E0\, S1^2 S2\, S3 +$$
$$a^2 b^2 c^4 E0\, S1^2 S2\, S3 - a^4 b^2 d^2 E0\, S1^2 S2\, S3 + a^2 b^4 d^2 E0\, S1^2 S2\, S3 - a^4 c^2 d^2 E0\, S1^2 S2\, S3 +$$
$$a^2 b^2 c^2 d^2 E0\, S1^2 S2\, S3 + a^4 b^2 c^2 E0\, S0\, S2^2 S3 - a^2 b^4 c^2 E0\, S0\, S2^2 S3 - a^4 c^4 E0\, S0\, S2^2 S3 +$$
$$a^2 b^2 c^4 E0\, S0\, S2^2 S3 + a^4 b^2 d^2 E0\, S0\, S2^2 S3 - a^2 b^4 d^2 E0\, S0\, S2^2 S3 - a^4 c^2 d^2 E0\, S0\, S2^2 S3 +$$
$$a^2 b^2 c^2 d^2 E0\, S0\, S2^2 S3 - a^4 b^2 c^2 E0\, S1\, S2^2 S3 - a^2 b^4 c^2 E0\, S1\, S2^2 S3 + a^4 c^4 E0\, S1\, S2^2 S3 +$$
$$a^2 b^2 c^4 E0\, S1\, S2^2 S3 - a^4 b^2 d^2 E0\, S1\, S2^2 S3 - a^2 b^4 d^2 E0\, S1\, S2^2 S3 + a^4 c^2 d^2 E0\, S1\, S2^2 S3 +$$
$$a^2 b^2 c^2 d^2 E0\, S1\, S2^2 S3 + a^4 b^2 c^2 E0\, S0\, S1\, S3^2 + a^2 b^4 c^2 E0\, S0\, S1\, S3^2 - a^4 c^4 E0\, S0\, S1\, S3^2 -$$
$$a^2 b^2 c^4 E0\, S0\, S1\, S3^2 - a^4 b^2 d^2 E0\, S0\, S1\, S3^2 - a^2 b^4 d^2 E0\, S0\, S1\, S3^2 + a^4 c^2 d^2 E0\, S0\, S1\, S3^2 +$$
$$a^2 b^2 c^2 d^2 E0\, S0\, S1\, S3^2 - a^4 b^2 c^2 E0\, S1^2 S3^2 + a^2 b^4 c^2 E0\, S1^2 S3^2 + a^4 c^4 E0\, S1^2 S3^2 -$$
$$a^2 b^2 c^4 E0\, S1^2 S3^2 + a^4 b^2 d^2 E0\, S1^2 S3^2 - a^2 b^4 d^2 E0\, S1^2 S3^2 - a^4 c^2 d^2 E0\, S1^2 S3^2 +$$
$$a^2 b^2 c^2 d^2 E0\, S1^2 S3^2 + a^4 b^2 c^2 E0\, S0\, S2\, S3^2 - a^2 b^4 c^2 E0\, S0\, S2\, S3^2 + a^4 c^4 E0\, S0\, S2\, S3^2 -$$
$$a^2 b^2 c^4 E0\, S0\, S2\, S3^2 - a^4 b^2 d^2 E0\, S0\, S2\, S3^2 + a^2 b^4 d^2 E0\, S0\, S2\, S3^2 - a^4 c^2 d^2 E0\, S0\, S2\, S3^2 +$$
$$a^2 b^2 c^2 d^2 E0\, S0\, S2\, S3^2 - a^4 b^2 c^2 E0\, S1\, S2\, S3^2 - a^2 b^4 c^2 E0\, S1\, S2\, S3^2 -$$
$$a^4 c^4 E0\, S1\, S2\, S3^2 - a^2 b^2 c^4 E0\, S1\, S2\, S3^2 + a^4 b^2 d^2 E0\, S1\, S2\, S3^2 + a^2 b^4 d^2 E0\, S1\, S2\, S3^2 +$$
$$a^4 c^2 d^2 E0\, S1\, S2\, S3^2 + a^2 b^2 c^2 d^2 E0\, S1\, S2\, S3^2 + a^4 b^2 c^2 E0\, S0\, S1\, S2\, S4 +$$
$$a^2 b^4 c^2 E0\, S0\, S1\, S2\, S4 + a^4 c^4 E0\, S0\, S1\, S2\, S4 + a^2 b^2 c^4 E0\, S0\, S1\, S2\, S4 -$$
$$a^4 b^2 d^2 E0\, S0\, S1\, S2\, S4 - a^2 b^4 d^2 E0\, S0\, S1\, S2\, S4 - a^4 c^2 d^2 E0\, S0\, S1\, S2\, S4 -$$
$$a^2 b^2 c^2 d^2 E0\, S0\, S1\, S2\, S4 - a^4 b^2 c^2 E0\, S1^2 S2\, S4 + a^2 b^4 c^2 E0\, S1^2 S2\, S4 -$$
$$a^4 c^4 E0\, S1^2 S2\, S4 + a^2 b^2 c^4 E0\, S1^2 S2\, S4 + a^4 b^2 d^2 E0\, S1^2 S2\, S4 - a^2 b^4 d^2 E0\, S1^2 S2\, S4 +$$
$$a^4 c^2 d^2 E0\, S1^2 S2\, S4 - a^2 b^2 c^2 d^2 E0\, S1^2 S2\, S4 + a^4 b^2 c^2 E0\, S0\, S2^2 S4 - a^2 b^4 c^2 E0\, S0\, S2^2 S4 -$$
$$a^4 c^4 E0\, S0\, S2^2 S4 + a^2 b^2 c^4 E0\, S0\, S2^2 S4 - a^4 b^2 d^2 E0\, S0\, S2^2 S4 + a^2 b^4 d^2 E0\, S0\, S2^2 S4 +$$
$$a^4 c^2 d^2 E0\, S0\, S2^2 S4 - a^2 b^2 c^2 d^2 E0\, S0\, S2^2 S4 - a^4 b^2 c^2 E0\, S1\, S2^2 S4 - a^2 b^4 c^2 E0\, S1\, S2^2 S4 +$$
$$a^4 c^4 E0\, S1\, S2^2 S4 + a^2 b^2 c^4 E0\, S1\, S2^2 S4 + a^4 b^2 d^2 E0\, S1\, S2^2 S4 + a^2 b^4 d^2 E0\, S1\, S2^2 S4 -$$
$$a^4 c^2 d^2 E0\, S1\, S2^2 S4 - a^2 b^2 c^2 d^2 E0\, S1\, S2^2 S4 + a^4 b^2 c^2 E0\, S0\, S1\, S3\, S4 +$$
$$a^2 b^4 c^2 E0\, S0\, S1\, S3\, S4 - a^4 c^4 E0\, S0\, S1\, S3\, S4 - a^2 b^2 c^4 E0\, S0\, S1\, S3\, S4 +$$
$$a^4 b^2 d^2 E0\, S0\, S1\, S3\, S4 + a^2 b^4 d^2 E0\, S0\, S1\, S3\, S4 - a^4 c^2 d^2 E0\, S0\, S1\, S3\, S4 -$$
$$a^2 b^2 c^2 d^2 E0\, S0\, S1\, S3\, S4 - a^4 b^2 c^2 E0\, S1^2 S3\, S4 + a^2 b^4 c^2 E0\, S1^2 S3\, S4 +$$
$$a^4 c^4 E0\, S1^2 S3\, S4 - a^2 b^2 c^4 E0\, S1^2 S3\, S4 - a^4 b^2 d^2 E0\, S1^2 S3\, S4 + a^2 b^4 d^2 E0\, S1^2 S3\, S4 +$$
$$a^4 c^2 d^2 E0\, S1^2 S3\, S4 - a^2 b^2 c^2 d^2 E0\, S1^2 S3\, S4 + a^4 b^2 c^2 E0\, S0\, S2\, S3\, S4 -$$
$$a^2 b^4 c^2 E0\, S0\, S2\, S3\, S4 + a^4 c^4 E0\, S0\, S2\, S3\, S4 - a^2 b^2 c^4 E0\, S0\, S2\, S3\, S4 +$$
$$a^4 b^2 d^2 E0\, S0\, S2\, S3\, S4 - a^2 b^4 d^2 E0\, S0\, S2\, S3\, S4 + a^4 c^2 d^2 E0\, S0\, S2\, S3\, S4 -$$
$$a^2 b^2 c^2 d^2 E0\, S0\, S2\, S3\, S4 - a^4 b^2 c^2 E0\, S1\, S2\, S3\, S4 - a^2 b^4 c^2 E0\, S1\, S2\, S3\, S4 -$$
$$a^4 c^4 E0\, S1\, S2\, S3\, S4 - a^2 b^2 c^4 E0\, S1\, S2\, S3\, S4 - a^4 b^2 d^2 E0\, S1\, S2\, S3\, S4 -$$
$$a^2 b^4 d^2 E0\, S1\, S2\, S3\, S4 - a^4 c^2 d^2 E0\, S1\, S2\, S3\, S4 - a^2 b^2 c^2 d^2 E0\, S1\, S2\, S3\, S4) /$$
$$(a^2 b^2 c^2 S0\, S1\, S2\, S3 + b^4 c^2 S0\, S1\, S2\, S3 + a^2 c^4 S0\, S1\, S2\, S3 + b^2 c^4 S0\, S1\, S2\, S3 +$$
$$a^2 b^2 d^2 S0\, S1\, S2\, S3 + b^4 d^2 S0\, S1\, S2\, S3 + a^2 c^2 d^2 S0\, S1\, S2\, S3 + b^2 c^2 d^2 S0\, S1\, S2\, S3 +$$
$$a^2 b^2 c^2 S1^2 S2\, S3 - b^4 c^2 S1^2 S2\, S3 + a^2 c^4 S1^2 S2\, S3 - b^2 c^4 S1^2 S2\, S3 + a^2 b^2 d^2 S1^2 S2\, S3 -$$

$b^4 d^2 S1^2 S2\,S3 + a^2 c^2 d^2 S1^2 S2\,S3 - b^2 c^2 d^2 S1^2 S2\,S3 + a^2 b^2 c^2 S0\,S2^2 S3 -$
$b^4 c^2 S0\,S2^2 S3 - a^2 c^4 S0\,S2^2 S3 + b^2 c^4 S0\,S2^2 S3 + a^2 b^2 d^2 S0\,S2^2 S3 - b^4 d^2 S0\,S2^2 S3 -$
$a^2 c^2 d^2 S0\,S2^2 S3 + b^2 c^2 d^2 S0\,S2^2 S3 + a^2 b^2 c^2 S1\,S2^2 S3 + b^4 c^2 S1\,S2^2 S3 -$
$a^2 c^4 S1\,S2^2 S3 - b^2 c^4 S1\,S2^2 S3 + a^2 b^2 d^2 S1\,S2^2 S3 + b^4 d^2 S1\,S2^2 S3 - a^2 c^2 d^2 S1\,S2^2 S3 -$
$b^2 c^2 d^2 S1\,S2^2 S3 + a^2 b^2 c^2 S0\,S1\,S3^2 + b^4 c^2 S0\,S1\,S3^2 - a^2 c^4 S0\,S1\,S3^2 - b^2 c^4 S0\,S1\,S3^2 -$
$a^2 b^2 d^2 S0\,S1\,S3^2 - b^4 d^2 S0\,S1\,S3^2 + a^2 c^2 d^2 S0\,S1\,S3^2 + b^2 c^2 d^2 S0\,S1\,S3^2 +$
$a^2 b^2 c^2 S1^2 S3^2 - b^4 c^2 S1^2 S3^2 - a^2 c^4 S1^2 S3^2 + b^2 c^4 S1^2 S3^2 - a^2 b^2 d^2 S1^2 S3^2 +$
$b^4 d^2 S1^2 S3^2 + a^2 c^2 d^2 S1^2 S3^2 - b^2 c^2 d^2 S1^2 S3^2 + a^2 b^2 c^2 S0\,S2\,S3^2 - b^4 c^2 S0\,S2\,S3^2 +$
$a^2 c^4 S0\,S2\,S3^2 - b^2 c^4 S0\,S2\,S3^2 - a^2 b^2 d^2 S0\,S2\,S3^2 + b^4 d^2 S0\,S2\,S3^2 - a^2 c^2 d^2 S0\,S2\,S3^2 +$
$b^2 c^2 d^2 S0\,S2\,S3^2 + a^2 b^2 c^2 S1\,S2\,S3^2 + b^4 c^2 S1\,S2\,S3^2 + a^2 c^4 S1\,S2\,S3^2 + b^2 c^4 S1\,S2\,S3^2 -$
$a^2 b^2 d^2 S1\,S2\,S3^2 - b^4 d^2 S1\,S2\,S3^2 - a^2 c^2 d^2 S1\,S2\,S3^2 - b^2 c^2 d^2 S1\,S2\,S3^2 +$
$a^2 b^2 c^2 S0\,S1\,S2\,S4 + b^4 c^2 S0\,S1\,S2\,S4 + a^2 c^4 S0\,S1\,S2\,S4 + b^2 c^4 S0\,S1\,S2\,S4 -$
$a^2 b^2 d^2 S0\,S1\,S2\,S4 - b^4 d^2 S0\,S1\,S2\,S4 - a^2 c^2 d^2 S0\,S1\,S2\,S4 - b^2 c^2 d^2 S0\,S1\,S2\,S4 +$
$a^2 b^2 c^2 S1^2 S2\,S4 - b^4 c^2 S1^2 S2\,S4 + a^2 c^4 S1^2 S2\,S4 - b^2 c^4 S1^2 S2\,S4 - a^2 b^2 d^2 S1^2 S2\,S4 +$
$b^4 d^2 S1^2 S2\,S4 - a^2 c^2 d^2 S1^2 S2\,S4 + b^2 c^2 d^2 S1^2 S2\,S4 + a^2 b^2 c^2 S0\,S2^2 S4 -$
$b^4 c^2 S0\,S2^2 S4 - a^2 c^4 S0\,S2^2 S4 + b^2 c^4 S0\,S2^2 S4 - a^2 b^2 d^2 S0\,S2^2 S4 + b^4 d^2 S0\,S2^2 S4 +$
$a^2 c^2 d^2 S0\,S2^2 S4 - b^2 c^2 d^2 S0\,S2^2 S4 + a^2 b^2 c^2 S1\,S2^2 S4 + b^4 c^2 S1\,S2^2 S4 -$
$a^2 c^4 S1\,S2^2 S4 - b^2 c^4 S1\,S2^2 S4 - a^2 b^2 d^2 S1\,S2^2 S4 - b^4 d^2 S1\,S2^2 S4 + a^2 c^2 d^2 S1\,S2^2 S4 +$
$b^2 c^2 d^2 S1\,S2^2 S4 + a^2 b^2 c^2 S0\,S1\,S3\,S4 + b^4 c^2 S0\,S1\,S3\,S4 - a^2 c^4 S0\,S1\,S3\,S4 -$
$b^2 c^4 S0\,S1\,S3\,S4 + a^2 b^2 d^2 S0\,S1\,S3\,S4 + b^4 d^2 S0\,S1\,S3\,S4 - a^2 c^2 d^2 S0\,S1\,S3\,S4 -$
$b^2 c^2 d^2 S0\,S1\,S3\,S4 + a^2 b^2 c^2 S1^2 S3\,S4 - b^4 c^2 S1^2 S3\,S4 - a^2 c^4 S1^2 S3\,S4 + b^2 c^4 S1^2 S3\,S4 +$
$a^2 b^2 d^2 S1^2 S3\,S4 - b^4 d^2 S1^2 S3\,S4 - a^2 c^2 d^2 S1^2 S3\,S4 + b^2 c^2 d^2 S1^2 S3\,S4 +$
$a^2 b^2 c^2 S0\,S2\,S3\,S4 - b^4 c^2 S0\,S2\,S3\,S4 + a^2 c^4 S0\,S2\,S3\,S4 - b^2 c^4 S0\,S2\,S3\,S4 +$
$a^2 b^2 d^2 S0\,S2\,S3\,S4 - b^4 d^2 S0\,S2\,S3\,S4 + a^2 c^2 d^2 S0\,S2\,S3\,S4 - b^2 c^2 d^2 S0\,S2\,S3\,S4 +$
$a^2 b^2 c^2 S1\,S2\,S3\,S4 + b^4 c^2 S1\,S2\,S3\,S4 + a^2 c^4 S1\,S2\,S3\,S4 + b^2 c^4 S1\,S2\,S3\,S4 +$
$a^2 b^2 d^2 S1\,S2\,S3\,S4 + b^4 d^2 S1\,S2\,S3\,S4 + a^2 c^2 d^2 S1\,S2\,S3\,S4 + b^2 c^2 d^2 S1\,S2\,S3\,S4 \big) \big)$

Simplify[C0]

$\Big(a^2\,E0\,\Big(-b^2\,(S0 + S1)\,\Big(c^2\,(S1 + S2)\,\Big(d^2\,(S2 + S3)\,(S3 - S4) + c^2\,(S2 - S3)\,(S3 + S4)\Big) +$
$b^2\,(S1 - S2)\,\Big(d^2\,(S2 - S3)\,(S3 - S4) + c^2\,(S2 + S3)\,(S3 + S4)\Big)\Big) -$
$a^2\,(S0 - S1)\,\Big(c^2\,(S1 - S2)\,\Big(d^2\,(S2 + S3)\,(S3 - S4) + c^2\,(S2 - S3)\,(S3 + S4)\Big) +$
$b^2\,(S1 + S2)\,\Big(d^2\,(S2 - S3)\,(S3 - S4) + c^2\,(S2 + S3)\,(S3 + S4)\Big)\Big)\Big)\Big) \Big/$
$\Big(b^2\,(S0 - S1)\,\Big(c^2\,(S1 + S2)\,\Big(d^2\,(S2 + S3)\,(S3 - S4) + c^2\,(S2 - S3)\,(S3 + S4)\Big) +$
$b^2\,(S1 - S2)\,\Big(d^2\,(S2 - S3)\,(S3 - S4) + c^2\,(S2 + S3)\,(S3 + S4)\Big)\Big) +$
$a^2\,(S0 + S1)\,\Big(c^2\,(S1 - S2)\,\Big(d^2\,(S2 + S3)\,(S3 - S4) + c^2\,(S2 - S3)\,(S3 + S4)\Big) +$
$b^2\,(S1 + S2)\,\Big(d^2\,(S2 - S3)\,(S3 - S4) + c^2\,(S2 + S3)\,(S3 + S4)\Big)\Big)\Big)$

$$
\begin{aligned}
A1 = -\Bigg(\Bigg(& 2\, E0\, S0 \\
& \Bigg(-\frac{1}{b^3} 2\, S1\, S2 \left(-\frac{2\, S2\, S3 \left(\frac{S3}{d}+\frac{S4}{d}\right)}{c^3} - \left(-\frac{S2}{c^3}+\frac{S3}{c^3}\right)\left(S3\left(\frac{S3}{d}+\frac{S4}{d}\right)+\frac{S3\left(-d\, S3+d\, S4\right)}{c^2}\right)\right) - \\
& \left(-\frac{S1}{b^3}+\frac{S2}{b^3}\right) \\
& \Bigg(S2 \left(-\frac{2\, S2\, S3 \left(\frac{S3}{d}+\frac{S4}{d}\right)}{c^3} - \left(-\frac{S2}{c^3}+\frac{S3}{c^3}\right)\left(S3\left(\frac{S3}{d}+\frac{S4}{d}\right)+\frac{S3\left(-d\, S3+d\, S4\right)}{c^2}\right)\right) + \frac{1}{b^2} \\
& S2 \left(-\frac{2\, S3 \left(-\frac{S2\, S3}{d}-\frac{S2\, S4}{d}\right)}{c} - \left(\frac{S2}{c}+\frac{S3}{c}\right)\left(S3\left(\frac{S3}{d}+\frac{S4}{d}\right)+\frac{S3\left(-d\, S3+d\, S4\right)}{c^2}\right)\right)\Bigg)\Bigg)\Bigg)\Bigg/ \\
& \Bigg(a \Bigg(-\left(-\frac{S0}{a}-\frac{S1}{a}\right)\Bigg(-\frac{1}{b^3} 2\, S1\, S2 \Bigg(-\frac{2\, S2\, S3 \left(\frac{S3}{d}+\frac{S4}{d}\right)}{c^3} - \left(-\frac{S2}{c^3}+\frac{S3}{c^3}\right) \\
& \left(S3\left(\frac{S3}{d}+\frac{S4}{d}\right)+\frac{S3\left(-d\, S3+d\, S4\right)}{c^2}\right)\Bigg) - \left(-\frac{S1}{b^3}+\frac{S2}{b^3}\right) \\
& \Bigg(S2 \left(-\frac{2\, S2\, S3 \left(\frac{S3}{d}+\frac{S4}{d}\right)}{c^3} - \left(-\frac{S2}{c^3}+\frac{S3}{c^3}\right)\left(S3\left(\frac{S3}{d}+\frac{S4}{d}\right)+\frac{S3\left(-d\, S3+d\, S4\right)}{c^2}\right)\right) + \frac{1}{b^2} \\
& S2 \left(-\frac{2\, S3 \left(-\frac{S2\, S3}{d}-\frac{S2\, S4}{d}\right)}{c} - \left(\frac{S2}{c}+\frac{S3}{c}\right)\left(S3\left(\frac{S3}{d}+\frac{S4}{d}\right)+\frac{S3\left(-d\, S3+d\, S4\right)}{c^2}\right)\right)\Bigg)\Bigg) + \\
& \left(-\frac{S0}{a^3}+\frac{S1}{a^3}\right)\Bigg(\frac{1}{b} 2\, S1\, S2 \Bigg(-\frac{2\, S2\, S3 \left(\frac{S3}{d}+\frac{S4}{d}\right)}{c^3} - \left(-\frac{S2}{c^3}+\frac{S3}{c^3}\right)\Bigg(S3\left(\frac{S3}{d}+\frac{S4}{d}\right) + \\
& \frac{S3\left(-d\, S3+d\, S4\right)}{c^2}\Bigg)\Bigg) - \left(\frac{S1}{b}+\frac{S2}{b}\right)\Bigg(S2 \Bigg(-\frac{2\, S2\, S3 \left(\frac{S3}{d}+\frac{S4}{d}\right)}{c^3} - \\
& \left(-\frac{S2}{c^3}+\frac{S3}{c^3}\right)\left(S3\left(\frac{S3}{d}+\frac{S4}{d}\right)+\frac{S3\left(-d\, S3+d\, S4\right)}{c^2}\right)\Bigg) + \frac{1}{b^2} S2 \\
& \left(-\frac{2\, S3 \left(-\frac{S2\, S3}{d}-\frac{S2\, S4}{d}\right)}{c} - \left(\frac{S2}{c}+\frac{S3}{c}\right)\left(S3\left(\frac{S3}{d}+\frac{S4}{d}\right)+\frac{S3\left(-d\, S3+d\, S4\right)}{c^2}\right)\right)\Bigg)\Bigg)\Bigg)\Bigg)
\end{aligned}
$$

$$
-\Bigg(\Bigg(2\,E0\,S0\left(-\frac{1}{b^3}2\,S1\,S2\left(-\frac{2\,S2\,S3\left(\frac{S3}{d}+\frac{S4}{d}\right)}{c^3}-\left(-\frac{S2}{c^3}+\frac{S3}{c^3}\right)\left(S3\left(\frac{S3}{d}+\frac{S4}{d}\right)+\frac{S3\,(-d\,S3+d\,S4)}{c^2}\right)\right)-\left(-\frac{S1}{b^3}+\frac{S2}{b^3}\right)\left(S2\left(-\frac{2\,S2\,S3\left(\frac{S3}{d}+\frac{S4}{d}\right)}{c^3}-\left(-\frac{S2}{c^3}+\frac{S3}{c^3}\right)\left(S3\left(\frac{S3}{d}+\frac{S4}{d}\right)+\frac{S3\,(-d\,S3+d\,S4)}{c^2}\right)\right)+\frac{1}{b^2}S2\left(-\frac{2\,S3\left(-\frac{S2\,S3}{d}-\frac{S2\,S4}{d}\right)}{c}-\left(\frac{S2}{c}+\frac{S3}{c}\right)\left(S3\left(\frac{S3}{d}+\frac{S4}{d}\right)+\frac{S3\,(-d\,S3+d\,S4)}{c^2}\right)\right)\right)\right)\Bigg)\Bigg/
$$

$$
\Bigg(a\Bigg(\left(\frac{S0}{a}+\frac{S1}{a}\right)\left(-\frac{1}{b^3}2\,S1\,S2\left(-\frac{2\,S2\,S3\left(\frac{S3}{d}+\frac{S4}{d}\right)}{c^3}-\left(-\frac{S2}{c^3}+\frac{S3}{c^3}\right)\left(S3\left(\frac{S3}{d}+\frac{S4}{d}\right)+\frac{S3\,(-d\,S3+d\,S4)}{c^2}\right)\right)-\left(-\frac{S1}{b^3}+\frac{S2}{b^3}\right)\left(S2\left(-\frac{2\,S2\,S3\left(\frac{S3}{d}+\frac{S4}{d}\right)}{c^3}-\left(-\frac{S2}{c^3}+\frac{S3}{c^3}\right)\left(S3\left(\frac{S3}{d}+\frac{S4}{d}\right)+\frac{S3\,(-d\,S3+d\,S4)}{c^2}\right)\right)+\frac{1}{b^2}S2\left(-\frac{2\,S3\left(-\frac{S2\,S3}{d}-\frac{S2\,S4}{d}\right)}{c}-\left(\frac{S2}{c}+\frac{S3}{c}\right)\left(S3\left(\frac{S3}{d}+\frac{S4}{d}\right)+\frac{S3\,(-d\,S3+d\,S4)}{c^2}\right)\right)\right)\right)+
$$

$$
\left(-\frac{S0}{a^3}+\frac{S1}{a^3}\right)\left(\frac{1}{b}2\,S1\,S2\left(-\frac{2\,S2\,S3\left(\frac{S3}{d}+\frac{S4}{d}\right)}{c^3}-\left(-\frac{S2}{c^3}+\frac{S3}{c^3}\right)\left(S3\left(\frac{S3}{d}+\frac{S4}{d}\right)+\frac{S3\,(-d\,S3+d\,S4)}{c^2}\right)\right)-\left(\frac{S1}{b}+\frac{S2}{b}\right)\left(S2\left(-\frac{2\,S2\,S3\left(\frac{S3}{d}+\frac{S4}{d}\right)}{c^3}-\left(-\frac{S2}{c^3}+\frac{S3}{c^3}\right)\left(S3\left(\frac{S3}{d}+\frac{S4}{d}\right)+\frac{S3\,(-d\,S3+d\,S4)}{c^2}\right)\right)+\frac{1}{b^2}S2\left(-\frac{2\,S3\left(-\frac{S2\,S3}{d}-\frac{S2\,S4}{d}\right)}{c}-\left(\frac{S2}{c}+\frac{S3}{c}\right)\left(S3\left(\frac{S3}{d}+\frac{S4}{d}\right)+\frac{S3\,(-d\,S3+d\,S4)}{c^2}\right)\right)\right)\right)\Bigg)\Bigg)
$$

```
FullSimplify[A1]
```

$$
-\Big(\Big(2\,a^2\,E0\,S0\,\big(c^2\,(S1-S2)\,\big(d^2\,(S2+S3)\,(S3-S4)+c^2\,(S2-S3)\,(S3+S4)\big)+b^2\,(S1+S2)\,\big(d^2\,(S2-S3)\,(S3-S4)+c^2\,(S2+S3)\,(S3+S4)\big)\big)\Big)\Big/
$$

$$
\Big(b^2\,(S0-S1)\,\big(c^2\,(S1+S2)\,\big(d^2\,(S2+S3)\,(S3-S4)+c^2\,(S2-S3)\,(S3+S4)\big)+b^2\,(S1-S2)\,\big(d^2\,(S2-S3)\,(S3-S4)+c^2\,(S2+S3)\,(S3+S4)\big)\big)+a^2\,(S0+S1)\,\big(c^2\,(S1-S2)\,\big(d^2\,(S2+S3)\,(S3-S4)+c^2\,(S2-S3)\,(S3+S4)\big)+b^2\,(S1+S2)\,\big(d^2\,(S2-S3)\,(S3-S4)+c^2\,(S2+S3)\,(S3+S4)\big)\big)\Big)\Big)
$$

$$
\begin{aligned}
&C1 = \\
&-((2\,E0\,S0\,(a^2 b^4 c^2 S1 S2 S3 + a^2 b^2 c^4 S1 S2 S3 + a^2 b^4 d^2 S1 S2 S3 + a^2 b^2 c^2 d^2 S1 S2 S3 - a^2 b^4 c^2 \\
&S2^2 S3 + a^2 b^2 c^4 S2^2 S3 - a^2 b^4 d^2 S2^2 S3 + a^2 b^2 c^2 d^2 S2^2 S3 + a^2 b^4 c^2 S1 S3^2 - \\
&a^2 b^2 c^4 S1 S3^2 - a^2 b^4 d^2 S1 S3^2 + a^2 b^2 c^2 d^2 S1 S3^2 - a^2 b^4 c^2 S2 S3^2 - a^2 b^2 c^4 S2 S3^2 + \\
&a^2 b^4 d^2 S2 S3^2 + a^2 b^2 c^2 d^2 S2 S3^2 + a^2 b^4 c^2 S1 S2 S4 + a^2 b^2 c^4 S1 S2 S4 - \\
&a^2 b^4 d^2 S1 S2 S4 - a^2 b^2 c^2 d^2 S1 S2 S4 - a^2 b^4 c^2 S2^2 S4 + a^2 b^2 c^4 S2^2 S4 + a^2 b^4 d^2 S2^2 S4 - \\
&a^2 b^2 c^2 d^2 S2^2 S4 + a^2 b^4 c^2 S1 S3 S4 - a^2 b^2 c^4 S1 S3 S4 + a^2 b^4 d^2 S1 S3 S4 - a^2 b^2 c^2 d^2 S1 \\
&S3 S4 - a^2 b^4 c^2 S2 S3 S4 - a^2 b^2 c^4 S2 S3 S4 - a^2 b^4 d^2 S2 S3 S4 - a^2 b^2 c^2 d^2 S2 S3 S4)) / \\
&(a^2 b^2 c^2 S0 S1 S2 S3 + b^4 c^2 S0 S1 S2 S3 + a^2 c^4 S0 S1 S2 S3 + b^2 c^4 S0 S1 S2 S3 + \\
&a^2 b^2 d^2 S0 S1 S2 S3 + b^4 d^2 S0 S1 S2 S3 + a^2 c^2 d^2 S0 S1 S2 S3 + b^2 c^2 d^2 S0 S1 S2 S3 + \\
&a^2 b^2 c^2 S1^2 S2 S3 - b^4 c^2 S1^2 S2 S3 + a^2 c^4 S1^2 S2 S3 - b^2 c^4 S1^2 S2 S3 + a^2 b^2 d^2 S1^2 S2 S3 - \\
&b^4 d^2 S1^2 S2 S3 + a^2 c^2 d^2 S1^2 S2 S3 - b^2 c^2 d^2 S1^2 S2 S3 + a^2 b^2 c^2 S0 S2^2 S3 - \\
&b^4 c^2 S0 S2^2 S3 - a^2 c^4 S0 S2^2 S3 + b^2 c^4 S0 S2^2 S3 + a^2 b^2 d^2 S0 S2^2 S3 - b^4 d^2 S0 S2^2 S3 - \\
&a^2 c^2 d^2 S0 S2^2 S3 + b^2 c^2 d^2 S0 S2^2 S3 + a^2 b^2 c^2 S1 S2^2 S3 + b^4 c^2 S1 S2^2 S3 - \\
&a^2 c^4 S1 S2^2 S3 - b^2 c^4 S1 S2^2 S3 + a^2 b^2 d^2 S1 S2^2 S3 + b^4 d^2 S1 S2^2 S3 - \\
&a^2 c^2 d^2 S1 S2^2 S3 - b^2 c^2 d^2 S1 S2^2 S3 + a^2 b^2 c^2 S0 S1 S3^2 + b^4 c^2 S0 S1 S3^2 - \\
&a^2 c^4 S0 S1 S3^2 - b^2 c^4 S0 S1 S3^2 - a^2 b^2 d^2 S0 S1 S3^2 - b^4 d^2 S0 S1 S3^2 + \\
&a^2 c^2 d^2 S0 S1 S3^2 + b^2 c^2 d^2 S0 S1 S3^2 + a^2 b^2 c^2 S1^2 S3^2 - b^4 c^2 S1^2 S3^2 - a^2 c^4 S1^2 S3^2 + \\
&b^2 c^4 S1^2 S3^2 - a^2 b^2 d^2 S1^2 S3^2 + b^4 d^2 S1^2 S3^2 + a^2 c^2 d^2 S1^2 S3^2 - b^2 c^2 d^2 S1^2 S3^2 + \\
&a^2 b^2 c^2 S0 S2 S3^2 - b^4 c^2 S0 S2 S3^2 + a^2 c^4 S0 S2 S3^2 - b^2 c^4 S0 S2 S3^2 - a^2 b^2 d^2 S0 S2 S3^2 + \\
&b^4 d^2 S0 S2 S3^2 - a^2 c^2 d^2 S0 S2 S3^2 + b^2 c^2 d^2 S0 S2 S3^2 + a^2 b^2 c^2 S1 S2 S3^2 + \\
&b^4 c^2 S1 S2 S3^2 + a^2 c^4 S1 S2 S3^2 + b^2 c^4 S1 S2 S3^2 - a^2 b^2 d^2 S1 S2 S3^2 - b^4 d^2 S1 S2 S3^2 - \\
&a^2 c^2 d^2 S1 S2 S3^2 - b^2 c^2 d^2 S1 S2 S3^2 + a^2 b^2 c^2 S0 S1 S2 S4 + b^4 c^2 S0 S1 S2 S4 + \\
&a^2 c^4 S0 S1 S2 S4 + b^2 c^4 S0 S1 S2 S4 - a^2 b^2 d^2 S0 S1 S2 S4 - b^4 d^2 S0 S1 S2 S4 - \\
&a^2 c^2 d^2 S0 S1 S2 S4 - b^2 c^2 d^2 S0 S1 S2 S4 + a^2 b^2 c^2 S1^2 S2 S4 - b^4 c^2 S1^2 S2 S4 + \\
&a^2 c^4 S1^2 S2 S4 - b^2 c^4 S1^2 S2 S4 - a^2 b^2 d^2 S1^2 S2 S4 + b^4 d^2 S1^2 S2 S4 - a^2 c^2 d^2 S1^2 S2 S4 + \\
&b^2 c^2 d^2 S1^2 S2 S4 + a^2 b^2 c^2 S0 S2^2 S4 - b^4 c^2 S0 S2^2 S4 - a^2 c^4 S0 S2^2 S4 + \\
&b^2 c^4 S0 S2^2 S4 - a^2 b^2 d^2 S0 S2^2 S4 + b^4 d^2 S0 S2^2 S4 + a^2 c^2 d^2 S0 S2^2 S4 - \\
&b^2 c^2 d^2 S0 S2^2 S4 + a^2 b^2 c^2 S1 S2^2 S4 + b^4 c^2 S1 S2^2 S4 - a^2 c^4 S1 S2^2 S4 - b^2 c^4 S1 S2^2 S4 - \\
&a^2 b^2 d^2 S1 S2^2 S4 - b^4 d^2 S1 S2^2 S4 + a^2 c^2 d^2 S1 S2^2 S4 + b^2 c^2 d^2 S1 S2^2 S4 + \\
&a^2 b^2 c^2 S0 S1 S3 S4 + b^4 c^2 S0 S1 S3 S4 - a^2 c^4 S0 S1 S3 S4 - b^2 c^4 S0 S1 S3 S4 + \\
&a^2 b^2 d^2 S0 S1 S3 S4 + b^4 d^2 S0 S1 S3 S4 - a^2 c^2 d^2 S0 S1 S3 S4 - b^2 c^2 d^2 S0 S1 S3 S4 + \\
&a^2 b^2 c^2 S1^2 S3 S4 - b^4 c^2 S1^2 S3 S4 - a^2 c^4 S1^2 S3 S4 + b^2 c^4 S1^2 S3 S4 + \\
&a^2 b^2 d^2 S1^2 S3 S4 - b^4 d^2 S1^2 S3 S4 - a^2 c^2 d^2 S1^2 S3 S4 + b^2 c^2 d^2 S1^2 S3 S4 + \\
&a^2 b^2 c^2 S0 S2 S3 S4 - b^4 c^2 S0 S2 S3 S4 + a^2 c^4 S0 S2 S3 S4 - b^2 c^4 S0 S2 S3 S4 + \\
&a^2 b^2 d^2 S0 S2 S3 S4 - b^4 d^2 S0 S2 S3 S4 + a^2 c^2 d^2 S0 S2 S3 S4 - b^2 c^2 d^2 S0 S2 S3 S4 + \\
&a^2 b^2 c^2 S1 S2 S3 S4 + b^4 c^2 S1 S2 S3 S4 + a^2 c^4 S1 S2 S3 S4 + b^2 c^4 S1 S2 S3 S4 + \\
&a^2 b^2 d^2 S1 S2 S3 S4 + b^4 d^2 S1 S2 S3 S4 + a^2 c^2 d^2 S1 S2 S3 S4 + b^2 c^2 d^2 S1 S2 S3 S4))
\end{aligned}
$$

$$-\,((2\,E0\,S0\,(a^2 b^4 c^2\,S1\,S2\,S3 + a^2 b^2 c^4\,S1\,S2\,S3 + a^2 b^4 d^2\,S1\,S2\,S3 + a^2 b^2 c^2 d^2\,S1\,S2\,S3 -$$
$$a^2 b^4 c^2\,S2^2\,S3 + a^2 b^2 c^4\,S2^2\,S3 - a^2 b^4 d^2\,S2^2\,S3 + a^2 b^2 c^2 d^2\,S2^2\,S3 + a^2 b^4 c^2\,S1\,S3^2 -$$
$$a^2 b^2 c^4\,S1\,S3^2 - a^2 b^4 d^2\,S1\,S3^2 + a^2 b^2 c^2 d^2\,S1\,S3^2 - a^2 b^4 c^2\,S2\,S3^2 - a^2 b^2 c^4\,S2\,S3^2 +$$
$$a^2 b^4 d^2\,S2\,S3^2 + a^2 b^2 c^2 d^2\,S2\,S3^2 + a^2 b^4 c^2\,S1\,S2\,S4 + a^2 b^2 c^4\,S1\,S2\,S4 - a^2 b^4 d^2\,S1\,S2\,S4 -$$
$$a^2 b^2 c^2 d^2\,S1\,S2\,S4 - a^2 b^4 c^2\,S2^2\,S4 + a^2 b^2 c^4\,S2^2\,S4 + a^2 b^4 d^2\,S2^2\,S4 - a^2 b^2 c^2 d^2\,S2^2\,S4 +$$
$$a^2 b^4 c^2\,S1\,S3\,S4 - a^2 b^2 c^4\,S1\,S3\,S4 + a^2 b^4 d^2\,S1\,S3\,S4 - a^2 b^2 c^2 d^2\,S1\,S3\,S4 -$$
$$a^2 b^4 c^2\,S2\,S3\,S4 - a^2 b^2 c^4\,S2\,S3\,S4 - a^2 b^4 d^2\,S2\,S3\,S4 - a^2 b^2 c^2 d^2\,S2\,S3\,S4))\,/$$
$$(a^2 b^2 c^2\,S0\,S1\,S2\,S3 + b^4 c^2\,S0\,S1\,S2\,S3 + a^2 c^4\,S0\,S1\,S2\,S3 + b^2 c^4\,S0\,S1\,S2\,S3 +$$
$$a^2 b^2 d^2\,S0\,S1\,S2\,S3 + b^4 d^2\,S0\,S1\,S2\,S3 + a^2 c^2 d^2\,S0\,S1\,S2\,S3 + b^2 c^2 d^2\,S0\,S1\,S2\,S3 +$$
$$a^2 b^2 c^2\,S1^2\,S2\,S3 - b^4 c^2\,S1^2\,S2\,S3 + a^2 c^4\,S1^2\,S2\,S3 - b^2 c^4\,S1^2\,S2\,S3 +$$
$$a^2 b^2 d^2\,S1^2\,S2\,S3 - b^4 d^2\,S1^2\,S2\,S3 + a^2 c^2 d^2\,S1^2\,S2\,S3 - b^2 c^2 d^2\,S1^2\,S2\,S3 +$$
$$a^2 b^2 c^2\,S0\,S2^2\,S3 - b^4 c^2\,S0\,S2^2\,S3 - a^2 c^4\,S0\,S2^2\,S3 + b^2 c^4\,S0\,S2^2\,S3 + a^2 b^2 d^2\,S0\,S2^2\,S3 -$$
$$b^4 d^2\,S0\,S2^2\,S3 - a^2 c^2 d^2\,S0\,S2^2\,S3 + b^2 c^2 d^2\,S0\,S2^2\,S3 + a^2 b^2 c^2\,S1\,S2^2\,S3 +$$
$$b^4 c^2\,S1\,S2^2\,S3 - a^2 c^4\,S1\,S2^2\,S3 - b^2 c^4\,S1\,S2^2\,S3 + a^2 b^2 d^2\,S1\,S2^2\,S3 +$$
$$b^4 d^2\,S1\,S2^2\,S3 - a^2 c^2 d^2\,S1\,S2^2\,S3 - b^2 c^2 d^2\,S1\,S2^2\,S3 + a^2 b^2 c^2\,S0\,S1\,S3^2 +$$
$$b^4 c^2\,S0\,S1\,S3^2 - a^2 c^4\,S0\,S1\,S3^2 - b^2 c^4\,S0\,S1\,S3^2 - a^2 b^2 d^2\,S0\,S1\,S3^2 - b^4 d^2\,S0\,S1\,S3^2 +$$
$$a^2 c^2 d^2\,S0\,S1\,S3^2 + b^2 c^2 d^2\,S0\,S1\,S3^2 + a^2 b^2 c^2\,S1^2\,S3^2 - b^4 c^2\,S1^2\,S3^2 -$$
$$a^2 c^4\,S1^2\,S3^2 + b^2 c^4\,S1^2\,S3^2 - a^2 b^2 d^2\,S1^2\,S3^2 + b^4 d^2\,S1^2\,S3^2 + a^2 c^2 d^2\,S1^2\,S3^2 -$$
$$b^2 c^2 d^2\,S1^2\,S3^2 + a^2 b^2 c^2\,S0\,S2\,S3^2 - b^4 c^2\,S0\,S2\,S3^2 + a^2 c^4\,S0\,S2\,S3^2 - b^2 c^4\,S0\,S2\,S3^2 -$$
$$a^2 b^2 d^2\,S0\,S2\,S3^2 + b^4 d^2\,S0\,S2\,S3^2 - a^2 c^2 d^2\,S0\,S2\,S3^2 + b^2 c^2 d^2\,S0\,S2\,S3^2 +$$
$$a^2 b^2 c^2\,S1\,S2\,S3^2 + b^4 c^2\,S1\,S2\,S3^2 + a^2 c^4\,S1\,S2\,S3^2 + b^2 c^4\,S1\,S2\,S3^2 -$$
$$a^2 b^2 d^2\,S1\,S2\,S3^2 - b^4 d^2\,S1\,S2\,S3^2 - a^2 c^2 d^2\,S1\,S2\,S3^2 - b^2 c^2 d^2\,S1\,S2\,S3^2 +$$
$$a^2 b^2 c^2\,S0\,S1\,S2\,S4 + b^4 c^2\,S0\,S1\,S2\,S4 + a^2 c^4\,S0\,S1\,S2\,S4 + b^2 c^4\,S0\,S1\,S2\,S4 -$$
$$a^2 b^2 d^2\,S0\,S1\,S2\,S4 - b^4 d^2\,S0\,S1\,S2\,S4 - a^2 c^2 d^2\,S0\,S1\,S2\,S4 - b^2 c^2 d^2\,S0\,S1\,S2\,S4 +$$
$$a^2 b^2 c^2\,S1^2\,S2\,S4 - b^4 c^2\,S1^2\,S2\,S4 + a^2 c^4\,S1^2\,S2\,S4 - b^2 c^4\,S1^2\,S2\,S4 - a^2 b^2 d^2\,S1^2\,S2\,S4 +$$
$$b^4 d^2\,S1^2\,S2\,S4 - a^2 c^2 d^2\,S1^2\,S2\,S4 + b^2 c^2 d^2\,S1^2\,S2\,S4 + a^2 b^2 c^2\,S0\,S2^2\,S4 -$$
$$b^4 c^2\,S0\,S2^2\,S4 - a^2 c^4\,S0\,S2^2\,S4 + b^2 c^4\,S0\,S2^2\,S4 - a^2 b^2 d^2\,S0\,S2^2\,S4 + b^4 d^2\,S0\,S2^2\,S4 +$$
$$a^2 c^2 d^2\,S0\,S2^2\,S4 - b^2 c^2 d^2\,S0\,S2^2\,S4 + a^2 b^2 c^2\,S1\,S2^2\,S4 + b^4 c^2\,S1\,S2^2\,S4 -$$
$$a^2 c^4\,S1\,S2^2\,S4 - b^2 c^4\,S1\,S2^2\,S4 - a^2 b^2 d^2\,S1\,S2^2\,S4 - b^4 d^2\,S1\,S2^2\,S4 + a^2 c^2 d^2\,S1\,S2^2\,S4 +$$
$$b^2 c^2 d^2\,S1\,S2^2\,S4 + a^2 b^2 c^2\,S0\,S1\,S3\,S4 + b^4 c^2\,S0\,S1\,S3\,S4 - a^2 c^4\,S0\,S1\,S3\,S4 -$$
$$b^2 c^4\,S0\,S1\,S3\,S4 + a^2 b^2 d^2\,S0\,S1\,S3\,S4 + b^4 d^2\,S0\,S1\,S3\,S4 - a^2 c^2 d^2\,S0\,S1\,S3\,S4 -$$
$$b^2 c^2 d^2\,S0\,S1\,S3\,S4 + a^2 b^2 c^2\,S1^2\,S3\,S4 - b^4 c^2\,S1^2\,S3\,S4 - a^2 c^4\,S1^2\,S3\,S4 + b^2 c^4\,S1^2\,S3\,S4 +$$
$$a^2 b^2 d^2\,S1^2\,S3\,S4 - b^4 d^2\,S1^2\,S3\,S4 - a^2 c^2 d^2\,S1^2\,S3\,S4 + b^2 c^2 d^2\,S1^2\,S3\,S4 +$$
$$a^2 b^2 c^2\,S0\,S2\,S3\,S4 - b^4 c^2\,S0\,S2\,S3\,S4 + a^2 c^4\,S0\,S2\,S3\,S4 - b^2 c^4\,S0\,S2\,S3\,S4 +$$
$$a^2 b^2 d^2\,S0\,S2\,S3\,S4 - b^4 d^2\,S0\,S2\,S3\,S4 + a^2 c^2 d^2\,S0\,S2\,S3\,S4 - b^2 c^2 d^2\,S0\,S2\,S3\,S4 +$$
$$a^2 b^2 c^2\,S1\,S2\,S3\,S4 + b^4 c^2\,S1\,S2\,S3\,S4 + a^2 c^4\,S1\,S2\,S3\,S4 + b^2 c^4\,S1\,S2\,S3\,S4 +$$
$$a^2 b^2 d^2\,S1\,S2\,S3\,S4 + b^4 d^2\,S1\,S2\,S3\,S4 + a^2 c^2 d^2\,S1\,S2\,S3\,S4 + b^2 c^2 d^2\,S1\,S2\,S3\,S4))$$

```
FullSimplify[C1]
```

```
-((2 a^2 b^2 E0 S0 (c^2 (S1 + S2) (d^2 (S2 + S3) (S3 - S4) + c^2 (S2 - S3) (S3 + S4)) +
        b^2 (S1 - S2) (d^2 (S2 - S3) (S3 - S4) + c^2 (S2 + S3) (S3 + S4)))) /
    (b^2 (S0 - S1) (c^2 (S1 + S2) (d^2 (S2 + S3) (S3 - S4) + c^2 (S2 - S3) (S3 + S4)) +
          b^2 (S1 - S2) (d^2 (S2 - S3) (S3 - S4) + c^2 (S2 + S3) (S3 + S4))) +
      a^2 (S0 + S1) (c^2 (S1 - S2) (d^2 (S2 + S3) (S3 - S4) + c^2 (S2 - S3) (S3 + S4)) +
          b^2 (S1 + S2) (d^2 (S2 - S3) (S3 - S4) + c^2 (S2 + S3) (S3 + S4)))))
```

```
A2 =
 -((4 b^2 E0 S0 (a^2 c^2 S1 S2 S3 + a^2 d^2 S1 S2 S3 + a^2 c^2 S1 S3^2 - a^2 d^2 S1 S3^2 + a^2 c^2 S1 S2 S4 - a^2 d^2
          S1 S2 S4 + a^2 c^2 S1 S3 S4 + a^2 d^2 S1 S3 S4)) /
    (a^2 b^2 c^2 S0 S1 S2 S3 + b^4 c^2 S0 S1 S2 S3 + a^2 c^4 S0 S1 S2 S3 + b^2 c^4 S0 S1 S2 S3 +
      a^2 b^2 d^2 S0 S1 S2 S3 + b^4 d^2 S0 S1 S2 S3 + a^2 c^2 d^2 S0 S1 S2 S3 + b^2 c^2 d^2 S0 S1 S2 S3 +
      a^2 b^2 c^2 S1^2 S2 S3 - b^4 c^2 S1^2 S2 S3 + a^2 c^4 S1^2 S2 S3 - b^2 c^4 S1^2 S2 S3 + a^2 b^2 d^2 S1^2 S2 S3 -
      b^4 d^2 S1^2 S2 S3 + a^2 c^2 d^2 S1^2 S2 S3 - b^2 c^2 d^2 S1^2 S2 S3 + a^2 b^2 c^2 S0 S2^2 S3 -
      b^4 c^2 S0 S2^2 S3 - a^2 c^4 S0 S2^2 S3 + b^2 c^4 S0 S2^2 S3 + a^2 b^2 d^2 S0 S2^2 S3 - b^4 d^2 S0 S2^2 S3 -
      a^2 c^2 d^2 S0 S2^2 S3 + b^2 c^2 d^2 S0 S2^2 S3 + a^2 b^2 c^2 S1 S2^2 S3 + b^4 c^2 S1 S2^2 S3 -
      a^2 c^4 S1 S2^2 S3 - b^2 c^4 S1 S2^2 S3 + a^2 b^2 d^2 S1 S2^2 S3 + b^4 d^2 S1 S2^2 S3 - a^2 c^2 d^2 S1 S2^2 S3 -
      b^2 c^2 d^2 S1 S2^2 S3 + a^2 b^2 c^2 S0 S1 S3^2 + b^4 c^2 S0 S1 S3^2 - a^2 c^4 S0 S1 S3^2 - b^2 c^4 S0 S1 S3^2 -
      a^2 b^2 d^2 S0 S1 S3^2 - b^4 d^2 S0 S1 S3^2 + a^2 c^2 d^2 S0 S1 S3^2 + b^2 c^2 d^2 S0 S1 S3^2 +
      a^2 b^2 c^2 S1^2 S3^2 - b^4 c^2 S1^2 S3^2 - a^2 c^4 S1^2 S3^2 + b^2 c^4 S1^2 S3^2 - a^2 b^2 d^2 S1^2 S3^2 +
      b^4 d^2 S1^2 S3^2 + a^2 c^2 d^2 S1^2 S3^2 - b^2 c^2 d^2 S1^2 S3^2 + a^2 b^2 c^2 S0 S2 S3^2 - b^4 c^2 S0 S2 S3^2 +
      a^2 c^4 S0 S2 S3^2 - b^2 c^4 S0 S2 S3^2 - a^2 b^2 d^2 S0 S2 S3^2 + b^4 d^2 S0 S2 S3^2 - a^2 c^2 d^2 S0 S2 S3^2 +
      b^2 c^2 d^2 S0 S2 S3^2 + a^2 b^2 c^2 S1 S2 S3^2 + b^4 c^2 S1 S2 S3^2 + a^2 c^4 S1 S2 S3^2 + b^2 c^4 S1 S2 S3^2 -
      a^2 b^2 d^2 S1 S2 S3^2 - b^4 d^2 S1 S2 S3^2 - a^2 c^2 d^2 S1 S2 S3^2 - b^2 c^2 d^2 S1 S2 S3^2 +
      a^2 b^2 c^2 S0 S1 S2 S4 + b^4 c^2 S0 S1 S2 S4 + a^2 c^4 S0 S1 S2 S4 + b^2 c^4 S0 S1 S2 S4 -
      a^2 b^2 d^2 S0 S1 S2 S4 - b^4 d^2 S0 S1 S2 S4 - a^2 c^2 d^2 S0 S1 S2 S4 - b^2 c^2 d^2 S0 S1 S2 S4 +
      a^2 b^2 c^2 S1^2 S2 S4 - b^4 c^2 S1^2 S2 S4 + a^2 c^4 S1^2 S2 S4 - b^2 c^4 S1^2 S2 S4 - a^2 b^2 d^2 S1^2 S2 S4 +
      b^4 d^2 S1^2 S2 S4 - a^2 c^2 d^2 S1^2 S2 S4 + b^2 c^2 d^2 S1^2 S2 S4 + a^2 b^2 c^2 S0 S2^2 S4 -
      b^4 c^2 S0 S2^2 S4 - a^2 c^4 S0 S2^2 S4 + b^2 c^4 S0 S2^2 S4 - a^2 b^2 d^2 S0 S2^2 S4 + b^4 d^2 S0 S2^2 S4 +
      a^2 c^2 d^2 S0 S2^2 S4 - b^2 c^2 d^2 S0 S2^2 S4 + a^2 b^2 c^2 S1 S2^2 S4 + b^4 c^2 S1 S2^2 S4 -
      a^2 c^4 S1 S2^2 S4 - b^2 c^4 S1 S2^2 S4 - a^2 b^2 d^2 S1 S2^2 S4 - b^4 d^2 S1 S2^2 S4 + a^2 c^2 d^2 S1 S2^2 S4 +
      b^2 c^2 d^2 S1 S2^2 S4 + a^2 b^2 c^2 S0 S1 S3 S4 + b^4 c^2 S0 S1 S3 S4 - a^2 c^4 S0 S1 S3 S4 -
      b^2 c^4 S0 S1 S3 S4 + a^2 b^2 d^2 S0 S1 S3 S4 + b^4 d^2 S0 S1 S3 S4 - a^2 c^2 d^2 S0 S1 S3 S4 -
      b^2 c^2 d^2 S0 S1 S3 S4 + a^2 b^2 c^2 S1^2 S3 S4 - b^4 c^2 S1^2 S3 S4 - a^2 c^4 S1^2 S3 S4 + b^2 c^4 S1^2 S3 S4 +
      a^2 b^2 d^2 S1^2 S3 S4 - b^4 d^2 S1^2 S3 S4 - a^2 c^2 d^2 S1^2 S3 S4 + b^2 c^2 d^2 S1^2 S3 S4 +
      a^2 b^2 c^2 S0 S2 S3 S4 - b^4 c^2 S0 S2 S3 S4 + a^2 c^4 S0 S2 S3 S4 - b^2 c^4 S0 S2 S3 S4 +
      a^2 b^2 d^2 S0 S2 S3 S4 - b^4 d^2 S0 S2 S3 S4 + a^2 c^2 d^2 S0 S2 S3 S4 - b^2 c^2 d^2 S0 S2 S3 S4 +
      a^2 b^2 c^2 S1 S2 S3 S4 + b^4 c^2 S1 S2 S3 S4 + a^2 c^4 S1 S2 S3 S4 + b^2 c^4 S1 S2 S3 S4 +
      a^2 b^2 d^2 S1 S2 S3 S4 + b^4 d^2 S1 S2 S3 S4 + a^2 c^2 d^2 S1 S2 S3 S4 + b^2 c^2 d^2 S1 S2 S3 S4))
```

$$
\begin{aligned}
&-\Big(\Big(4 b^2 E0\, S0 \big(a^2 c^2 S1\, S2\, S3 + a^2 d^2 S1\, S2\, S3 + a^2 c^2 S1\, S3^2 - \\
&\quad a^2 d^2 S1\, S3^2 + a^2 c^2 S1\, S2\, S4 - a^2 d^2 S1\, S2\, S4 + a^2 c^2 S1\, S3\, S4 + a^2 d^2 S1\, S3\, S4\big)\Big) \Big/ \\
&\Big(a^2 b^2 c^2 S0\, S1\, S2\, S3 + b^4 c^2 S0\, S1\, S2\, S3 + a^2 c^4 S0\, S1\, S2\, S3 + b^2 c^4 S0\, S1\, S2\, S3 + \\
&a^2 b^2 d^2 S0\, S1\, S2\, S3 + b^4 d^2 S0\, S1\, S2\, S3 + a^2 c^2 d^2 S0\, S1\, S2\, S3 + b^2 c^2 d^2 S0\, S1\, S2\, S3 + \\
&a^2 b^2 c^2 S1^2 S2\, S3 - b^4 c^2 S1^2 S2\, S3 + a^2 c^4 S1^2 S2\, S3 - b^2 c^4 S1^2 S2\, S3 + a^2 b^2 d^2 S1^2 S2\, S3 - \\
&b^4 d^2 S1^2 S2\, S3 + a^2 c^2 d^2 S1^2 S2\, S3 - b^2 c^2 d^2 S1^2 S2\, S3 + a^2 b^2 c^2 S0\, S2^2 S3 - \\
&b^4 c^2 S0\, S2^2 S3 - a^2 c^4 S0\, S2^2 S3 + b^2 c^4 S0\, S2^2 S3 + a^2 b^2 d^2 S0\, S2^2 S3 - b^4 d^2 S0\, S2^2 S3 - \\
&a^2 c^2 d^2 S0\, S2^2 S3 + b^2 c^2 d^2 S0\, S2^2 S3 + a^2 b^2 c^2 S1\, S2^2 S3 + b^4 c^2 S1\, S2^2 S3 - \\
&a^2 c^4 S1\, S2^2 S3 - b^2 c^4 S1\, S2^2 S3 + a^2 b^2 d^2 S1\, S2^2 S3 + b^4 d^2 S1\, S2^2 S3 - a^2 c^2 d^2 S1\, S2^2 S3 - \\
&b^2 c^2 d^2 S1\, S2^2 S3 + a^2 b^2 c^2 S0\, S1\, S3^2 + b^4 c^2 S0\, S1\, S3^2 - a^2 c^4 S0\, S1\, S3^2 - b^2 c^4 S0\, S1\, S3^2 - \\
&a^2 b^2 d^2 S0\, S1\, S3^2 - b^4 d^2 S0\, S1\, S3^2 + a^2 c^2 d^2 S0\, S1\, S3^2 + b^2 c^2 d^2 S0\, S1\, S3^2 + \\
&a^2 b^2 c^2 S1^2 S3^2 - b^4 c^2 S1^2 S3^2 - a^2 c^4 S1^2 S3^2 + b^2 c^4 S1^2 S3^2 - a^2 b^2 d^2 S1^2 S3^2 + \\
&b^4 d^2 S1^2 S3^2 + a^2 c^2 d^2 S1^2 S3^2 - b^2 c^2 d^2 S1^2 S3^2 + a^2 b^2 c^2 S0\, S2\, S3^2 - b^4 c^2 S0\, S2\, S3^2 + \\
&a^2 c^4 S0\, S2\, S3^2 - b^2 c^4 S0\, S2\, S3^2 - a^2 b^2 d^2 S0\, S2\, S3^2 + b^4 d^2 S0\, S2\, S3^2 - a^2 c^2 d^2 S0\, S2\, S3^2 + \\
&b^2 c^2 d^2 S0\, S2\, S3^2 + a^2 b^2 c^2 S1\, S2\, S3^2 + b^4 c^2 S1\, S2\, S3^2 + a^2 c^4 S1\, S2\, S3^2 + b^2 c^4 S1\, S2\, S3^2 - \\
&a^2 b^2 d^2 S1\, S2\, S3^2 - b^4 d^2 S1\, S2\, S3^2 - a^2 c^2 d^2 S1\, S2\, S3^2 - b^2 c^2 d^2 S1\, S2\, S3^2 + \\
&a^2 b^2 c^2 S0\, S1\, S2\, S4 + b^4 c^2 S0\, S1\, S2\, S4 + a^2 c^4 S0\, S1\, S2\, S4 + b^2 c^4 S0\, S1\, S2\, S4 - \\
&a^2 b^2 d^2 S0\, S1\, S2\, S4 - b^4 d^2 S0\, S1\, S2\, S4 - a^2 c^2 d^2 S0\, S1\, S2\, S4 - b^2 c^2 d^2 S0\, S1\, S2\, S4 + \\
&a^2 b^2 c^2 S1^2 S2\, S4 - b^4 c^2 S1^2 S2\, S4 + a^2 c^4 S1^2 S2\, S4 - b^2 c^4 S1^2 S2\, S4 - a^2 b^2 d^2 S1^2 S2\, S4 + \\
&b^4 d^2 S1^2 S2\, S4 - a^2 c^2 d^2 S1^2 S2\, S4 + b^2 c^2 d^2 S1^2 S2\, S4 + a^2 b^2 c^2 S0\, S2^2 S4 - \\
&b^4 c^2 S0\, S2^2 S4 - a^2 c^4 S0\, S2^2 S4 + b^2 c^4 S0\, S2^2 S4 - a^2 b^2 d^2 S0\, S2^2 S4 + b^4 d^2 S0\, S2^2 S4 + \\
&a^2 c^2 d^2 S0\, S2^2 S4 - b^2 c^2 d^2 S0\, S2^2 S4 + a^2 b^2 c^2 S1\, S2^2 S4 + b^4 c^2 S1\, S2^2 S4 - \\
&a^2 c^4 S1\, S2^2 S4 - b^2 c^4 S1\, S2^2 S4 - a^2 b^2 d^2 S1\, S2^2 S4 - b^4 d^2 S1\, S2^2 S4 + a^2 c^2 d^2 S1\, S2^2 S4 + \\
&b^2 c^2 d^2 S1\, S2^2 S4 + a^2 b^2 c^2 S0\, S1\, S3\, S4 + b^4 c^2 S0\, S1\, S3\, S4 - a^2 c^4 S0\, S1\, S3\, S4 - \\
&b^2 c^4 S0\, S1\, S3\, S4 + a^2 b^2 d^2 S0\, S1\, S3\, S4 + b^4 d^2 S0\, S1\, S3\, S4 - a^2 c^2 d^2 S0\, S1\, S3\, S4 - \\
&b^2 c^2 d^2 S0\, S1\, S3\, S4 + a^2 b^2 c^2 S1^2 S3\, S4 - b^4 c^2 S1^2 S3\, S4 - a^2 c^4 S1^2 S3\, S4 + b^2 c^4 S1^2 S3\, S4 + \\
&a^2 b^2 d^2 S1^2 S3\, S4 - b^4 d^2 S1^2 S3\, S4 - a^2 c^2 d^2 S1^2 S3\, S4 + b^2 c^2 d^2 S1^2 S3\, S4 + \\
&a^2 b^2 c^2 S0\, S2\, S3\, S4 - b^4 c^2 S0\, S2\, S3\, S4 + a^2 c^4 S0\, S2\, S3\, S4 - b^2 c^4 S0\, S2\, S3\, S4 + \\
&a^2 b^2 d^2 S0\, S2\, S3\, S4 - b^4 d^2 S0\, S2\, S3\, S4 + a^2 c^2 d^2 S0\, S2\, S3\, S4 - b^2 c^2 d^2 S0\, S2\, S3\, S4 + \\
&a^2 b^2 c^2 S1\, S2\, S3\, S4 + b^4 c^2 S1\, S2\, S3\, S4 + a^2 c^4 S1\, S2\, S3\, S4 + b^2 c^4 S1\, S2\, S3\, S4 + \\
&a^2 b^2 d^2 S1\, S2\, S3\, S4 + b^4 d^2 S1\, S2\, S3\, S4 + a^2 c^2 d^2 S1\, S2\, S3\, S4 + b^2 c^2 d^2 S1\, S2\, S3\, S4\Big)\Big)
\end{aligned}
$$

```
FullSimplify[A2]
```

$$
\begin{aligned}
&-\Big(\Big(4 a^2 b^2 E0\, S0\, S1 \big(d^2 (S2 - S3)(S3 - S4) + c^2 (S2 + S3)(S3 + S4)\big)\Big) \Big/ \\
&\Big(b^2 (S0 - S1) \Big(c^2 (S1 + S2) \big(d^2 (S2 + S3)(S3 - S4) + c^2 (S2 - S3)(S3 + S4)\big) + \\
&\quad b^2 (S1 - S2) \big(d^2 (S2 - S3)(S3 - S4) + c^2 (S2 + S3)(S3 + S4)\big)\Big) + \\
&a^2 (S0 + S1) \Big(c^2 (S1 - S2) \big(d^2 (S2 + S3)(S3 - S4) + c^2 (S2 - S3)(S3 + S4)\big) + \\
&\quad b^2 (S1 + S2) \big(d^2 (S2 - S3)(S3 - S4) + c^2 (S2 + S3)(S3 + S4)\big)\Big)\Big)\Big)
\end{aligned}
$$

$$
\begin{aligned}
&C2 = \\
&-\Big(\big(4\,E0\,S0\,\big(a^2 b^2 c^4 S1\,S2\,S3 + a^2 b^2 c^2 d^2 S1\,S2\,S3 - a^2 b^2 c^4 S1\,S3^2 + a^2 b^2 c^2 d^2 S1\,S3^2 + a^2 b^2 c^4 \\
&\quad S1\,S2\,S4 - a^2 b^2 c^2 d^2 S1\,S2\,S4 - a^2 b^2 c^4 S1\,S3\,S4 - a^2 b^2 c^2 d^2 S1\,S3\,S4\big)\big)\Big/ \\
&\big(a^2 b^2 c^2 S0\,S1\,S2\,S3 + b^4 c^2 S0\,S1\,S2\,S3 + a^2 c^4 S0\,S1\,S2\,S3 + b^2 c^4 S0\,S1\,S2\,S3 + \\
&a^2 b^2 d^2 S0\,S1\,S2\,S3 + b^4 d^2 S0\,S1\,S2\,S3 + a^2 c^2 d^2 S0\,S1\,S2\,S3 + b^2 c^2 d^2 S0\,S1\,S2\,S3 + \\
&a^2 b^2 c^2 S1^2 S2\,S3 - b^4 c^2 S1^2 S2\,S3 + a^2 c^4 S1^2 S2\,S3 - b^2 c^4 S1^2 S2\,S3 + a^2 b^2 d^2 S1^2 S2\,S3 - \\
&b^4 d^2 S1^2 S2\,S3 + a^2 c^2 d^2 S1^2 S2\,S3 - b^2 c^2 d^2 S1^2 S2\,S3 + a^2 b^2 c^2 S0\,S2^2 S3 - \\
&b^4 c^2 S0\,S2^2 S3 - a^2 c^4 S0\,S2^2 S3 + b^2 c^4 S0\,S2^2 S3 + a^2 b^2 d^2 S0\,S2^2 S3 - b^4 d^2 S0\,S2^2 S3 - \\
&a^2 c^2 d^2 S0\,S2^2 S3 + b^2 c^2 d^2 S0\,S2^2 S3 + a^2 b^2 c^2 S1\,S2^2 S3 + b^4 c^2 S1\,S2^2 S3 - \\
&a^2 c^4 S1\,S2^2 S3 - b^2 c^4 S1\,S2^2 S3 + a^2 b^2 d^2 S1\,S2^2 S3 + b^4 d^2 S1\,S2^2 S3 - a^2 c^2 d^2 S1\,S2^2 S3 - \\
&b^2 c^2 d^2 S1\,S2^2 S3 + a^2 b^2 c^2 S0\,S1\,S3^2 + b^4 c^2 S0\,S1\,S3^2 - a^2 c^4 S0\,S1\,S3^2 - b^2 c^4 S0\,S1\,S3^2 - \\
&a^2 b^2 d^2 S0\,S1\,S3^2 - b^4 d^2 S0\,S1\,S3^2 + a^2 c^2 d^2 S0\,S1\,S3^2 + b^2 c^2 d^2 S0\,S1\,S3^2 + \\
&a^2 b^2 c^2 S1^2 S3^2 - b^4 c^2 S1^2 S3^2 - a^2 c^4 S1^2 S3^2 + b^2 c^4 S1^2 S3^2 - a^2 b^2 d^2 S1^2 S3^2 + \\
&b^4 d^2 S1^2 S3^2 + a^2 c^2 d^2 S1^2 S3^2 - b^2 c^2 d^2 S1^2 S3^2 + a^2 b^2 c^2 S0\,S2\,S3^2 - b^4 c^2 S0\,S2\,S3^2 + \\
&a^2 c^4 S0\,S2\,S3^2 - b^2 c^4 S0\,S2\,S3^2 - a^2 b^2 d^2 S0\,S2\,S3^2 + b^4 d^2 S0\,S2\,S3^2 - a^2 c^2 d^2 S0\,S2\,S3^2 + \\
&b^2 c^2 d^2 S0\,S2\,S3^2 + a^2 b^2 c^2 S1\,S2\,S3^2 + b^4 c^2 S1\,S2\,S3^2 + a^2 c^4 S1\,S2\,S3^2 + b^2 c^4 S1\,S2\,S3^2 - \\
&a^2 b^2 d^2 S1\,S2\,S3^2 - b^4 d^2 S1\,S2\,S3^2 - a^2 c^2 d^2 S1\,S2\,S3^2 - b^2 c^2 d^2 S1\,S2\,S3^2 + \\
&a^2 b^2 c^2 S0\,S1\,S2\,S4 + b^4 c^2 S0\,S1\,S2\,S4 + a^2 c^4 S0\,S1\,S2\,S4 + b^2 c^4 S0\,S1\,S2\,S4 - \\
&a^2 b^2 d^2 S0\,S1\,S2\,S4 - b^4 d^2 S0\,S1\,S2\,S4 - a^2 c^2 d^2 S0\,S1\,S2\,S4 - b^2 c^2 d^2 S0\,S1\,S2\,S4 + \\
&a^2 b^2 c^2 S1^2 S2\,S4 - b^4 c^2 S1^2 S2\,S4 + a^2 c^4 S1^2 S2\,S4 - b^2 c^4 S1^2 S2\,S4 - a^2 b^2 d^2 S1^2 S2\,S4 + \\
&b^4 d^2 S1^2 S2\,S4 - a^2 c^2 d^2 S1^2 S2\,S4 + b^2 c^2 d^2 S1^2 S2\,S4 + a^2 b^2 c^2 S0\,S2^2 S4 - \\
&b^4 c^2 S0\,S2^2 S4 - a^2 c^4 S0\,S2^2 S4 + b^2 c^4 S0\,S2^2 S4 - a^2 b^2 d^2 S0\,S2^2 S4 + b^4 d^2 S0\,S2^2 S4 + \\
&a^2 c^2 d^2 S0\,S2^2 S4 - b^2 c^2 d^2 S0\,S2^2 S4 + a^2 b^2 c^2 S1\,S2^2 S4 + b^4 c^2 S1\,S2^2 S4 - \\
&a^2 c^4 S1\,S2^2 S4 - b^2 c^4 S1\,S2^2 S4 - a^2 b^2 d^2 S1\,S2^2 S4 - b^4 d^2 S1\,S2^2 S4 + a^2 c^2 d^2 S1\,S2^2 S4 + \\
&b^2 c^2 d^2 S1\,S2^2 S4 + a^2 b^2 c^2 S0\,S1\,S3\,S4 + b^4 c^2 S0\,S1\,S3\,S4 - a^2 c^4 S0\,S1\,S3\,S4 - \\
&b^2 c^4 S0\,S1\,S3\,S4 + a^2 b^2 d^2 S0\,S1\,S3\,S4 + b^4 d^2 S0\,S1\,S3\,S4 - a^2 c^2 d^2 S0\,S1\,S3\,S4 - \\
&b^2 c^2 d^2 S0\,S1\,S3\,S4 + a^2 b^2 c^2 S1^2 S3\,S4 - b^4 c^2 S1^2 S3\,S4 - a^2 c^4 S1^2 S3\,S4 + b^2 c^4 S1^2 S3\,S4 + \\
&a^2 b^2 d^2 S1^2 S3\,S4 - b^4 d^2 S1^2 S3\,S4 - a^2 c^2 d^2 S1^2 S3\,S4 + b^2 c^2 d^2 S1^2 S3\,S4 + \\
&a^2 b^2 c^2 S0\,S2\,S3\,S4 - b^4 c^2 S0\,S2\,S3\,S4 + a^2 c^4 S0\,S2\,S3\,S4 - b^2 c^4 S0\,S2\,S3\,S4 + \\
&a^2 b^2 d^2 S0\,S2\,S3\,S4 - b^4 d^2 S0\,S2\,S3\,S4 + a^2 c^2 d^2 S0\,S2\,S3\,S4 - b^2 c^2 d^2 S0\,S2\,S3\,S4 + \\
&a^2 b^2 c^2 S1\,S2\,S3\,S4 + b^4 c^2 S1\,S2\,S3\,S4 + a^2 c^4 S1\,S2\,S3\,S4 + b^2 c^4 S1\,S2\,S3\,S4 + \\
&a^2 b^2 d^2 S1\,S2\,S3\,S4 + b^4 d^2 S1\,S2\,S3\,S4 + a^2 c^2 d^2 S1\,S2\,S3\,S4 + b^2 c^2 d^2 S1\,S2\,S3\,S4\big)\Big)
\end{aligned}
$$

$$
\begin{aligned}
& -\Big(\big(4\, E0\, S0\, \big(a^2 b^2 c^4 S1\, S2\, S3 + a^2 b^2 c^2 d^2 S1\, S2\, S3 - a^2 b^2 c^4 S1\, S3^2 + a^2 b^2 c^2 d^2 S1\, S3^2 + \\
& \quad a^2 b^2 c^4 S1\, S2\, S4 - a^2 b^2 c^2 d^2 S1\, S2\, S4 - a^2 b^2 c^4 S1\, S3\, S4 - a^2 b^2 c^2 d^2 S1\, S3\, S4\big)\big) \Big/ \\
& \big(a^2 b^2 c^2 S0\, S1\, S2\, S3 + b^4 c^2 S0\, S1\, S2\, S3 + a^2 c^4 S0\, S1\, S2\, S3 + b^2 c^4 S0\, S1\, S2\, S3 + \\
& a^2 b^2 d^2 S0\, S1\, S2\, S3 + b^4 d^2 S0\, S1\, S2\, S3 + a^2 c^2 d^2 S0\, S1\, S2\, S3 + b^2 c^2 d^2 S0\, S1\, S2\, S3 + \\
& a^2 b^2 c^2 S1^2 S2\, S3 - b^4 c^2 S1^2 S2\, S3 + a^2 c^4 S1^2 S2\, S3 - b^2 c^4 S1^2 S2\, S3 + a^2 b^2 d^2 S1^2 S2\, S3 - \\
& b^4 d^2 S1^2 S2\, S3 + a^2 c^2 d^2 S1^2 S2\, S3 - b^2 c^2 d^2 S1^2 S2\, S3 + a^2 b^2 c^2 S0\, S2^2 S3 - \\
& b^4 c^2 S0\, S2^2 S3 - a^2 c^4 S0\, S2^2 S3 + b^2 c^4 S0\, S2^2 S3 + a^2 b^2 d^2 S0\, S2^2 S3 - b^4 d^2 S0\, S2^2 S3 - \\
& a^2 c^2 d^2 S0\, S2^2 S3 + b^2 c^2 d^2 S0\, S2^2 S3 + a^2 b^2 c^2 S1\, S2^2 S3 + b^4 c^2 S1\, S2^2 S3 - \\
& a^2 c^4 S1\, S2^2 S3 - b^2 c^4 S1\, S2^2 S3 + a^2 b^2 d^2 S1\, S2^2 S3 + b^4 d^2 S1\, S2^2 S3 - a^2 c^2 d^2 S1\, S2^2 S3 - \\
& b^2 c^2 d^2 S1\, S2^2 S3 + a^2 b^2 c^2 S0\, S1\, S3^2 + b^4 c^2 S0\, S1\, S3^2 - a^2 c^4 S0\, S1\, S3^2 - b^2 c^4 S0\, S1\, S3^2 - \\
& a^2 b^2 d^2 S0\, S1\, S3^2 - b^4 d^2 S0\, S1\, S3^2 + a^2 c^2 d^2 S0\, S1\, S3^2 + b^2 c^2 d^2 S0\, S1\, S3^2 + \\
& a^2 b^2 c^2 S1^2 S3^2 - b^4 c^2 S1^2 S3^2 - a^2 c^4 S1^2 S3^2 + b^2 c^4 S1^2 S3^2 - a^2 b^2 d^2 S1^2 S3^2 + \\
& b^4 d^2 S1^2 S3^2 + a^2 c^2 d^2 S1^2 S3^2 - b^2 c^2 d^2 S1^2 S3^2 + a^2 b^2 c^2 S0\, S2\, S3^2 - b^4 c^2 S0\, S2\, S3^2 + \\
& a^2 c^4 S0\, S2\, S3^2 - b^2 c^4 S0\, S2\, S3^2 - a^2 b^2 d^2 S0\, S2\, S3^2 + b^4 d^2 S0\, S2\, S3^2 - a^2 c^2 d^2 S0\, S2\, S3^2 + \\
& b^2 c^2 d^2 S0\, S2\, S3^2 + a^2 b^2 c^2 S1\, S2\, S3^2 + b^4 c^2 S1\, S2\, S3^2 + a^2 c^4 S1\, S2\, S3^2 + b^2 c^4 S1\, S2\, S3^2 - \\
& a^2 b^2 d^2 S1\, S2\, S3^2 - b^4 d^2 S1\, S2\, S3^2 - a^2 c^2 d^2 S1\, S2\, S3^2 - b^2 c^2 d^2 S1\, S2\, S3^2 + \\
& a^2 b^2 c^2 S0\, S1\, S2\, S4 + b^4 c^2 S0\, S1\, S2\, S4 + a^2 c^4 S0\, S1\, S2\, S4 + b^2 c^4 S0\, S1\, S2\, S4 - \\
& a^2 b^2 d^2 S0\, S1\, S2\, S4 - b^4 d^2 S0\, S1\, S2\, S4 - a^2 c^2 d^2 S0\, S1\, S2\, S4 - b^2 c^2 d^2 S0\, S1\, S2\, S4 + \\
& a^2 b^2 c^2 S1^2 S2\, S4 - b^4 c^2 S1^2 S2\, S4 + a^2 c^4 S1^2 S2\, S4 - b^2 c^4 S1^2 S2\, S4 - a^2 b^2 d^2 S1^2 S2\, S4 + \\
& b^4 d^2 S1^2 S2\, S4 - a^2 c^2 d^2 S1^2 S2\, S4 + b^2 c^2 d^2 S1^2 S2\, S4 + a^2 b^2 c^2 S0\, S2^2 S4 - \\
& b^4 c^2 S0\, S2^2 S4 - a^2 c^4 S0\, S2^2 S4 + b^2 c^4 S0\, S2^2 S4 - a^2 b^2 d^2 S0\, S2^2 S4 + b^4 d^2 S0\, S2^2 S4 + \\
& a^2 c^2 d^2 S0\, S2^2 S4 - b^2 c^2 d^2 S0\, S2^2 S4 + a^2 b^2 c^2 S1\, S2^2 S4 + b^4 c^2 S1\, S2^2 S4 - \\
& a^2 c^4 S1\, S2^2 S4 - b^2 c^4 S1\, S2^2 S4 - a^2 b^2 d^2 S1\, S2^2 S4 - b^4 d^2 S1\, S2^2 S4 + a^2 c^2 d^2 S1\, S2^2 S4 + \\
& b^2 c^2 d^2 S1\, S2^2 S4 + a^2 b^2 c^2 S0\, S1\, S3\, S4 + b^4 c^2 S0\, S1\, S3\, S4 - a^2 c^4 S0\, S1\, S3\, S4 - \\
& b^2 c^4 S0\, S1\, S3\, S4 + a^2 b^2 d^2 S0\, S1\, S3\, S4 + b^4 d^2 S0\, S1\, S3\, S4 - a^2 c^2 d^2 S0\, S1\, S3\, S4 - \\
& b^2 c^2 d^2 S0\, S1\, S3\, S4 + a^2 b^2 c^2 S1^2 S3\, S4 - b^4 c^2 S1^2 S3\, S4 - a^2 c^4 S1^2 S3\, S4 + b^2 c^4 S1^2 S3\, S4 + \\
& a^2 b^2 d^2 S1^2 S3\, S4 - b^4 d^2 S1^2 S3\, S4 - a^2 c^2 d^2 S1^2 S3\, S4 + b^2 c^2 d^2 S1^2 S3\, S4 + \\
& a^2 b^2 c^2 S0\, S2\, S3\, S4 - b^4 c^2 S0\, S2\, S3\, S4 + a^2 c^4 S0\, S2\, S3\, S4 - b^2 c^4 S0\, S2\, S3\, S4 + \\
& a^2 b^2 d^2 S0\, S2\, S3\, S4 - b^4 d^2 S0\, S2\, S3\, S4 + a^2 c^2 d^2 S0\, S2\, S3\, S4 - b^2 c^2 d^2 S0\, S2\, S3\, S4 + \\
& a^2 b^2 c^2 S1\, S2\, S3\, S4 + b^4 c^2 S1\, S2\, S3\, S4 + a^2 c^4 S1\, S2\, S3\, S4 + b^2 c^4 S1\, S2\, S3\, S4 + \\
& a^2 b^2 d^2 S1\, S2\, S3\, S4 + b^4 d^2 S1\, S2\, S3\, S4 + a^2 c^2 d^2 S1\, S2\, S3\, S4 + b^2 c^2 d^2 S1\, S2\, S3\, S4\big)\Big)
\end{aligned}
$$

FullSimplify[C2]

$$
\begin{aligned}
& -\Big(\big(4\, a^2 b^2 c^2 E0\, S0\, S1\, \big(d^2 (S2 + S3)(S3 - S4) + c^2 (S2 - S3)(S3 + S4)\big)\big) \Big/ \\
& \quad \big(b^2 (S0 - S1)\, \big(c^2 (S1 + S2)\, \big(d^2 (S2 + S3)(S3 - S4) + c^2 (S2 - S3)(S3 + S4)\big) + \\
& \qquad b^2 (S1 - S2)\, \big(d^2 (S2 - S3)(S3 - S4) + c^2 (S2 + S3)(S3 + S4)\big)\big) + \\
& \quad a^2 (S0 + S1)\, \big(c^2 (S1 - S2)\, \big(d^2 (S2 + S3)(S3 - S4) + c^2 (S2 - S3)(S3 + S4)\big) + \\
& \qquad b^2 (S1 + S2)\, \big(d^2 (S2 - S3)(S3 - S4) + c^2 (S2 + S3)(S3 + S4)\big)\big)\big)\Big)
\end{aligned}
$$

$$
\begin{aligned}
&A3 = \\
&-\Big(\big(8\, b^2 c^2\, E0\, S0\, S2\, (a^2 S1\, S3 + a^2 S1\, S4)\big) \Big/ \big(a^2 b^2 c^2 S0\, S1\, S2\, S3 + b^4 c^2 S0\, S1\, S2\, S3 + a^2 c^4 S0\, S1 \\
&\quad S2\, S3 + b^2 c^4 S0\, S1\, S2\, S3 + a^2 b^2 d^2 S0\, S1\, S2\, S3 + b^4 d^2 S0\, S1\, S2\, S3 + a^2 c^2 d^2 S0\, S1\, S2\, S3 + \\
&\quad b^2 c^2 d^2 S0\, S1\, S2\, S3 + a^2 b^2 c^2 S1^2 S2\, S3 - b^4 c^2 S1^2 S2\, S3 + a^2 c^4 S1^2 S2\, S3 - \\
&\quad b^2 c^4 S1^2 S2\, S3 + a^2 b^2 d^2 S1^2 S2\, S3 - b^4 d^2 S1^2 S2\, S3 + a^2 c^2 d^2 S1^2 S2\, S3 - b^2 c^2 d^2 S1^2 S2\, S3 + \\
&\quad a^2 b^2 c^2 S0\, S2^2 S3 - b^4 c^2 S0\, S2^2 S3 - a^2 c^4 S0\, S2^2 S3 + b^2 c^4 S0\, S2^2 S3 + a^2 b^2 d^2 S0\, S2^2 S3 - \\
&\quad b^4 d^2 S0\, S2^2 S3 - a^2 c^2 d^2 S0\, S2^2 S3 + b^2 c^2 d^2 S0\, S2^2 S3 + a^2 b^2 c^2 S1\, S2^2 S3 + \\
&\quad b^4 c^2 S1\, S2^2 S3 - a^2 c^4 S1\, S2^2 S3 - b^2 c^4 S1\, S2^2 S3 + a^2 b^2 d^2 S1\, S2^2 S3 + b^4 d^2 S1\, S2^2 S3 - \\
&\quad a^2 c^2 d^2 S1\, S2^2 S3 - b^2 c^2 d^2 S1\, S2^2 S3 + a^2 b^2 c^2 S0\, S1\, S3^2 + b^4 c^2 S0\, S1\, S3^2 - \\
&\quad a^2 c^4 S0\, S1\, S3^2 - b^2 c^4 S0\, S1\, S3^2 - a^2 b^2 d^2 S0\, S1\, S3^2 - b^4 d^2 S0\, S1\, S3^2 + a^2 c^2 d^2 S0\, S1\, S3^2 + \\
&\quad b^2 c^2 d^2 S0\, S1\, S3^2 + a^2 b^2 c^2 S1^2 S3^2 - b^4 c^2 S1^2 S3^2 - a^2 c^4 S1^2 S3^2 + b^2 c^4 S1^2 S3^2 - \\
&\quad a^2 b^2 d^2 S1^2 S3^2 + b^4 d^2 S1^2 S3^2 + a^2 c^2 d^2 S1^2 S3^2 - b^2 c^2 d^2 S1^2 S3^2 + a^2 b^2 c^2 S0\, S2\, S3^2 - \\
&\quad b^4 c^2 S0\, S2\, S3^2 + a^2 c^4 S0\, S2\, S3^2 - b^2 c^4 S0\, S2\, S3^2 - a^2 b^2 d^2 S0\, S2\, S3^2 + b^4 d^2 S0\, S2\, S3^2 - \\
&\quad a^2 c^2 d^2 S0\, S2\, S3^2 + b^2 c^2 d^2 S0\, S2\, S3^2 + a^2 b^2 c^2 S1\, S2\, S3^2 + b^4 c^2 S1\, S2\, S3^2 + a^2 c^4 S1\, S2\, S3^2 + \\
&\quad b^2 c^4 S1\, S2\, S3^2 - a^2 b^2 d^2 S1\, S2\, S3^2 - b^4 d^2 S1\, S2\, S3^2 - a^2 c^2 d^2 S1\, S2\, S3^2 - b^2 c^2 d^2 S1\, S2\, S3^2 + \\
&\quad a^2 b^2 c^2 S0\, S1\, S2\, S4 + b^4 c^2 S0\, S1\, S2\, S4 + a^2 c^4 S0\, S1\, S2\, S4 + b^2 c^4 S0\, S1\, S2\, S4 - \\
&\quad a^2 b^2 d^2 S0\, S1\, S2\, S4 - b^4 d^2 S0\, S1\, S2\, S4 - a^2 c^2 d^2 S0\, S1\, S2\, S4 - b^2 c^2 d^2 S0\, S1\, S2\, S4 + \\
&\quad a^2 b^2 c^2 S1^2 S2\, S4 - b^4 c^2 S1^2 S2\, S4 + a^2 c^4 S1^2 S2\, S4 - b^2 c^4 S1^2 S2\, S4 - a^2 b^2 d^2 S1^2 S2\, S4 + \\
&\quad b^4 d^2 S1^2 S2\, S4 - a^2 c^2 d^2 S1^2 S2\, S4 + b^2 c^2 d^2 S1^2 S2\, S4 + a^2 b^2 c^2 S0\, S2^2 S4 - \\
&\quad b^4 c^2 S0\, S2^2 S4 - a^2 c^4 S0\, S2^2 S4 + b^2 c^4 S0\, S2^2 S4 - a^2 b^2 d^2 S0\, S2^2 S4 + b^4 d^2 S0\, S2^2 S4 + \\
&\quad a^2 c^2 d^2 S0\, S2^2 S4 - b^2 c^2 d^2 S0\, S2^2 S4 + a^2 b^2 c^2 S1\, S2^2 S4 + b^4 c^2 S1\, S2^2 S4 - \\
&\quad a^2 c^4 S1\, S2^2 S4 - b^2 c^4 S1\, S2^2 S4 - a^2 b^2 d^2 S1\, S2^2 S4 - b^4 d^2 S1\, S2^2 S4 + a^2 c^2 d^2 S1\, S2^2 S4 + \\
&\quad b^2 c^2 d^2 S1\, S2^2 S4 + a^2 b^2 c^2 S0\, S1\, S3\, S4 + b^4 c^2 S0\, S1\, S3\, S4 - a^2 c^4 S0\, S1\, S3\, S4 - \\
&\quad b^2 c^4 S0\, S1\, S3\, S4 + a^2 b^2 d^2 S0\, S1\, S3\, S4 + b^4 d^2 S0\, S1\, S3\, S4 - a^2 c^2 d^2 S0\, S1\, S3\, S4 - \\
&\quad b^2 c^2 d^2 S0\, S1\, S3\, S4 + a^2 b^2 c^2 S1^2 S3\, S4 - b^4 c^2 S1^2 S3\, S4 - a^2 c^4 S1^2 S3\, S4 + b^2 c^4 S1^2 S3\, S4 + \\
&\quad a^2 b^2 d^2 S1^2 S3\, S4 - b^4 d^2 S1^2 S3\, S4 - a^2 c^2 d^2 S1^2 S3\, S4 + b^2 c^2 d^2 S1^2 S3\, S4 + \\
&\quad a^2 b^2 c^2 S0\, S2\, S3\, S4 - b^4 c^2 S0\, S2\, S3\, S4 + a^2 c^4 S0\, S2\, S3\, S4 - b^2 c^4 S0\, S2\, S3\, S4 + \\
&\quad a^2 b^2 d^2 S0\, S2\, S3\, S4 - b^4 d^2 S0\, S2\, S3\, S4 + a^2 c^2 d^2 S0\, S2\, S3\, S4 - b^2 c^2 d^2 S0\, S2\, S3\, S4 + \\
&\quad a^2 b^2 c^2 S1\, S2\, S3\, S4 + b^4 c^2 S1\, S2\, S3\, S4 + a^2 c^4 S1\, S2\, S3\, S4 + b^2 c^4 S1\, S2\, S3\, S4 + \\
&\quad a^2 b^2 d^2 S1\, S2\, S3\, S4 + b^4 d^2 S1\, S2\, S3\, S4 + a^2 c^2 d^2 S1\, S2\, S3\, S4 + b^2 c^2 d^2 S1\, S2\, S3\, S4\big)\Big)
\end{aligned}
$$

$$
\begin{aligned}
&-((8\, b^2 c^2\, E0\, S0\, S2\, (a^2\, S1\, S3 + a^2\, S1\, S4))\, / \\
&(a^2 b^2 c^2\, S0\, S1\, S2\, S3 + b^4 c^2\, S0\, S1\, S2\, S3 + a^2 c^4\, S0\, S1\, S2\, S3 + b^2 c^4\, S0\, S1\, S2\, S3 + \\
&a^2 b^2 d^2\, S0\, S1\, S2\, S3 + b^4 d^2\, S0\, S1\, S2\, S3 + a^2 c^2 d^2\, S0\, S1\, S2\, S3 + b^2 c^2 d^2\, S0\, S1\, S2\, S3 + \\
&a^2 b^2 c^2\, S1^2\, S2\, S3 - b^4 c^2\, S1^2\, S2\, S3 + a^2 c^4\, S1^2\, S2\, S3 - b^2 c^4\, S1^2\, S2\, S3 + a^2 b^2 d^2\, S1^2\, S2\, S3 - \\
&b^4 d^2\, S1^2\, S2\, S3 + a^2 c^2 d^2\, S1^2\, S2\, S3 - b^2 c^2 d^2\, S1^2\, S2\, S3 + a^2 b^2 c^2\, S0\, S2^2\, S3 - \\
&b^4 c^2\, S0\, S2^2\, S3 - a^2 c^4\, S0\, S2^2\, S3 + b^2 c^4\, S0\, S2^2\, S3 + a^2 b^2 d^2\, S0\, S2^2\, S3 - b^4 d^2\, S0\, S2^2\, S3 - \\
&a^2 c^2 d^2\, S0\, S2^2\, S3 + b^2 c^2 d^2\, S0\, S2^2\, S3 + a^2 b^2 c^2\, S1\, S2^2\, S3 + b^4 c^2\, S1\, S2^2\, S3 - \\
&a^2 c^4\, S1\, S2^2\, S3 - b^2 c^4\, S1\, S2^2\, S3 + a^2 b^2 d^2\, S1\, S2^2\, S3 + b^4 d^2\, S1\, S2^2\, S3 - a^2 c^2 d^2\, S1\, S2^2\, S3 - \\
&b^2 c^2 d^2\, S1\, S2^2\, S3 + a^2 b^2 c^2\, S0\, S1\, S3^2 + b^4 c^2\, S0\, S1\, S3^2 - a^2 c^4\, S0\, S1\, S3^2 - b^2 c^4\, S0\, S1\, S3^2 - \\
&a^2 b^2 d^2\, S0\, S1\, S3^2 - b^4 d^2\, S0\, S1\, S3^2 + a^2 c^2 d^2\, S0\, S1\, S3^2 + b^2 c^2 d^2\, S0\, S1\, S3^2 + \\
&a^2 b^2 c^2\, S1^2\, S3^2 - b^4 c^2\, S1^2\, S3^2 - a^2 c^4\, S1^2\, S3^2 + b^2 c^4\, S1^2\, S3^2 - a^2 b^2 d^2\, S1^2\, S3^2 + \\
&b^4 d^2\, S1^2\, S3^2 + a^2 c^2 d^2\, S1^2\, S3^2 - b^2 c^2 d^2\, S1^2\, S3^2 + a^2 b^2 c^2\, S0\, S2\, S3^2 - b^4 c^2\, S0\, S2\, S3^2 + \\
&a^2 c^4\, S0\, S2\, S3^2 - b^2 c^4\, S0\, S2\, S3^2 - a^2 b^2 d^2\, S0\, S2\, S3^2 + b^4 d^2\, S0\, S2\, S3^2 - a^2 c^2 d^2\, S0\, S2\, S3^2 + \\
&b^2 c^2 d^2\, S0\, S2\, S3^2 + a^2 b^2 c^2\, S1\, S2\, S3^2 + b^4 c^2\, S1\, S2\, S3^2 + a^2 c^4\, S1\, S2\, S3^2 + b^2 c^4\, S1\, S2\, S3^2 - \\
&a^2 b^2 d^2\, S1\, S2\, S3^2 - b^4 d^2\, S1\, S2\, S3^2 - a^2 c^2 d^2\, S1\, S2\, S3^2 - b^2 c^2 d^2\, S1\, S2\, S3^2 + \\
&a^2 b^2 c^2\, S0\, S1\, S2\, S4 + b^4 c^2\, S0\, S1\, S2\, S4 + a^2 c^4\, S0\, S1\, S2\, S4 + b^2 c^4\, S0\, S1\, S2\, S4 - \\
&a^2 b^2 d^2\, S0\, S1\, S2\, S4 - b^4 d^2\, S0\, S1\, S2\, S4 - a^2 c^2 d^2\, S0\, S1\, S2\, S4 - b^2 c^2 d^2\, S0\, S1\, S2\, S4 + \\
&a^2 b^2 c^2\, S1^2\, S2\, S4 - b^4 c^2\, S1^2\, S2\, S4 + a^2 c^4\, S1^2\, S2\, S4 - b^2 c^4\, S1^2\, S2\, S4 - a^2 b^2 d^2\, S1^2\, S2\, S4 + \\
&b^4 d^2\, S1^2\, S2\, S4 - a^2 c^2 d^2\, S1^2\, S2\, S4 + b^2 c^2 d^2\, S1^2\, S2\, S4 + a^2 b^2 c^2\, S0\, S2^2\, S4 - \\
&b^4 c^2\, S0\, S2^2\, S4 - a^2 c^4\, S0\, S2^2\, S4 + b^2 c^4\, S0\, S2^2\, S4 - a^2 b^2 d^2\, S0\, S2^2\, S4 + b^4 d^2\, S0\, S2^2\, S4 + \\
&a^2 c^2 d^2\, S0\, S2^2\, S4 - b^2 c^2 d^2\, S0\, S2^2\, S4 + a^2 b^2 c^2\, S1\, S2^2\, S4 + b^4 c^2\, S1\, S2^2\, S4 - \\
&a^2 c^4\, S1\, S2^2\, S4 - b^2 c^4\, S1\, S2^2\, S4 - a^2 b^2 d^2\, S1\, S2^2\, S4 - b^4 d^2\, S1\, S2^2\, S4 + a^2 c^2 d^2\, S1\, S2^2\, S4 + \\
&b^2 c^2 d^2\, S1\, S2^2\, S4 + a^2 b^2 c^2\, S0\, S1\, S3\, S4 + b^4 c^2\, S0\, S1\, S3\, S4 - a^2 c^4\, S0\, S1\, S3\, S4 - \\
&b^2 c^4\, S0\, S1\, S3\, S4 + a^2 b^2 d^2\, S0\, S1\, S3\, S4 + b^4 d^2\, S0\, S1\, S3\, S4 - a^2 c^2 d^2\, S0\, S1\, S3\, S4 - \\
&b^2 c^2 d^2\, S0\, S1\, S3\, S4 + a^2 b^2 c^2\, S1^2\, S3\, S4 - b^4 c^2\, S1^2\, S3\, S4 - a^2 c^4\, S1^2\, S3\, S4 + b^2 c^4\, S1^2\, S3\, S4 + \\
&a^2 b^2 d^2\, S1^2\, S3\, S4 - b^4 d^2\, S1^2\, S3\, S4 - a^2 c^2 d^2\, S1^2\, S3\, S4 + b^2 c^2 d^2\, S1^2\, S3\, S4 + \\
&a^2 b^2 c^2\, S0\, S2\, S3\, S4 - b^4 c^2\, S0\, S2\, S3\, S4 + a^2 c^4\, S0\, S2\, S3\, S4 - b^2 c^4\, S0\, S2\, S3\, S4 + \\
&a^2 b^2 d^2\, S0\, S2\, S3\, S4 - b^4 d^2\, S0\, S2\, S3\, S4 + a^2 c^2 d^2\, S0\, S2\, S3\, S4 - b^2 c^2 d^2\, S0\, S2\, S3\, S4 + \\
&a^2 b^2 c^2\, S1\, S2\, S3\, S4 + b^4 c^2\, S1\, S2\, S3\, S4 + a^2 c^4\, S1\, S2\, S3\, S4 + b^2 c^4\, S1\, S2\, S3\, S4 + \\
&a^2 b^2 d^2\, S1\, S2\, S3\, S4 + b^4 d^2\, S1\, S2\, S3\, S4 + a^2 c^2 d^2\, S1\, S2\, S3\, S4 + b^2 c^2 d^2\, S1\, S2\, S3\, S4))
\end{aligned}
$$

FullSimplify[A3]

$$
\begin{aligned}
&-((8\, a^2 b^2 c^2\, E0\, S0\, S1\, S2\, (S3 + S4))\, / \\
&(b^2\, (S0 - S1)\, (c^2\, (S1 + S2)\, (d^2\, (S2 + S3)\, (S3 - S4) + c^2\, (S2 - S3)\, (S3 + S4)) + \\
&\quad b^2\, (S1 - S2)\, (d^2\, (S2 - S3)\, (S3 - S4) + c^2\, (S2 + S3)\, (S3 + S4))) + \\
&a^2\, (S0 + S1)\, (c^2\, (S1 - S2)\, (d^2\, (S2 + S3)\, (S3 - S4) + c^2\, (S2 - S3)\, (S3 + S4)) + \\
&\quad b^2\, (S1 + S2)\, (d^2\, (S2 - S3)\, (S3 - S4) + c^2\, (S2 + S3)\, (S3 + S4)))))
\end{aligned}
$$

$$
\begin{aligned}
C3 = &-\Big(\big(8\, b^2\, E0\, S0\, S2\, (a^2 c^2 d^2 S1\, S3 - a^2 c^2 d^2 S1\, S4)\big) \Big/ \\
&\big(a^2 b^2 c^2 S0\, S1\, S2\, S3 + b^4 c^2 S0\, S1\, S2\, S3 + a^2 c^4 S0\, S1\, S2\, S3 + b^2 c^4 S0\, S1\, S2\, S3 + \\
&a^2 b^2 d^2 S0\, S1\, S2\, S3 + b^4 d^2 S0\, S1\, S2\, S3 + a^2 c^2 d^2 S0\, S1\, S2\, S3 + b^2 c^2 d^2 S0\, S1\, S2\, S3 + \\
&a^2 b^2 c^2 S1^2 S2\, S3 - b^4 c^2 S1^2 S2\, S3 + a^2 c^4 S1^2 S2\, S3 - b^2 c^4 S1^2 S2\, S3 + a^2 b^2 d^2 S1^2 S2\, S3 - \\
&b^4 d^2 S1^2 S2\, S3 + a^2 c^2 d^2 S1^2 S2\, S3 - b^2 c^2 d^2 S1^2 S2\, S3 + a^2 b^2 c^2 S0\, S2^2 S3 - \\
&b^4 c^2 S0\, S2^2 S3 - a^2 c^4 S0\, S2^2 S3 + b^2 c^4 S0\, S2^2 S3 + a^2 b^2 d^2 S0\, S2^2 S3 - b^4 d^2 S0\, S2^2 S3 - \\
&a^2 c^2 d^2 S0\, S2^2 S3 + b^2 c^2 d^2 S0\, S2^2 S3 + a^2 b^2 c^2 S1\, S2^2 S3 + b^4 c^2 S1\, S2^2 S3 - \\
&a^2 c^4 S1\, S2^2 S3 - b^2 c^4 S1\, S2^2 S3 + a^2 b^2 d^2 S1\, S2^2 S3 + b^4 d^2 S1\, S2^2 S3 - a^2 c^2 d^2 S1\, S2^2 S3 - \\
&b^2 c^2 d^2 S1\, S2^2 S3 + a^2 b^2 c^2 S0\, S1\, S3^2 + b^4 c^2 S0\, S1\, S3^2 - a^2 c^4 S0\, S1\, S3^2 - b^2 c^4 S0\, S1\, S3^2 - \\
&a^2 b^2 d^2 S0\, S1\, S3^2 - b^4 d^2 S0\, S1\, S3^2 + a^2 c^2 d^2 S0\, S1\, S3^2 + b^2 c^2 d^2 S0\, S1\, S3^2 + \\
&a^2 b^2 c^2 S1^2 S3^2 - b^4 c^2 S1^2 S3^2 - a^2 c^4 S1^2 S3^2 + b^2 c^4 S1^2 S3^2 - a^2 b^2 d^2 S1^2 S3^2 + \\
&b^4 d^2 S1^2 S3^2 + a^2 c^2 d^2 S1^2 S3^2 - b^2 c^2 d^2 S1^2 S3^2 + a^2 b^2 c^2 S0\, S2\, S3^2 - b^4 c^2 S0\, S2\, S3^2 + \\
&a^2 c^4 S0\, S2\, S3^2 - b^2 c^4 S0\, S2\, S3^2 - a^2 b^2 d^2 S0\, S2\, S3^2 + b^4 d^2 S0\, S2\, S3^2 - a^2 c^2 d^2 S0\, S2\, S3^2 + \\
&b^2 c^2 d^2 S0\, S2\, S3^2 + a^2 b^2 c^2 S1\, S2\, S3^2 + b^4 c^2 S1\, S2\, S3^2 + a^2 c^4 S1\, S2\, S3^2 + b^2 c^4 S1\, S2\, S3^2 - \\
&a^2 b^2 d^2 S1\, S2\, S3^2 - b^4 d^2 S1\, S2\, S3^2 - a^2 c^2 d^2 S1\, S2\, S3^2 - b^2 c^2 d^2 S1\, S2\, S3^2 + \\
&a^2 b^2 c^2 S0\, S1\, S2\, S4 + b^4 c^2 S0\, S1\, S2\, S4 + a^2 c^4 S0\, S1\, S2\, S4 + b^2 c^4 S0\, S1\, S2\, S4 - \\
&a^2 b^2 d^2 S0\, S1\, S2\, S4 - b^4 d^2 S0\, S1\, S2\, S4 - a^2 c^2 d^2 S0\, S1\, S2\, S4 - b^2 c^2 d^2 S0\, S1\, S2\, S4 + \\
&a^2 b^2 c^2 S1^2 S2\, S4 - b^4 c^2 S1^2 S2\, S4 + a^2 c^4 S1^2 S2\, S4 - b^2 c^4 S1^2 S2\, S4 - a^2 b^2 d^2 S1^2 S2\, S4 + \\
&b^4 d^2 S1^2 S2\, S4 - a^2 c^2 d^2 S1^2 S2\, S4 + b^2 c^2 d^2 S1^2 S2\, S4 + a^2 b^2 c^2 S0\, S2^2 S4 - \\
&b^4 c^2 S0\, S2^2 S4 - a^2 c^4 S0\, S2^2 S4 + b^2 c^4 S0\, S2^2 S4 - a^2 b^2 d^2 S0\, S2^2 S4 + b^4 d^2 S0\, S2^2 S4 + \\
&a^2 c^2 d^2 S0\, S2^2 S4 - b^2 c^2 d^2 S0\, S2^2 S4 + a^2 b^2 c^2 S1\, S2^2 S4 + b^4 c^2 S1\, S2^2 S4 - \\
&a^2 c^4 S1\, S2^2 S4 - b^2 c^4 S1\, S2^2 S4 - a^2 b^2 d^2 S1\, S2^2 S4 - b^4 d^2 S1\, S2^2 S4 + a^2 c^2 d^2 S1\, S2^2 S4 + \\
&b^2 c^2 d^2 S1\, S2^2 S4 + a^2 b^2 c^2 S0\, S1\, S3\, S4 + b^4 c^2 S0\, S1\, S3\, S4 - a^2 c^4 S0\, S1\, S3\, S4 - \\
&b^2 c^4 S0\, S1\, S3\, S4 + a^2 b^2 d^2 S0\, S1\, S3\, S4 + b^4 d^2 S0\, S1\, S3\, S4 - a^2 c^2 d^2 S0\, S1\, S3\, S4 - \\
&b^2 c^2 d^2 S0\, S1\, S3\, S4 + a^2 b^2 c^2 S1^2 S3\, S4 - b^4 c^2 S1^2 S3\, S4 - a^2 c^4 S1^2 S3\, S4 + b^2 c^4 S1^2 S3\, S4 + \\
&a^2 b^2 d^2 S1^2 S3\, S4 - b^4 d^2 S1^2 S3\, S4 - a^2 c^2 d^2 S1^2 S3\, S4 + b^2 c^2 d^2 S1^2 S3\, S4 + \\
&a^2 b^2 c^2 S0\, S2\, S3\, S4 - b^4 c^2 S0\, S2\, S3\, S4 + a^2 c^4 S0\, S2\, S3\, S4 - b^2 c^4 S0\, S2\, S3\, S4 + \\
&a^2 b^2 d^2 S0\, S2\, S3\, S4 - b^4 d^2 S0\, S2\, S3\, S4 + a^2 c^2 d^2 S0\, S2\, S3\, S4 - b^2 c^2 d^2 S0\, S2\, S3\, S4 + \\
&a^2 b^2 c^2 S1\, S2\, S3\, S4 + b^4 c^2 S1\, S2\, S3\, S4 + a^2 c^4 S1\, S2\, S3\, S4 + b^2 c^4 S1\, S2\, S3\, S4 + \\
&a^2 b^2 d^2 S1\, S2\, S3\, S4 + b^4 d^2 S1\, S2\, S3\, S4 + a^2 c^2 d^2 S1\, S2\, S3\, S4 + b^2 c^2 d^2 S1\, S2\, S3\, S4\big)\Big)
\end{aligned}
$$

$$-\left(\left(8\, b^2\, E0\, S0\, S2\, \left(a^2\, c^2\, d^2\, S1\, S3 - a^2\, c^2\, d^2\, S1\, S4\right)\right)\right/$$
$$\left(a^2 b^2 c^2 S0\, S1\, S2\, S3 + b^4 c^2 S0\, S1\, S2\, S3 + a^2 c^4 S0\, S1\, S2\, S3 + b^2 c^4 S0\, S1\, S2\, S3 +\right.$$
$$a^2 b^2 d^2 S0\, S1\, S2\, S3 + b^4 d^2 S0\, S1\, S2\, S3 + a^2 c^2 d^2 S0\, S1\, S2\, S3 + b^2 c^2 d^2 S0\, S1\, S2\, S3 +$$
$$a^2 b^2 c^2 S1^2 S2\, S3 - b^4 c^2 S1^2 S2\, S3 + a^2 c^4 S1^2 S2\, S3 - b^2 c^4 S1^2 S2\, S3 + a^2 b^2 d^2 S1^2 S2\, S3 -$$
$$b^4 d^2 S1^2 S2\, S3 + a^2 c^2 d^2 S1^2 S2\, S3 - b^2 c^2 d^2 S1^2 S2\, S3 + a^2 b^2 c^2 S0\, S2^2 S3 -$$
$$b^4 c^2 S0\, S2^2 S3 - a^2 c^4 S0\, S2^2 S3 + b^2 c^4 S0\, S2^2 S3 + a^2 b^2 d^2 S0\, S2^2 S3 - b^4 d^2 S0\, S2^2 S3 -$$
$$a^2 c^2 d^2 S0\, S2^2 S3 + b^2 c^2 d^2 S0\, S2^2 S3 + a^2 b^2 c^2 S1\, S2^2 S3 + b^4 c^2 S1\, S2^2 S3 -$$
$$a^2 c^4 S1\, S2^2 S3 - b^2 c^4 S1\, S2^2 S3 + a^2 b^2 d^2 S1\, S2^2 S3 + b^4 d^2 S1\, S2^2 S3 - a^2 c^2 d^2 S1\, S2^2 S3 -$$
$$b^2 c^2 d^2 S1\, S2^2 S3 + a^2 b^2 c^2 S0\, S1\, S3^2 + b^4 c^2 S0\, S1\, S3^2 - a^2 c^4 S0\, S1\, S3^2 - b^2 c^4 S0\, S1\, S3^2 -$$
$$a^2 b^2 d^2 S0\, S1\, S3^2 - b^4 d^2 S0\, S1\, S3^2 + a^2 c^2 d^2 S0\, S1\, S3^2 + b^2 c^2 d^2 S0\, S1\, S3^2 +$$
$$a^2 b^2 c^2 S1^2 S3^2 - b^4 c^2 S1^2 S3^2 - a^2 c^4 S1^2 S3^2 + b^2 c^4 S1^2 S3^2 - a^2 b^2 d^2 S1^2 S3^2 +$$
$$b^4 d^2 S1^2 S3^2 + a^2 c^2 d^2 S1^2 S3^2 - b^2 c^2 d^2 S1^2 S3^2 + a^2 b^2 c^2 S0\, S2\, S3^2 - b^4 c^2 S0\, S2\, S3^2 +$$
$$a^2 c^4 S0\, S2\, S3^2 - b^2 c^4 S0\, S2\, S3^2 - a^2 b^2 d^2 S0\, S2\, S3^2 + b^4 d^2 S0\, S2\, S3^2 - a^2 c^2 d^2 S0\, S2\, S3^2 +$$
$$b^2 c^2 d^2 S0\, S2\, S3^2 + a^2 b^2 c^2 S1\, S2\, S3^2 + b^4 c^2 S1\, S2\, S3^2 + a^2 c^4 S1\, S2\, S3^2 + b^2 c^4 S1\, S2\, S3^2 -$$
$$a^2 b^2 d^2 S1\, S2\, S3^2 - b^4 d^2 S1\, S2\, S3^2 - a^2 c^2 d^2 S1\, S2\, S3^2 - b^2 c^2 d^2 S1\, S2\, S3^2 +$$
$$a^2 b^2 c^2 S0\, S1\, S2\, S4 + b^4 c^2 S0\, S1\, S2\, S4 + a^2 c^4 S0\, S1\, S2\, S4 + b^2 c^4 S0\, S1\, S2\, S4 -$$
$$a^2 b^2 d^2 S0\, S1\, S2\, S4 - b^4 d^2 S0\, S1\, S2\, S4 - a^2 c^2 d^2 S0\, S1\, S2\, S4 - b^2 c^2 d^2 S0\, S1\, S2\, S4 +$$
$$a^2 b^2 c^2 S1^2 S2\, S4 - b^4 c^2 S1^2 S2\, S4 + a^2 c^4 S1^2 S2\, S4 - b^2 c^4 S1^2 S2\, S4 - a^2 b^2 d^2 S1^2 S2\, S4 +$$
$$b^4 d^2 S1^2 S2\, S4 - a^2 c^2 d^2 S1^2 S2\, S4 + b^2 c^2 d^2 S1^2 S2\, S4 + a^2 b^2 c^2 S0\, S2^2 S4 -$$
$$b^4 c^2 S0\, S2^2 S4 - a^2 c^4 S0\, S2^2 S4 + b^2 c^4 S0\, S2^2 S4 - a^2 b^2 d^2 S0\, S2^2 S4 + b^4 d^2 S0\, S2^2 S4 +$$
$$a^2 c^2 d^2 S0\, S2^2 S4 - b^2 c^2 d^2 S0\, S2^2 S4 + a^2 b^2 c^2 S1\, S2^2 S4 + b^4 c^2 S1\, S2^2 S4 -$$
$$a^2 c^4 S1\, S2^2 S4 - b^2 c^4 S1\, S2^2 S4 - a^2 b^2 d^2 S1\, S2^2 S4 - b^4 d^2 S1\, S2^2 S4 + a^2 c^2 d^2 S1\, S2^2 S4 +$$
$$b^2 c^2 d^2 S1\, S2^2 S4 + a^2 b^2 c^2 S0\, S1\, S3\, S4 + b^4 c^2 S0\, S1\, S3\, S4 - a^2 c^4 S0\, S1\, S3\, S4 -$$
$$b^2 c^4 S0\, S1\, S3\, S4 + a^2 b^2 d^2 S0\, S1\, S3\, S4 + b^4 d^2 S0\, S1\, S3\, S4 - a^2 c^2 d^2 S0\, S1\, S3\, S4 -$$
$$b^2 c^2 d^2 S0\, S1\, S3\, S4 + a^2 b^2 c^2 S1^2 S3\, S4 - b^4 c^2 S1^2 S3\, S4 - a^2 c^4 S1^2 S3\, S4 + b^2 c^4 S1^2 S3\, S4 +$$
$$a^2 b^2 d^2 S1^2 S3\, S4 - b^4 d^2 S1^2 S3\, S4 - a^2 c^2 d^2 S1^2 S3\, S4 + b^2 c^2 d^2 S1^2 S3\, S4 +$$
$$a^2 b^2 c^2 S0\, S2\, S3\, S4 - b^4 c^2 S0\, S2\, S3\, S4 + a^2 c^4 S0\, S2\, S3\, S4 - b^2 c^4 S0\, S2\, S3\, S4 +$$
$$a^2 b^2 d^2 S0\, S2\, S3\, S4 - b^4 d^2 S0\, S2\, S3\, S4 + a^2 c^2 d^2 S0\, S2\, S3\, S4 - b^2 c^2 d^2 S0\, S2\, S3\, S4 +$$
$$a^2 b^2 c^2 S1\, S2\, S3\, S4 + b^4 c^2 S1\, S2\, S3\, S4 + a^2 c^4 S1\, S2\, S3\, S4 + b^2 c^4 S1\, S2\, S3\, S4 +$$
$$\left.a^2 b^2 d^2 S1\, S2\, S3\, S4 + b^4 d^2 S1\, S2\, S3\, S4 + a^2 c^2 d^2 S1\, S2\, S3\, S4 + b^2 c^2 d^2 S1\, S2\, S3\, S4\right)\Big)$$

```
FullSimplify[C3]
```

$$\left(8\, a^2\, b^2\, c^2\, d^2\, E0\, S0\, S1\, S2\, (-S3 + S4)\right) /$$
$$\Big(b^2 (S0 - S1) \left(c^2 (S1 + S2) \left(d^2 (S2 + S3) (S3 - S4) + c^2 (S2 - S3) (S3 + S4)\right) +\right.$$
$$\left.b^2 (S1 - S2) \left(d^2 (S2 - S3) (S3 - S4) + c^2 (S2 + S3) (S3 + S4)\right)\right) +$$
$$a^2 (S0 + S1) \left(c^2 (S1 - S2) \left(d^2 (S2 + S3) (S3 - S4) + c^2 (S2 - S3) (S3 + S4)\right) +\right.$$
$$\left.b^2 (S1 + S2) \left(d^2 (S2 - S3) (S3 - S4) + c^2 (S2 + S3) (S3 + S4)\right)\right)\Big)$$

$$
\begin{aligned}
&A4 = -((16\, a^2 b^2 c^2\, E0\, S0\, S1\, S2\, S3) / \\
&(a^2 b^2 c^2 S0 S1 S2 S3 + b^4 c^2 S0 S1 S2 S3 + a^2 c^4 S0 S1 S2 S3 + b^2 c^4 S0 S1 S2 S3 + \\
&a^2 b^2 d^2 S0 S1 S2 S3 + b^4 d^2 S0 S1 S2 S3 + a^2 c^2 d^2 S0 S1 S2 S3 + b^2 c^2 d^2 S0 S1 S2 S3 + \\
&a^2 b^2 c^2 S1^2 S2 S3 - b^4 c^2 S1^2 S2 S3 + a^2 c^4 S1^2 S2 S3 - b^2 c^4 S1^2 S2 S3 + a^2 b^2 d^2 S1^2 S2 S3 - \\
&b^4 d^2 S1^2 S2 S3 + a^2 c^2 d^2 S1^2 S2 S3 - b^2 c^2 d^2 S1^2 S2 S3 + a^2 b^2 c^2 S0 S2^2 S3 - \\
&b^4 c^2 S0 S2^2 S3 - a^2 c^4 S0 S2^2 S3 + b^2 c^4 S0 S2^2 S3 + a^2 b^2 d^2 S0 S2^2 S3 - b^4 d^2 S0 S2^2 S3 - \\
&a^2 c^2 d^2 S0 S2^2 S3 + b^2 c^2 d^2 S0 S2^2 S3 + a^2 b^2 c^2 S1 S2^2 S3 + b^4 c^2 S1 S2^2 S3 - \\
&a^2 c^4 S1 S2^2 S3 - b^2 c^4 S1 S2^2 S3 + a^2 b^2 d^2 S1 S2^2 S3 + b^4 d^2 S1 S2^2 S3 - a^2 c^2 d^2 S1 S2^2 S3 - \\
&b^2 c^2 d^2 S1 S2^2 S3 + a^2 b^2 c^2 S0 S1 S3^2 + b^4 c^2 S0 S1 S3^2 - a^2 c^4 S0 S1 S3^2 - b^2 c^4 S0 S1 S3^2 - \\
&a^2 b^2 d^2 S0 S1 S3^2 - b^4 d^2 S0 S1 S3^2 + a^2 c^2 d^2 S0 S1 S3^2 + b^2 c^2 d^2 S0 S1 S3^2 + \\
&a^2 b^2 c^2 S1^2 S3^2 - b^4 c^2 S1^2 S3^2 - a^2 c^4 S1^2 S3^2 + b^2 c^4 S1^2 S3^2 - a^2 b^2 d^2 S1^2 S3^2 + \\
&b^4 d^2 S1^2 S3^2 + a^2 c^2 d^2 S1^2 S3^2 - b^2 c^2 d^2 S1^2 S3^2 + a^2 b^2 c^2 S0 S2 S3^2 - b^4 c^2 S0 S2 S3^2 + \\
&a^2 c^4 S0 S2 S3^2 - b^2 c^4 S0 S2 S3^2 - a^2 b^2 d^2 S0 S2 S3^2 + b^4 d^2 S0 S2 S3^2 - a^2 c^2 d^2 S0 S2 S3^2 + \\
&b^2 c^2 d^2 S0 S2 S3^2 + a^2 b^2 c^2 S1 S2 S3^2 + b^4 c^2 S1 S2 S3^2 + a^2 c^4 S1 S2 S3^2 + b^2 c^4 S1 S2 S3^2 - \\
&a^2 b^2 d^2 S1 S2 S3^2 - b^4 d^2 S1 S2 S3^2 - a^2 c^2 d^2 S1 S2 S3^2 - b^2 c^2 d^2 S1 S2 S3^2 + \\
&a^2 b^2 c^2 S0 S1 S2 S4 + b^4 c^2 S0 S1 S2 S4 + a^2 c^4 S0 S1 S2 S4 + b^2 c^4 S0 S1 S2 S4 - \\
&a^2 b^2 d^2 S0 S1 S2 S4 - b^4 d^2 S0 S1 S2 S4 - a^2 c^2 d^2 S0 S1 S2 S4 - b^2 c^2 d^2 S0 S1 S2 S4 + \\
&a^2 b^2 c^2 S1^2 S2 S4 - b^4 c^2 S1^2 S2 S4 + a^2 c^4 S1^2 S2 S4 - b^2 c^4 S1^2 S2 S4 - a^2 b^2 d^2 S1^2 S2 S4 + \\
&b^4 d^2 S1^2 S2 S4 - a^2 c^2 d^2 S1^2 S2 S4 + b^2 c^2 d^2 S1^2 S2 S4 + a^2 b^2 c^2 S0 S2^2 S4 - \\
&b^4 c^2 S0 S2^2 S4 - a^2 c^4 S0 S2^2 S4 + b^2 c^4 S0 S2^2 S4 - a^2 b^2 d^2 S0 S2^2 S4 + b^4 d^2 S0 S2^2 S4 + \\
&a^2 c^2 d^2 S0 S2^2 S4 - b^2 c^2 d^2 S0 S2^2 S4 + a^2 b^2 c^2 S1 S2^2 S4 + b^4 c^2 S1 S2^2 S4 - \\
&a^2 c^4 S1 S2^2 S4 - b^2 c^4 S1 S2^2 S4 - a^2 b^2 d^2 S1 S2^2 S4 - b^4 d^2 S1 S2^2 S4 + a^2 c^2 d^2 S1 S2^2 S4 + \\
&b^2 c^2 d^2 S1 S2^2 S4 + a^2 b^2 c^2 S0 S1 S3 S4 + b^4 c^2 S0 S1 S3 S4 - a^2 c^4 S0 S1 S3 S4 - \\
&b^2 c^4 S0 S1 S3 S4 + a^2 b^2 d^2 S0 S1 S3 S4 + b^4 d^2 S0 S1 S3 S4 - a^2 c^2 d^2 S0 S1 S3 S4 - \\
&b^2 c^2 d^2 S0 S1 S3 S4 + a^2 b^2 c^2 S1^2 S3 S4 - b^4 c^2 S1^2 S3 S4 - a^2 c^4 S1^2 S3 S4 + b^2 c^4 S1^2 S3 S4 + \\
&a^2 b^2 d^2 S1^2 S3 S4 - b^4 d^2 S1^2 S3 S4 - a^2 c^2 d^2 S1^2 S3 S4 + b^2 c^2 d^2 S1^2 S3 S4 + \\
&a^2 b^2 c^2 S0 S2 S3 S4 - b^4 c^2 S0 S2 S3 S4 + a^2 c^4 S0 S2 S3 S4 - b^2 c^4 S0 S2 S3 S4 + \\
&a^2 b^2 d^2 S0 S2 S3 S4 - b^4 d^2 S0 S2 S3 S4 + a^2 c^2 d^2 S0 S2 S3 S4 - b^2 c^2 d^2 S0 S2 S3 S4 + \\
&a^2 b^2 c^2 S1 S2 S3 S4 + b^4 c^2 S1 S2 S3 S4 + a^2 c^4 S1 S2 S3 S4 + b^2 c^4 S1 S2 S3 S4 + \\
&a^2 b^2 d^2 S1 S2 S3 S4 + b^4 d^2 S1 S2 S3 S4 + a^2 c^2 d^2 S1 S2 S3 S4 + b^2 c^2 d^2 S1 S2 S3 S4))
\end{aligned}
$$

```
-((16 a^2 b^2 c^2 E0 S0 S1 S2 S3) /
  (a^2 b^2 c^2 S0 S1 S2 S3 + b^4 c^2 S0 S1 S2 S3 + a^2 c^4 S0 S1 S2 S3 + b^2 c^4 S0 S1 S2 S3 +
    a^2 b^2 d^2 S0 S1 S2 S3 + b^4 d^2 S0 S1 S2 S3 + a^2 c^2 d^2 S0 S1 S2 S3 + b^2 c^2 d^2 S0 S1 S2 S3 +
    a^2 b^2 c^2 S1^2 S2 S3 - b^4 c^2 S1^2 S2 S3 + a^2 c^4 S1^2 S2 S3 - b^2 c^4 S1^2 S2 S3 + a^2 b^2 d^2 S1^2 S2 S3 -
    b^4 d^2 S1^2 S2 S3 + a^2 c^2 d^2 S1^2 S2 S3 - b^2 c^2 d^2 S1^2 S2 S3 + a^2 b^2 c^2 S0 S2^2 S3 -
    b^4 c^2 S0 S2^2 S3 - a^2 c^4 S0 S2^2 S3 + b^2 c^4 S0 S2^2 S3 + a^2 b^2 d^2 S0 S2^2 S3 - b^4 d^2 S0 S2^2 S3 -
    a^2 c^2 d^2 S0 S2^2 S3 + b^2 c^2 d^2 S0 S2^2 S3 + a^2 b^2 c^2 S1 S2^2 S3 + b^4 c^2 S1 S2^2 S3 -
    a^2 c^4 S1 S2^2 S3 - b^2 c^4 S1 S2^2 S3 + a^2 b^2 d^2 S1 S2^2 S3 + b^4 d^2 S1 S2^2 S3 - a^2 c^2 d^2 S1 S2^2 S3 -
    b^2 c^2 d^2 S1 S2^2 S3 + a^2 b^2 c^2 S0 S1 S3^2 + b^4 c^2 S0 S1 S3^2 - a^2 c^4 S0 S1 S3^2 - b^2 c^4 S0 S1 S3^2 -
    a^2 b^2 d^2 S0 S1 S3^2 - b^4 d^2 S0 S1 S3^2 + a^2 c^2 d^2 S0 S1 S3^2 + b^2 c^2 d^2 S0 S1 S3^2 +
    a^2 b^2 c^2 S1^2 S3^2 - b^4 c^2 S1^2 S3^2 - a^2 c^4 S1^2 S3^2 + b^2 c^4 S1^2 S3^2 - a^2 b^2 d^2 S1^2 S3^2 +
    b^4 d^2 S1^2 S3^2 + a^2 c^2 d^2 S1^2 S3^2 - b^2 c^2 d^2 S1^2 S3^2 + a^2 b^2 c^2 S0 S2 S3^2 - b^4 c^2 S0 S2 S3^2 +
    a^2 c^4 S0 S2 S3^2 - b^2 c^4 S0 S2 S3^2 - a^2 b^2 d^2 S0 S2 S3^2 + b^4 d^2 S0 S2 S3^2 - a^2 c^2 d^2 S0 S2 S3^2 +
    b^2 c^2 d^2 S0 S2 S3^2 + a^2 b^2 c^2 S1 S2 S3^2 + b^4 c^2 S1 S2 S3^2 + a^2 c^4 S1 S2 S3^2 + b^2 c^4 S1 S2 S3^2 -
    a^2 b^2 d^2 S1 S2 S3^2 - b^4 d^2 S1 S2 S3^2 - a^2 c^2 d^2 S1 S2 S3^2 - b^2 c^2 d^2 S1 S2 S3^2 +
    a^2 b^2 c^2 S0 S1 S2 S4 + b^4 c^2 S0 S1 S2 S4 + a^2 c^4 S0 S1 S2 S4 + b^2 c^4 S0 S1 S2 S4 -
    a^2 b^2 d^2 S0 S1 S2 S4 - b^4 d^2 S0 S1 S2 S4 - a^2 c^2 d^2 S0 S1 S2 S4 - b^2 c^2 d^2 S0 S1 S2 S4 +
    a^2 b^2 c^2 S1^2 S2 S4 - b^4 c^2 S1^2 S2 S4 + a^2 c^4 S1^2 S2 S4 - b^2 c^4 S1^2 S2 S4 - a^2 b^2 d^2 S1^2 S2 S4 +
    b^4 d^2 S1^2 S2 S4 - a^2 c^2 d^2 S1^2 S2 S4 + b^2 c^2 d^2 S1^2 S2 S4 + a^2 b^2 c^2 S0 S2^2 S4 -
    b^4 c^2 S0 S2^2 S4 - a^2 c^4 S0 S2^2 S4 + b^2 c^4 S0 S2^2 S4 - a^2 b^2 d^2 S0 S2^2 S4 + b^4 d^2 S0 S2^2 S4 +
    a^2 c^2 d^2 S0 S2^2 S4 - b^2 c^2 d^2 S0 S2^2 S4 + a^2 b^2 c^2 S1 S2^2 S4 + b^4 c^2 S1 S2^2 S4 -
    a^2 c^4 S1 S2^2 S4 - b^2 c^4 S1 S2^2 S4 - a^2 b^2 d^2 S1 S2^2 S4 - b^4 d^2 S1 S2^2 S4 + a^2 c^2 d^2 S1 S2^2 S4 +
    b^2 c^2 d^2 S1 S2^2 S4 + a^2 b^2 c^2 S0 S1 S3 S4 + b^4 c^2 S0 S1 S3 S4 - a^2 c^4 S0 S1 S3 S4 -
    b^2 c^4 S0 S1 S3 S4 + a^2 b^2 d^2 S0 S1 S3 S4 + b^4 d^2 S0 S1 S3 S4 - a^2 c^2 d^2 S0 S1 S3 S4 -
    b^2 c^2 d^2 S0 S1 S3 S4 + a^2 b^2 c^2 S1^2 S3 S4 - b^4 c^2 S1^2 S3 S4 - a^2 c^4 S1^2 S3 S4 + b^2 c^4 S1^2 S3 S4 +
    a^2 b^2 d^2 S1^2 S3 S4 - b^4 d^2 S1^2 S3 S4 - a^2 c^2 d^2 S1^2 S3 S4 + b^2 c^2 d^2 S1^2 S3 S4 +
    a^2 b^2 c^2 S0 S2 S3 S4 - b^4 c^2 S0 S2 S3 S4 + a^2 c^4 S0 S2 S3 S4 - b^2 c^4 S0 S2 S3 S4 +
    a^2 b^2 d^2 S0 S2 S3 S4 - b^4 d^2 S0 S2 S3 S4 + a^2 c^2 d^2 S0 S2 S3 S4 - b^2 c^2 d^2 S0 S2 S3 S4 +
    a^2 b^2 c^2 S1 S2 S3 S4 + b^4 c^2 S1 S2 S3 S4 + a^2 c^4 S1 S2 S3 S4 + b^2 c^4 S1 S2 S3 S4 +
    a^2 b^2 d^2 S1 S2 S3 S4 + b^4 d^2 S1 S2 S3 S4 + a^2 c^2 d^2 S1 S2 S3 S4 + b^2 c^2 d^2 S1 S2 S3 S4))
```

FullSimplify[A4]

```
-((16 a^2 b^2 c^2 E0 S0 S1 S2 S3) /
  (b^2 (S0 - S1) (c^2 (S1 + S2) (d^2 (S2 + S3) (S3 - S4) + c^2 (S2 - S3) (S3 + S4)) +
        b^2 (S1 - S2) (d^2 (S2 - S3) (S3 - S4) + c^2 (S2 + S3) (S3 + S4))) +
    a^2 (S0 + S1) (c^2 (S1 - S2) (d^2 (S2 + S3) (S3 - S4) + c^2 (S2 - S3) (S3 + S4)) +
        b^2 (S1 + S2) (d^2 (S2 - S3) (S3 - S4) + c^2 (S2 + S3) (S3 + S4)))))
```

/ Step 2 : Analytical expressions for the voltages in all the model area : 0, 1, 2, 3, 4. * /

V0[r_, θ_] := -E0 r Cos[θ] + C0 / r Cos[θ]

V1[r_, θ_] := r A1 Cos[θ] + C1 / r Cos[θ]

```
V2[r_, θ_] := r A2 Cos[θ] + C2 / r Cos[θ]

V3[r_, θ_] := r A3 Cos[θ] + C3 / r Cos[θ]

V4[r_, θ_] := r A4 Cos[θ]

V0[r, θ]

-E0 r Cos[θ] -
 ((a^4 b^2 c^2 E0 S0 S1 S2 S3 + a^2 b^4 c^2 E0 S0 S1 S2 S3 + a^4 c^4 E0 S0 S1 S2 S3 + a^2 b^2 c^4 E0 S0 S1 S2 S3 +
      a^4 b^2 d^2 E0 S0 S1 S2 S3 + a^2 b^4 d^2 E0 S0 S1 S2 S3 + a^4 c^2 d^2 E0 S0 S1 S2 S3 +
      a^2 b^2 c^2 d^2 E0 S0 S1 S2 S3 - a^4 b^2 c^2 E0 S1^2 S2 S3 + a^2 b^4 c^2 E0 S1^2 S2 S3 - a^4 c^4 E0 S1^2 S2 S3 +
      a^2 b^2 c^4 E0 S1^2 S2 S3 - a^4 b^2 d^2 E0 S1^2 S2 S3 + a^2 b^4 d^2 E0 S1^2 S2 S3 - a^4 c^2 d^2 E0 S1^2 S2 S3 +
      a^2 b^2 c^2 d^2 E0 S1^2 S2 S3 + a^4 b^2 c^2 E0 S0 S2^2 S3 - a^2 b^4 c^2 E0 S0 S2^2 S3 - a^4 c^4 E0 S0 S2^2 S3 +
      a^2 b^2 c^4 E0 S0 S2^2 S3 + a^4 b^2 d^2 E0 S0 S2^2 S3 - a^2 b^4 d^2 E0 S0 S2^2 S3 - a^4 c^2 d^2 E0 S0 S2^2 S3 +
      a^2 b^2 c^2 d^2 E0 S0 S2^2 S3 - a^4 b^2 c^2 E0 S1 S2^2 S3 - a^2 b^4 c^2 E0 S1 S2^2 S3 + a^4 c^4 E0 S1 S2^2 S3 +
      a^2 b^2 c^4 E0 S1 S2^2 S3 - a^4 b^2 d^2 E0 S1 S2^2 S3 - a^2 b^4 d^2 E0 S1 S2^2 S3 + a^4 c^2 d^2 E0 S1 S2^2 S3 +
      a^2 b^2 c^2 d^2 E0 S1 S2^2 S3 + a^4 b^2 c^2 E0 S0 S1 S3^2 + a^2 b^4 c^2 E0 S0 S1 S3^2 - a^4 c^4 E0 S0 S1 S3^2 -
      a^2 b^2 c^4 E0 S0 S1 S3^2 - a^4 b^2 d^2 E0 S0 S1 S3^2 - a^2 b^4 d^2 E0 S0 S1 S3^2 + a^4 c^2 d^2 E0 S0 S1 S3^2 +
      a^2 b^2 c^2 d^2 E0 S0 S1 S3^2 - a^4 b^2 c^2 E0 S1^2 S3^2 + a^2 b^4 c^2 E0 S1^2 S3^2 + a^4 c^4 E0 S1^2 S3^2 -
      a^2 b^2 c^4 E0 S1^2 S3^2 + a^4 b^2 d^2 E0 S1^2 S3^2 - a^2 b^4 d^2 E0 S1^2 S3^2 - a^4 c^2 d^2 E0 S1^2 S3^2 +
      a^2 b^2 c^2 d^2 E0 S1^2 S3^2 + a^4 b^2 c^2 E0 S0 S2 S3^2 - a^2 b^4 c^2 E0 S0 S2 S3^2 + a^4 c^4 E0 S0 S2 S3^2 -
      a^2 b^2 c^4 E0 S0 S2 S3^2 - a^4 b^2 d^2 E0 S0 S2 S3^2 + a^2 b^4 d^2 E0 S0 S2 S3^2 - a^4 c^2 d^2 E0 S0 S2 S3^2 +
      a^2 b^2 c^2 d^2 E0 S0 S2 S3^2 - a^4 b^2 c^2 E0 S1 S2 S3^2 - a^2 b^4 c^2 E0 S1 S2 S3^2 -
      a^4 c^4 E0 S1 S2 S3^2 - a^2 b^2 c^4 E0 S1 S2 S3^2 + a^4 b^2 d^2 E0 S1 S2 S3^2 + a^2 b^4 d^2 E0 S1 S2 S3^2 +
      a^4 c^2 d^2 E0 S1 S2 S3^2 + a^2 b^2 c^2 d^2 E0 S1 S2 S3^2 + a^4 b^2 c^2 E0 S0 S1 S2 S4 +
      a^2 b^4 c^2 E0 S0 S1 S2 S4 + a^4 c^4 E0 S0 S1 S2 S4 + a^2 b^2 c^4 E0 S0 S1 S2 S4 -
      a^4 b^2 d^2 E0 S0 S1 S2 S4 - a^2 b^4 d^2 E0 S0 S1 S2 S4 - a^4 c^2 d^2 E0 S0 S1 S2 S4 -
      a^2 b^2 c^2 d^2 E0 S0 S1 S2 S4 - a^4 b^2 c^2 E0 S1^2 S2 S4 + a^2 b^4 c^2 E0 S1^2 S2 S4 -
      a^4 c^4 E0 S1^2 S2 S4 + a^2 b^2 c^4 E0 S1^2 S2 S4 + a^4 b^2 d^2 E0 S1^2 S2 S4 - a^2 b^4 d^2 E0 S1^2 S2 S4 +
      a^4 c^2 d^2 E0 S1^2 S2 S4 - a^2 b^2 c^2 d^2 E0 S1^2 S2 S4 + a^4 b^2 c^2 E0 S0 S2^2 S4 - a^2 b^4 c^2 E0 S0 S2^2 S4 -
      a^4 c^4 E0 S0 S2^2 S4 + a^2 b^2 c^4 E0 S0 S2^2 S4 - a^4 b^2 d^2 E0 S0 S2^2 S4 + a^2 b^4 d^2 E0 S0 S2^2 S4 +
      a^4 c^2 d^2 E0 S0 S2^2 S4 - a^2 b^2 c^2 d^2 E0 S0 S2^2 S4 - a^4 b^2 c^2 E0 S1 S2^2 S4 - a^2 b^4 c^2 E0 S1 S2^2 S4 +
      a^4 c^4 E0 S1 S2^2 S4 + a^2 b^2 c^4 E0 S1 S2^2 S4 + a^4 b^2 d^2 E0 S1 S2^2 S4 + a^2 b^4 d^2 E0 S1 S2^2 S4 -
      a^4 c^2 d^2 E0 S1 S2^2 S4 - a^2 b^2 c^2 d^2 E0 S1 S2^2 S4 + a^4 b^2 c^2 E0 S0 S1 S3 S4 +
      a^2 b^4 c^2 E0 S0 S1 S3 S4 - a^4 c^4 E0 S0 S1 S3 S4 - a^2 b^2 c^4 E0 S0 S1 S3 S4 +
      a^4 b^2 d^2 E0 S0 S1 S3 S4 + a^2 b^4 d^2 E0 S0 S1 S3 S4 - a^4 c^2 d^2 E0 S0 S1 S3 S4 -
      a^2 b^2 c^2 d^2 E0 S0 S1 S3 S4 - a^4 b^2 c^2 E0 S1^2 S3 S4 + a^2 b^4 c^2 E0 S1^2 S3 S4 +
      a^4 c^4 E0 S1^2 S3 S4 - a^2 b^2 c^4 E0 S1^2 S3 S4 - a^4 b^2 d^2 E0 S1^2 S3 S4 + a^2 b^4 d^2 E0 S1^2 S3 S4 +
      a^4 c^2 d^2 E0 S1^2 S3 S4 - a^2 b^2 c^2 d^2 E0 S1^2 S3 S4 + a^4 b^2 c^2 E0 S0 S2 S3 S4 -
      a^2 b^4 c^2 E0 S0 S2 S3 S4 + a^4 c^4 E0 S0 S2 S3 S4 - a^2 b^2 c^4 E0 S0 S2 S3 S4 +
      a^4 b^2 d^2 E0 S0 S2 S3 S4 - a^2 b^4 d^2 E0 S0 S2 S3 S4 + a^4 c^2 d^2 E0 S0 S2 S3 S4 -
      a^2 b^2 c^2 d^2 E0 S0 S2 S3 S4 - a^4 b^2 c^2 E0 S1 S2 S3 S4 - a^2 b^4 c^2 E0 S1 S2 S3 S4 -
      a^4 c^4 E0 S1 S2 S3 S4 - a^2 b^2 c^4 E0 S1 S2 S3 S4 - a^4 b^2 d^2 E0 S1 S2 S3 S4 -
      a^2 b^4 d^2 E0 S1 S2 S3 S4 - a^4 c^2 d^2 E0 S1 S2 S3 S4 - a^2 b^2 c^2 d^2 E0 S1 S2 S3 S4) Cos[θ]) /
```

$$
\begin{aligned}
&\big(r\,\big(a^2 b^2 c^2\, S0\, S1\, S2\, S3 + b^4 c^2\, S0\, S1\, S2\, S3 + a^2 c^4\, S0\, S1\, S2\, S3 + b^2 c^4\, S0\, S1\, S2\, S3 +\\
&\quad a^2 b^2 d^2\, S0\, S1\, S2\, S3 + b^4 d^2\, S0\, S1\, S2\, S3 + a^2 c^2 d^2\, S0\, S1\, S2\, S3 + b^2 c^2 d^2\, S0\, S1\, S2\, S3 +\\
&\quad a^2 b^2 c^2\, S1^2\, S2\, S3 - b^4 c^2\, S1^2\, S2\, S3 + a^2 c^4\, S1^2\, S2\, S3 - b^2 c^4\, S1^2\, S2\, S3 + a^2 b^2 d^2\, S1^2\, S2\, S3 -\\
&\quad b^4 d^2\, S1^2\, S2\, S3 + a^2 c^2 d^2\, S1^2\, S2\, S3 - b^2 c^2 d^2\, S1^2\, S2\, S3 + a^2 b^2 c^2\, S0\, S2^2\, S3 -\\
&\quad b^4 c^2\, S0\, S2^2\, S3 - a^2 c^4\, S0\, S2^2\, S3 + b^2 c^4\, S0\, S2^2\, S3 + a^2 b^2 d^2\, S0\, S2^2\, S3 - b^4 d^2\, S0\, S2^2\, S3 -\\
&\quad a^2 c^2 d^2\, S0\, S2^2\, S3 + b^2 c^2 d^2\, S0\, S2^2\, S3 + a^2 b^2 c^2\, S1\, S2^2\, S3 + b^4 c^2\, S1\, S2^2\, S3 -\\
&\quad a^2 c^4\, S1\, S2^2\, S3 - b^2 c^4\, S1\, S2^2\, S3 + a^2 b^2 d^2\, S1\, S2^2\, S3 + b^4 d^2\, S1\, S2^2\, S3 - a^2 c^2 d^2\, S1\, S2^2\, S3 -\\
&\quad b^2 c^2 d^2\, S1\, S2^2\, S3 + a^2 b^2 c^2\, S0\, S1\, S3^2 + b^4 c^2\, S0\, S1\, S3^2 - a^2 c^4\, S0\, S1\, S3^2 - b^2 c^4\, S0\, S1\, S3^2 -\\
&\quad a^2 b^2 d^2\, S0\, S1\, S3^2 - b^4 d^2\, S0\, S1\, S3^2 + a^2 c^2 d^2\, S0\, S1\, S3^2 + b^2 c^2 d^2\, S0\, S1\, S3^2 +\\
&\quad a^2 b^2 c^2\, S1^2\, S3^2 - b^4 c^2\, S1^2\, S3^2 - a^2 c^4\, S1^2\, S3^2 + b^2 c^4\, S1^2\, S3^2 - a^2 b^2 d^2\, S1^2\, S3^2 +\\
&\quad b^4 d^2\, S1^2\, S3^2 + a^2 c^2 d^2\, S1^2\, S3^2 - b^2 c^2 d^2\, S1^2\, S3^2 + a^2 b^2 c^2\, S0\, S2\, S3^2 - b^4 c^2\, S0\, S2\, S3^2 +\\
&\quad a^2 c^4\, S0\, S2\, S3^2 - b^2 c^4\, S0\, S2\, S3^2 - a^2 b^2 d^2\, S0\, S2\, S3^2 + b^4 d^2\, S0\, S2\, S3^2 - a^2 c^2 d^2\, S0\, S2\, S3^2 +\\
&\quad b^2 c^2 d^2\, S0\, S2\, S3^2 + a^2 b^2 c^2\, S1\, S2\, S3^2 + b^4 c^2\, S1\, S2\, S3^2 + a^2 c^4\, S1\, S2\, S3^2 + b^2 c^4\, S1\, S2\, S3^2 -\\
&\quad a^2 b^2 d^2\, S1\, S2\, S3^2 - b^4 d^2\, S1\, S2\, S3^2 - a^2 c^2 d^2\, S1\, S2\, S3^2 - b^2 c^2 d^2\, S1\, S2\, S3^2 +\\
&\quad a^2 b^2 c^2\, S0\, S1\, S2\, S4 + b^4 c^2\, S0\, S1\, S2\, S4 + a^2 c^4\, S0\, S1\, S2\, S4 + b^2 c^4\, S0\, S1\, S2\, S4 -\\
&\quad a^2 b^2 d^2\, S0\, S1\, S2\, S4 - b^4 d^2\, S0\, S1\, S2\, S4 - a^2 c^2 d^2\, S0\, S1\, S2\, S4 - b^2 c^2 d^2\, S0\, S1\, S2\, S4 +\\
&\quad a^2 b^2 c^2\, S1^2\, S2\, S4 - b^4 c^2\, S1^2\, S2\, S4 + a^2 c^4\, S1^2\, S2\, S4 - b^2 c^4\, S1^2\, S2\, S4 - a^2 b^2 d^2\, S1^2\, S2\, S4 +\\
&\quad b^4 d^2\, S1^2\, S2\, S4 - a^2 c^2 d^2\, S1^2\, S2\, S4 + b^2 c^2 d^2\, S1^2\, S2\, S4 + a^2 b^2 c^2\, S0\, S2^2\, S4 -\\
&\quad b^4 c^2\, S0\, S2^2\, S4 - a^2 c^4\, S0\, S2^2\, S4 + b^2 c^4\, S0\, S2^2\, S4 - a^2 b^2 d^2\, S0\, S2^2\, S4 + b^4 d^2\, S0\, S2^2\, S4 +\\
&\quad a^2 c^2 d^2\, S0\, S2^2\, S4 - b^2 c^2 d^2\, S0\, S2^2\, S4 + a^2 b^2 c^2\, S1\, S2^2\, S4 + b^4 c^2\, S1\, S2^2\, S4 -\\
&\quad a^2 c^4\, S1\, S2^2\, S4 - b^2 c^4\, S1\, S2^2\, S4 - a^2 b^2 d^2\, S1\, S2^2\, S4 - b^4 d^2\, S1\, S2^2\, S4 + a^2 c^2 d^2\, S1\, S2^2\, S4 +\\
&\quad b^2 c^2 d^2\, S1\, S2^2\, S4 + a^2 b^2 c^2\, S0\, S1\, S3\, S4 + b^4 c^2\, S0\, S1\, S3\, S4 - a^2 c^4\, S0\, S1\, S3\, S4 -\\
&\quad b^2 c^4\, S0\, S1\, S3\, S4 + a^2 b^2 d^2\, S0\, S1\, S3\, S4 + b^4 d^2\, S0\, S1\, S3\, S4 - a^2 c^2 d^2\, S0\, S1\, S3\, S4 -\\
&\quad b^2 c^2 d^2\, S0\, S1\, S3\, S4 + a^2 b^2 c^2\, S1^2\, S3\, S4 - b^4 c^2\, S1^2\, S3\, S4 - a^2 c^4\, S1^2\, S3\, S4 + b^2 c^4\, S1^2\, S3\, S4 +\\
&\quad a^2 b^2 d^2\, S1^2\, S3\, S4 - b^4 d^2\, S1^2\, S3\, S4 - a^2 c^2 d^2\, S1^2\, S3\, S4 + b^2 c^2 d^2\, S1^2\, S3\, S4 +\\
&\quad a^2 b^2 c^2\, S0\, S2\, S3\, S4 - b^4 c^2\, S0\, S2\, S3\, S4 + a^2 c^4\, S0\, S2\, S3\, S4 - b^2 c^4\, S0\, S2\, S3\, S4 +\\
&\quad a^2 b^2 d^2\, S0\, S2\, S3\, S4 - b^4 d^2\, S0\, S2\, S3\, S4 + a^2 c^2 d^2\, S0\, S2\, S3\, S4 - b^2 c^2 d^2\, S0\, S2\, S3\, S4 +\\
&\quad a^2 b^2 c^2\, S1\, S2\, S3\, S4 + b^4 c^2\, S1\, S2\, S3\, S4 + a^2 c^4\, S1\, S2\, S3\, S4 + b^2 c^4\, S1\, S2\, S3\, S4 +\\
&\quad a^2 b^2 d^2\, S1\, S2\, S3\, S4 + b^4 d^2\, S1\, S2\, S3\, S4 + a^2 c^2 d^2\, S1\, S2\, S3\, S4 + b^2 c^2 d^2\, S1\, S2\, S3\, S4\big)\big)
\end{aligned}
$$

FullSimplify[%24]

$$-\Big(\Big(\text{E0}\,\Big(b^2 r^2 (\text{S0}-\text{S1})\,\Big(c^2 (\text{S1}+\text{S2})\,\Big(d^2 (\text{S2}+\text{S3})\,(\text{S3}-\text{S4})+c^2 (\text{S2}-\text{S3})\,(\text{S3}+\text{S4})\Big)+$$
$$b^2 (\text{S1}-\text{S2})\,\Big(d^2 (\text{S2}-\text{S3})\,(\text{S3}-\text{S4})+c^2 (\text{S2}+\text{S3})\,(\text{S3}+\text{S4})\Big)\Big)+$$
$$a^4 (\text{S0}-\text{S1})\,\Big(c^2 (\text{S1}-\text{S2})\,\Big(d^2 (\text{S2}+\text{S3})\,(\text{S3}-\text{S4})+c^2 (\text{S2}-\text{S3})\,(\text{S3}+\text{S4})\Big)+$$
$$b^2 (\text{S1}+\text{S2})\,\Big(d^2 (\text{S2}-\text{S3})\,(\text{S3}-\text{S4})+c^2 (\text{S2}+\text{S3})\,(\text{S3}+\text{S4})\Big)\Big)+$$
$$a^2 (\text{S0}+\text{S1})\,\Big(c^2 r^2 (\text{S1}-\text{S2})\,\Big(d^2 (\text{S2}+\text{S3})\,(\text{S3}-\text{S4})+c^2 (\text{S2}-\text{S3})\,(\text{S3}+\text{S4})\Big)+$$
$$b^4 (\text{S1}-\text{S2})\,\Big(d^2 (\text{S2}-\text{S3})\,(\text{S3}-\text{S4})+c^2 (\text{S2}+\text{S3})\,(\text{S3}+\text{S4})\Big)+$$
$$b^2 (\text{S1}+\text{S2})\,\Big(d^2 r^2 (\text{S2}-\text{S3})\,(\text{S3}-\text{S4})+c^4 (\text{S2}-\text{S3})\,(\text{S3}+\text{S4})+$$
$$c^2 (\text{S2}+\text{S3})\,\Big(d^2 (\text{S3}-\text{S4})+r^2 (\text{S3}+\text{S4})\Big)\Big)\Big)\Big)\,\text{Cos}[\theta]\Big)\Big/$$
$$\Big(r\,\Big(b^2 (\text{S0}-\text{S1})\,\Big(c^2 (\text{S1}+\text{S2})\,\Big(d^2 (\text{S2}+\text{S3})\,(\text{S3}-\text{S4})+c^2 (\text{S2}-\text{S3})\,(\text{S3}+\text{S4})\Big)+$$
$$b^2 (\text{S1}-\text{S2})\,\Big(d^2 (\text{S2}-\text{S3})\,(\text{S3}-\text{S4})+c^2 (\text{S2}+\text{S3})\,(\text{S3}+\text{S4})\Big)\Big)+$$
$$a^2 (\text{S0}+\text{S1})\,\Big(c^2 (\text{S1}-\text{S2})\,\Big(d^2 (\text{S2}+\text{S3})\,(\text{S3}-\text{S4})+c^2 (\text{S2}-\text{S3})\,(\text{S3}+\text{S4})\Big)+$$
$$b^2 (\text{S1}+\text{S2})\,\Big(d^2 (\text{S2}-\text{S3})\,(\text{S3}-\text{S4})+c^2 (\text{S2}+\text{S3})\,(\text{S3}+\text{S4})\Big)\Big)\Big)\Big)$$

V1[r, θ]

$$-\Big(\Big(2\,\text{E0}\,\text{S0}\,\Big(a^2 b^4 c^2 \text{S1}\,\text{S2}\,\text{S3}+a^2 b^2 c^4 \text{S1}\,\text{S2}\,\text{S3}+a^2 b^4 d^2 \text{S1}\,\text{S2}\,\text{S3}+a^2 b^2 c^2 d^2 \text{S1}\,\text{S2}\,\text{S3}-$$
$$a^2 b^4 c^2 \text{S2}^2 \text{S3}+a^2 b^2 c^4 \text{S2}^2 \text{S3}-a^2 b^4 d^2 \text{S2}^2 \text{S3}+a^2 b^2 c^2 d^2 \text{S2}^2 \text{S3}+a^2 b^4 c^2 \text{S1}\,\text{S3}^2-$$
$$a^2 b^2 c^4 \text{S1}\,\text{S3}^2-a^2 b^4 d^2 \text{S1}\,\text{S3}^2+a^2 b^2 c^2 d^2 \text{S1}\,\text{S3}^2-a^2 b^4 c^2 \text{S2}\,\text{S3}^2-$$
$$a^2 b^2 c^4 \text{S2}\,\text{S3}^2+a^2 b^4 d^2 \text{S2}\,\text{S3}^2+a^2 b^2 c^2 d^2 \text{S2}\,\text{S3}^2+a^2 b^4 c^2 \text{S1}\,\text{S2}\,\text{S4}+$$
$$a^2 b^2 c^4 \text{S1}\,\text{S2}\,\text{S4}-a^2 b^4 d^2 \text{S1}\,\text{S2}\,\text{S4}-a^2 b^2 c^2 d^2 \text{S1}\,\text{S2}\,\text{S4}-a^2 b^4 c^2 \text{S2}^2 \text{S4}+$$
$$a^2 b^2 c^4 \text{S2}^2 \text{S4}+a^2 b^4 d^2 \text{S2}^2 \text{S4}-a^2 b^2 c^2 d^2 \text{S2}^2 \text{S4}+a^2 b^4 c^2 \text{S1}\,\text{S3}\,\text{S4}-$$
$$a^2 b^2 c^4 \text{S1}\,\text{S3}\,\text{S4}+a^2 b^4 d^2 \text{S1}\,\text{S3}\,\text{S4}-a^2 b^2 c^2 d^2 \text{S1}\,\text{S3}\,\text{S4}-a^2 b^4 c^2 \text{S2}\,\text{S3}\,\text{S4}-$$
$$a^2 b^2 c^4 \text{S2}\,\text{S3}\,\text{S4}-a^2 b^4 d^2 \text{S2}\,\text{S3}\,\text{S4}-a^2 b^2 c^2 d^2 \text{S2}\,\text{S3}\,\text{S4}\Big)\,\text{Cos}[\theta]\Big)\Big/$$
$$\Big(r\,\Big(a^2 b^2 c^2 \text{S0}\,\text{S1}\,\text{S2}\,\text{S3}+b^4 c^2 \text{S0}\,\text{S1}\,\text{S2}\,\text{S3}+a^2 c^4 \text{S0}\,\text{S1}\,\text{S2}\,\text{S3}+b^2 c^4 \text{S0}\,\text{S1}\,\text{S2}\,\text{S3}+$$
$$a^2 b^2 d^2 \text{S0}\,\text{S1}\,\text{S2}\,\text{S3}+b^4 d^2 \text{S0}\,\text{S1}\,\text{S2}\,\text{S3}+a^2 c^2 d^2 \text{S0}\,\text{S1}\,\text{S2}\,\text{S3}+b^2 c^2 d^2 \text{S0}\,\text{S1}\,\text{S2}\,\text{S3}+$$
$$a^2 b^2 c^2 \text{S1}^2 \text{S2}\,\text{S3}-b^4 c^2 \text{S1}^2 \text{S2}\,\text{S3}+a^2 c^4 \text{S1}^2 \text{S2}\,\text{S3}-b^2 c^4 \text{S1}^2 \text{S2}\,\text{S3}+a^2 b^2 d^2 \text{S1}^2 \text{S2}\,\text{S3}-$$
$$b^4 d^2 \text{S1}^2 \text{S2}\,\text{S3}+a^2 c^2 d^2 \text{S1}^2 \text{S2}\,\text{S3}-b^2 c^2 d^2 \text{S1}^2 \text{S2}\,\text{S3}+a^2 b^2 c^2 \text{S0}\,\text{S2}^2 \text{S3}-$$
$$b^4 c^2 \text{S0}\,\text{S2}^2 \text{S3}-a^2 c^4 \text{S0}\,\text{S2}^2 \text{S3}+b^2 c^4 \text{S0}\,\text{S2}^2 \text{S3}+a^2 b^2 d^2 \text{S0}\,\text{S2}^2 \text{S3}-b^4 d^2 \text{S0}\,\text{S2}^2 \text{S3}-$$
$$a^2 c^2 d^2 \text{S0}\,\text{S2}^2 \text{S3}+b^2 c^2 d^2 \text{S0}\,\text{S2}^2 \text{S3}+a^2 b^2 c^2 \text{S1}\,\text{S2}^2 \text{S3}+b^4 c^2 \text{S1}\,\text{S2}^2 \text{S3}-$$
$$a^2 c^4 \text{S1}\,\text{S2}^2 \text{S3}-b^2 c^4 \text{S1}\,\text{S2}^2 \text{S3}+a^2 b^2 d^2 \text{S1}\,\text{S2}^2 \text{S3}+b^4 d^2 \text{S1}\,\text{S2}^2 \text{S3}-a^2 c^2 d^2 \text{S1}\,\text{S2}^2 \text{S3}-$$
$$b^2 c^2 d^2 \text{S1}\,\text{S2}^2 \text{S3}+a^2 b^2 c^2 \text{S0}\,\text{S1}\,\text{S3}^2+b^4 c^2 \text{S0}\,\text{S1}\,\text{S3}^2-a^2 c^4 \text{S0}\,\text{S1}\,\text{S3}^2-$$
$$b^2 c^4 \text{S0}\,\text{S1}\,\text{S3}^2-a^2 b^2 d^2 \text{S0}\,\text{S1}\,\text{S3}^2-b^4 d^2 \text{S0}\,\text{S1}\,\text{S3}^2+a^2 c^2 d^2 \text{S0}\,\text{S1}\,\text{S3}^2+$$
$$b^2 c^2 d^2 \text{S0}\,\text{S1}\,\text{S3}^2+a^2 b^2 c^2 \text{S1}^2 \text{S3}^2-b^4 c^2 \text{S1}^2 \text{S3}^2-a^2 c^4 \text{S1}^2 \text{S3}^2+b^2 c^4 \text{S1}^2 \text{S3}^2-$$
$$a^2 b^2 d^2 \text{S1}^2 \text{S3}^2+b^4 d^2 \text{S1}^2 \text{S3}^2+a^2 c^2 d^2 \text{S1}^2 \text{S3}^2-b^2 c^2 d^2 \text{S1}^2 \text{S3}^2+a^2 b^2 c^2 \text{S0}\,\text{S2}\,\text{S3}^2-$$
$$b^4 c^2 \text{S0}\,\text{S2}\,\text{S3}^2+a^2 c^4 \text{S0}\,\text{S2}\,\text{S3}^2-b^2 c^4 \text{S0}\,\text{S2}\,\text{S3}^2-a^2 b^2 d^2 \text{S0}\,\text{S2}\,\text{S3}^2+b^4 d^2 \text{S0}\,\text{S2}\,\text{S3}^2-$$
$$a^2 c^2 d^2 \text{S0}\,\text{S2}\,\text{S3}^2+b^2 c^2 d^2 \text{S0}\,\text{S2}\,\text{S3}^2+a^2 b^2 c^2 \text{S1}\,\text{S2}\,\text{S3}^2+b^4 c^2 \text{S1}\,\text{S2}\,\text{S3}^2+$$
$$a^2 c^4 \text{S1}\,\text{S2}\,\text{S3}^2+b^2 c^4 \text{S1}\,\text{S2}\,\text{S3}^2-a^2 b^2 d^2 \text{S1}\,\text{S2}\,\text{S3}^2-b^4 d^2 \text{S1}\,\text{S2}\,\text{S3}^2-a^2 c^2 d^2 \text{S1}\,\text{S2}\,\text{S3}^2-$$
$$b^2 c^2 d^2 \text{S1}\,\text{S2}\,\text{S3}^2+a^2 b^2 c^2 \text{S0}\,\text{S1}\,\text{S2}\,\text{S4}+b^4 c^2 \text{S0}\,\text{S1}\,\text{S2}\,\text{S4}+a^2 c^4 \text{S0}\,\text{S1}\,\text{S2}\,\text{S4}+$$
$$b^2 c^4 \text{S0}\,\text{S1}\,\text{S2}\,\text{S4}-a^2 b^2 d^2 \text{S0}\,\text{S1}\,\text{S2}\,\text{S4}-b^4 d^2 \text{S0}\,\text{S1}\,\text{S2}\,\text{S4}-a^2 c^2 d^2 \text{S0}\,\text{S1}\,\text{S2}\,\text{S4}-$$
$$b^2 c^2 d^2 \text{S0}\,\text{S1}\,\text{S2}\,\text{S4}+a^2 b^2 c^2 \text{S1}^2 \text{S2}\,\text{S4}-b^4 c^2 \text{S1}^2 \text{S2}\,\text{S4}+a^2 c^4 \text{S1}^2 \text{S2}\,\text{S4}-$$
$$b^2 c^4 \text{S1}^2 \text{S2}\,\text{S4}-a^2 b^2 d^2 \text{S1}^2 \text{S2}\,\text{S4}+b^4 d^2 \text{S1}^2 \text{S2}\,\text{S4}-a^2 c^2 d^2 \text{S1}^2 \text{S2}\,\text{S4}+$$
$$b^2 c^2 d^2 \text{S1}^2 \text{S2}\,\text{S4}+a^2 b^2 c^2 \text{S0}\,\text{S2}^2 \text{S4}-b^4 c^2 \text{S0}\,\text{S2}^2 \text{S4}-a^2 c^4 \text{S0}\,\text{S2}^2 \text{S4}+$$
$$b^2 c^4 \text{S0}\,\text{S2}^2 \text{S4}-a^2 b^2 d^2 \text{S0}\,\text{S2}^2 \text{S4}+b^4 d^2 \text{S0}\,\text{S2}^2 \text{S4}+a^2 c^2 d^2 \text{S0}\,\text{S2}^2 \text{S4}-$$
$$b^2 c^2 d^2 \text{S0}\,\text{S2}^2 \text{S4}+a^2 b^2 c^2 \text{S1}\,\text{S2}^2 \text{S4}+b^4 c^2 \text{S1}\,\text{S2}^2 \text{S4}-a^2 c^4 \text{S1}\,\text{S2}^2 \text{S4}-b^2 c^4 \text{S1}\,\text{S2}^2 \text{S4}-$$

$$a^2 b^2 d^2 S1\, S2^2\, S4 - b^4 d^2 S1\, S2^2\, S4 + a^2 c^2 d^2 S1\, S2^2\, S4 + b^2 c^2 d^2 S1\, S2^2\, S4 +$$
$$a^2 b^2 c^2 S0\, S1\, S3\, S4 + b^4 c^2 S0\, S1\, S3\, S4 - a^2 c^4 S0\, S1\, S3\, S4 - b^2 c^4 S0\, S1\, S3\, S4 +$$
$$a^2 b^2 d^2 S0\, S1\, S3\, S4 + b^4 d^2 S0\, S1\, S3\, S4 - a^2 c^2 d^2 S0\, S1\, S3\, S4 - b^2 c^2 d^2 S0\, S1\, S3\, S4 +$$
$$a^2 b^2 c^2 S1^2\, S3\, S4 - b^4 c^2 S1^2\, S3\, S4 - a^2 c^4 S1^2\, S3\, S4 + b^2 c^4 S1^2\, S3\, S4 +$$
$$a^2 b^2 d^2 S1^2\, S3\, S4 - b^4 d^2 S1^2\, S3\, S4 - a^2 c^2 d^2 S1^2\, S3\, S4 + b^2 c^2 d^2 S1^2\, S3\, S4 +$$
$$a^2 b^2 c^2 S0\, S2\, S3\, S4 - b^4 c^2 S0\, S2\, S3\, S4 + a^2 c^4 S0\, S2\, S3\, S4 - b^2 c^4 S0\, S2\, S3\, S4 +$$
$$a^2 b^2 d^2 S0\, S2\, S3\, S4 - b^4 d^2 S0\, S2\, S3\, S4 + a^2 c^2 d^2 S0\, S2\, S3\, S4 - b^2 c^2 d^2 S0\, S2\, S3\, S4 +$$
$$a^2 b^2 c^2 S1\, S2\, S3\, S4 + b^4 c^2 S1\, S2\, S3\, S4 + a^2 c^4 S1\, S2\, S3\, S4 + b^2 c^4 S1\, S2\, S3\, S4 +$$
$$a^2 b^2 d^2 S1\, S2\, S3\, S4 + b^4 d^2 S1\, S2\, S3\, S4 + a^2 c^2 d^2 S1\, S2\, S3\, S4 + b^2 c^2 d^2 S1\, S2\, S3\, S4))) -$$

$$\Bigg(2\, E0\, r\, S0 \Bigg(-\frac{1}{b^3} 2\, S1\, S2 \Bigg(-\frac{2\, S2\, S3 \left(\frac{S3}{d}+\frac{S4}{d}\right)}{c^3} - \left(-\frac{S2}{c^3}+\frac{S3}{c^3}\right)\left(S3\left(\frac{S3}{d}+\frac{S4}{d}\right)+\frac{S3\,(-d\, S3 + d\, S4)}{c^2}\right)\Bigg) -$$
$$\left(-\frac{S1}{b^3}+\frac{S2}{b^3}\right)\Bigg(S2\Bigg(-\frac{2\, S2\, S3 \left(\frac{S3}{d}+\frac{S4}{d}\right)}{c^3} - \left(-\frac{S2}{c^3}+\frac{S3}{c^3}\right)\left(S3\left(\frac{S3}{d}+\frac{S4}{d}\right)+\frac{S3\,(-d\, S3 + d\, S4)}{c^2}\right)\Bigg) +$$
$$\frac{1}{b^2} S2 \Bigg(-\frac{2\, S3 \left(-\frac{S2\, S3}{d}-\frac{S2\, S4}{d}\right)}{c} - \left(\frac{S2}{c}+\frac{S3}{c}\right)\left(S3\left(\frac{S3}{d}+\frac{S4}{d}\right)+\frac{S3\,(-d\, S3 + d\, S4)}{c^2}\right)\Bigg)\Bigg)\Bigg)\Bigg)$$

$$\mathrm{Cos}[\theta]\Bigg) \Bigg/ \Bigg(a\Bigg(\left(\frac{S0}{a}+\frac{S1}{a}\right)\Bigg(-\frac{1}{b^3} 2\, S1\, S2 \Bigg(-\frac{2\, S2\, S3 \left(\frac{S3}{d}+\frac{S4}{d}\right)}{c^3} -$$
$$\left(-\frac{S2}{c^3}+\frac{S3}{c^3}\right)\left(S3\left(\frac{S3}{d}+\frac{S4}{d}\right)+\frac{S3\,(-d\, S3 + d\, S4)}{c^2}\right)\Bigg) - \left(-\frac{S1}{b^3}+\frac{S2}{b^3}\right)$$
$$\Bigg(S2\Bigg(-\frac{2\, S2\, S3 \left(\frac{S3}{d}+\frac{S4}{d}\right)}{c^3} - \left(-\frac{S2}{c^3}+\frac{S3}{c^3}\right)\left(S3\left(\frac{S3}{d}+\frac{S4}{d}\right)+\frac{S3\,(-d\, S3 + d\, S4)}{c^2}\right)\Bigg) +$$
$$\frac{1}{b^2} S2 \Bigg(-\frac{2\, S3 \left(-\frac{S2\, S3}{d}-\frac{S2\, S4}{d}\right)}{c} - \left(\frac{S2}{c}+\frac{S3}{c}\right)\left(S3\left(\frac{S3}{d}+\frac{S4}{d}\right)+\frac{S3\,(-d\, S3 + d\, S4)}{c^2}\right)\Bigg)\Bigg)\Bigg) +$$
$$\left(-\frac{S0}{a^3}+\frac{S1}{a^3}\right)\Bigg(\frac{1}{b} 2\, S1\, S2 \Bigg(-\frac{2\, S2\, S3 \left(\frac{S3}{d}+\frac{S4}{d}\right)}{c^3} - \left(-\frac{S2}{c^3}+\frac{S3}{c^3}\right)$$
$$\left(S3\left(\frac{S3}{d}+\frac{S4}{d}\right)+\frac{S3\,(-d\, S3 + d\, S4)}{c^2}\right)\Bigg) - \left(\frac{S1}{b}+\frac{S2}{b}\right)$$
$$\Bigg(S2\Bigg(-\frac{2\, S2\, S3 \left(\frac{S3}{d}+\frac{S4}{d}\right)}{c^3} - \left(-\frac{S2}{c^3}+\frac{S3}{c^3}\right)\left(S3\left(\frac{S3}{d}+\frac{S4}{d}\right)+\frac{S3\,(-d\, S3 + d\, S4)}{c^2}\right)\Bigg) + \frac{1}{b^2}$$
$$S2 \Bigg(-\frac{2\, S3 \left(-\frac{S2\, S3}{d}-\frac{S2\, S4}{d}\right)}{c} - \left(\frac{S2}{c}+\frac{S3}{c}\right)\left(S3\left(\frac{S3}{d}+\frac{S4}{d}\right)+\frac{S3\,(-d\, S3 + d\, S4)}{c^2}\right)\Bigg)\Bigg)\Bigg)\Bigg)\Bigg)\Bigg)$$

FullSimplify[%26]

$$-\Big(\Big(2 a^2 E0\, S0 \left(c^2 r^2 (S1 - S2) \left(d^2 (S2 + S3) (S3 - S4) + c^2 (S2 - S3) (S3 + S4)\right) + \right.$$
$$b^4 (S1 - S2) \left(d^2 (S2 - S3) (S3 - S4) + c^2 (S2 + S3) (S3 + S4)\right) +$$
$$b^2 (S1 + S2) \left(d^2 r^2 (S2 - S3) (S3 - S4) + c^4 (S2 - S3) (S3 + S4) + \right.$$
$$\left.\left. c^2 (S2 + S3) \left(d^2 (S3 - S4) + r^2 (S3 + S4)\right)\right)\right) \mathrm{Cos}[\theta]\Big) \Big/$$
$$\Big(r \Big(b^2 (S0 - S1) \Big(c^2 (S1 + S2) \left(d^2 (S2 + S3) (S3 - S4) + c^2 (S2 - S3) (S3 + S4)\right) +$$
$$b^2 (S1 - S2) \left(d^2 (S2 - S3) (S3 - S4) + c^2 (S2 + S3) (S3 + S4)\right)\Big) +$$
$$a^2 (S0 + S1) \Big(c^2 (S1 - S2) \left(d^2 (S2 + S3) (S3 - S4) + c^2 (S2 - S3) (S3 + S4)\right) +$$
$$b^2 (S1 + S2) \left(d^2 (S2 - S3) (S3 - S4) + c^2 (S2 + S3) (S3 + S4)\right)\Big)\Big)\Big)\Big)$$

V2[r, θ]

$$-\Big(\Big(4 b^2 E0\, r\, S0 \left(a^2 c^2 S1 S2 S3 + a^2 d^2 S1 S2 S3 + a^2 c^2 S1 S3^2 - a^2 d^2 S1 S3^2 + \right.$$
$$\left. a^2 c^2 S1 S2 S4 - a^2 d^2 S1 S2 S4 + a^2 c^2 S1 S3 S4 + a^2 d^2 S1 S3 S4\right) \mathrm{Cos}[\theta]\Big) \Big/$$
$$\Big(a^2 b^2 c^2 S0 S1 S2 S3 + b^4 c^2 S0 S1 S2 S3 + a^2 c^4 S0 S1 S2 S3 + b^2 c^4 S0 S1 S2 S3 +$$
$$a^2 b^2 d^2 S0 S1 S2 S3 + b^4 d^2 S0 S1 S2 S3 + a^2 c^2 d^2 S0 S1 S2 S3 + b^2 c^2 d^2 S0 S1 S2 S3 +$$
$$a^2 b^2 c^2 S1^2 S2 S3 - b^4 c^2 S1^2 S2 S3 + a^2 c^4 S1^2 S2 S3 - b^2 c^4 S1^2 S2 S3 + a^2 b^2 d^2 S1^2 S2 S3 -$$
$$b^4 d^2 S1^2 S2 S3 + a^2 c^2 d^2 S1^2 S2 S3 - b^2 c^2 d^2 S1^2 S2 S3 + a^2 b^2 c^2 S0 S2^2 S3 -$$
$$b^4 c^2 S0 S2^2 S3 - a^2 c^4 S0 S2^2 S3 + b^2 c^4 S0 S2^2 S3 + a^2 b^2 d^2 S0 S2^2 S3 - b^4 d^2 S0 S2^2 S3 -$$
$$a^2 c^2 d^2 S0 S2^2 S3 + b^2 c^2 d^2 S0 S2^2 S3 + a^2 b^2 c^2 S1 S2^2 S3 + b^4 c^2 S1 S2^2 S3 -$$
$$a^2 c^4 S1 S2^2 S3 - b^2 c^4 S1 S2^2 S3 + a^2 b^2 d^2 S1 S2^2 S3 + b^4 d^2 S1 S2^2 S3 - a^2 c^2 d^2 S1 S2^2 S3 -$$
$$b^2 c^2 d^2 S1 S2^2 S3 + a^2 b^2 c^2 S0 S1 S3^2 + b^4 c^2 S0 S1 S3^2 - a^2 c^4 S0 S1 S3^2 - b^2 c^4 S0 S1 S3^2 -$$
$$a^2 b^2 d^2 S0 S1 S3^2 - b^4 d^2 S0 S1 S3^2 + a^2 c^2 d^2 S0 S1 S3^2 + b^2 c^2 d^2 S0 S1 S3^2 +$$
$$a^2 b^2 c^2 S1^2 S3^2 - b^4 c^2 S1^2 S3^2 - a^2 c^4 S1^2 S3^2 + b^2 c^4 S1^2 S3^2 - a^2 b^2 d^2 S1^2 S3^2 +$$
$$b^4 d^2 S1^2 S3^2 + a^2 c^2 d^2 S1^2 S3^2 - b^2 c^2 d^2 S1^2 S3^2 + a^2 b^2 c^2 S0 S2 S3^2 - b^4 c^2 S0 S2 S3^2 +$$
$$a^2 c^4 S0 S2 S3^2 - b^2 c^4 S0 S2 S3^2 - a^2 b^2 d^2 S0 S2 S3^2 + b^4 d^2 S0 S2 S3^2 - a^2 c^2 d^2 S0 S2 S3^2 +$$
$$b^2 c^2 d^2 S0 S2 S3^2 + a^2 b^2 c^2 S1 S2 S3^2 + b^4 c^2 S1 S2 S3^2 + a^2 c^4 S1 S2 S3^2 + b^2 c^4 S1 S2 S3^2 -$$
$$a^2 b^2 d^2 S1 S2 S3^2 - b^4 d^2 S1 S2 S3^2 - a^2 c^2 d^2 S1 S2 S3^2 - b^2 c^2 d^2 S1 S2 S3^2 +$$
$$a^2 b^2 c^2 S0 S1 S2 S4 + b^4 c^2 S0 S1 S2 S4 + a^2 c^4 S0 S1 S2 S4 + b^2 c^4 S0 S1 S2 S4 -$$
$$a^2 b^2 d^2 S0 S1 S2 S4 - b^4 d^2 S0 S1 S2 S4 - a^2 c^2 d^2 S0 S1 S2 S4 - b^2 c^2 d^2 S0 S1 S2 S4 +$$
$$a^2 b^2 c^2 S1^2 S2 S4 - b^4 c^2 S1^2 S2 S4 + a^2 c^4 S1^2 S2 S4 - b^2 c^4 S1^2 S2 S4 - a^2 b^2 d^2 S1^2 S2 S4 +$$
$$b^4 d^2 S1^2 S2 S4 - a^2 c^2 d^2 S1^2 S2 S4 + b^2 c^2 d^2 S1^2 S2 S4 + a^2 b^2 c^2 S0 S2^2 S4 -$$
$$b^4 c^2 S0 S2^2 S4 - a^2 c^4 S0 S2^2 S4 + b^2 c^4 S0 S2^2 S4 - a^2 b^2 d^2 S0 S2^2 S4 + b^4 d^2 S0 S2^2 S4 +$$
$$a^2 c^2 d^2 S0 S2^2 S4 - b^2 c^2 d^2 S0 S2^2 S4 + a^2 b^2 c^2 S1 S2^2 S4 + b^4 c^2 S1 S2^2 S4 -$$
$$a^2 c^4 S1 S2^2 S4 - b^2 c^4 S1 S2^2 S4 - a^2 b^2 d^2 S1 S2^2 S4 - b^4 d^2 S1 S2^2 S4 + a^2 c^2 d^2 S1 S2^2 S4 +$$
$$b^2 c^2 d^2 S1 S2^2 S4 + a^2 b^2 c^2 S0 S1 S3 S4 + b^4 c^2 S0 S1 S3 S4 - a^2 c^4 S0 S1 S3 S4 -$$
$$b^2 c^4 S0 S1 S3 S4 + a^2 b^2 d^2 S0 S1 S3 S4 + b^4 d^2 S0 S1 S3 S4 - a^2 c^2 d^2 S0 S1 S3 S4 -$$
$$b^2 c^2 d^2 S0 S1 S3 S4 + a^2 b^2 c^2 S1^2 S3 S4 - b^4 c^2 S1^2 S3 S4 - a^2 c^4 S1^2 S3 S4 + b^2 c^4 S1^2 S3 S4 +$$
$$a^2 b^2 d^2 S1^2 S3 S4 - b^4 d^2 S1^2 S3 S4 - a^2 c^2 d^2 S1^2 S3 S4 + b^2 c^2 d^2 S1^2 S3 S4 +$$
$$a^2 b^2 c^2 S0 S2 S3 S4 - b^4 c^2 S0 S2 S3 S4 + a^2 c^4 S0 S2 S3 S4 - b^2 c^4 S0 S2 S3 S4 +$$
$$a^2 b^2 d^2 S0 S2 S3 S4 - b^4 d^2 S0 S2 S3 S4 + a^2 c^2 d^2 S0 S2 S3 S4 - b^2 c^2 d^2 S0 S2 S3 S4 +$$
$$a^2 b^2 c^2 S1 S2 S3 S4 + b^4 c^2 S1 S2 S3 S4 + a^2 c^4 S1 S2 S3 S4 + b^2 c^4 S1 S2 S3 S4 +$$
$$a^2 b^2 d^2 S1 S2 S3 S4 + b^4 d^2 S1 S2 S3 S4 + a^2 c^2 d^2 S1 S2 S3 S4 + b^2 c^2 d^2 S1 S2 S3 S4\Big)\Big) -$$
$$\Big(4 E0\, S0 \left(a^2 b^2 c^4 S1 S2 S3 + a^2 b^2 c^2 d^2 S1 S2 S3 - a^2 b^2 c^4 S1 S3^2 + a^2 b^2 c^2 d^2 S1 S3^2 + \right.$$
$$\left. a^2 b^2 c^4 S1 S2 S4 - a^2 b^2 c^2 d^2 S1 S2 S4 - a^2 b^2 c^4 S1 S3 S4 - a^2 b^2 c^2 d^2 S1 S3 S4\right) \mathrm{Cos}[\theta]\Big) \Big/$$

```
(r (a^2 b^2 c^2 S0 S1 S2 S3 + b^4 c^2 S0 S1 S2 S3 + a^2 c^4 S0 S1 S2 S3 + b^2 c^4 S0 S1 S2 S3 +
    a^2 b^2 d^2 S0 S1 S2 S3 + b^4 d^2 S0 S1 S2 S3 + a^2 c^2 d^2 S0 S1 S2 S3 + b^2 c^2 d^2 S0 S1 S2 S3 +
    a^2 b^2 c^2 S1^2 S2 S3 - b^4 c^2 S1^2 S2 S3 + a^2 c^4 S1^2 S2 S3 - b^2 c^4 S1^2 S2 S3 + a^2 b^2 d^2 S1^2 S2 S3 -
    b^4 d^2 S1^2 S2 S3 + a^2 c^2 d^2 S1^2 S2 S3 - b^2 c^2 d^2 S1^2 S2 S3 + a^2 b^2 c^2 S0 S2^2 S3 -
    b^4 c^2 S0 S2^2 S3 - a^2 c^4 S0 S2^2 S3 + b^2 c^4 S0 S2^2 S3 + a^2 b^2 d^2 S0 S2^2 S3 - b^4 d^2 S0 S2^2 S3 -
    a^2 c^2 d^2 S0 S2^2 S3 + b^2 c^2 d^2 S0 S2^2 S3 + a^2 b^2 c^2 S1 S2^2 S3 + b^4 c^2 S1 S2^2 S3 -
    a^2 c^4 S1 S2^2 S3 - b^2 c^4 S1 S2^2 S3 + a^2 b^2 d^2 S1 S2^2 S3 + b^4 d^2 S1 S2^2 S3 - a^2 c^2 d^2 S1 S2^2 S3 -
    b^2 c^2 d^2 S1 S2^2 S3 + a^2 b^2 c^2 S0 S1 S3^2 + b^4 c^2 S0 S1 S3^2 - a^2 c^4 S0 S1 S3^2 - b^2 c^4 S0 S1 S3^2 -
    a^2 b^2 d^2 S0 S1 S3^2 - b^4 d^2 S0 S1 S3^2 + a^2 c^2 d^2 S0 S1 S3^2 + b^2 c^2 d^2 S0 S1 S3^2 +
    a^2 b^2 c^2 S1^2 S3^2 - b^4 c^2 S1^2 S3^2 - a^2 c^4 S1^2 S3^2 + b^2 c^4 S1^2 S3^2 - a^2 b^2 d^2 S1^2 S3^2 +
    b^4 d^2 S1^2 S3^2 + a^2 c^2 d^2 S1^2 S3^2 - b^2 c^2 d^2 S1^2 S3^2 + a^2 b^2 c^2 S0 S2 S3^2 - b^4 c^2 S0 S2 S3^2 +
    a^2 c^4 S0 S2 S3^2 - b^2 c^4 S0 S2 S3^2 - a^2 b^2 d^2 S0 S2 S3^2 + b^4 d^2 S0 S2 S3^2 - a^2 c^2 d^2 S0 S2 S3^2 +
    b^2 c^2 d^2 S0 S2 S3^2 + a^2 b^2 c^2 S1 S2 S3^2 + b^4 c^2 S1 S2 S3^2 + a^2 c^4 S1 S2 S3^2 + b^2 c^4 S1 S2 S3^2 -
    a^2 b^2 d^2 S1 S2 S3^2 - b^4 d^2 S1 S2 S3^2 - a^2 c^2 d^2 S1 S2 S3^2 - b^2 c^2 d^2 S1 S2 S3^2 +
    a^2 b^2 c^2 S0 S1 S2 S4 + b^4 c^2 S0 S1 S2 S4 + a^2 c^4 S0 S1 S2 S4 + b^2 c^4 S0 S1 S2 S4 -
    a^2 b^2 d^2 S0 S1 S2 S4 - b^4 d^2 S0 S1 S2 S4 - a^2 c^2 d^2 S0 S1 S2 S4 - b^2 c^2 d^2 S0 S1 S2 S4 +
    a^2 b^2 c^2 S1^2 S2 S4 - b^4 c^2 S1^2 S2 S4 + a^2 c^4 S1^2 S2 S4 - b^2 c^4 S1^2 S2 S4 - a^2 b^2 d^2 S1^2 S2 S4 +
    b^4 d^2 S1^2 S2 S4 - a^2 c^2 d^2 S1^2 S2 S4 + b^2 c^2 d^2 S1^2 S2 S4 + a^2 b^2 c^2 S0 S2^2 S4 -
    b^4 c^2 S0 S2^2 S4 - a^2 c^4 S0 S2^2 S4 + b^2 c^4 S0 S2^2 S4 - a^2 b^2 d^2 S0 S2^2 S4 + b^4 d^2 S0 S2^2 S4 +
    a^2 c^2 d^2 S0 S2^2 S4 - b^2 c^2 d^2 S0 S2^2 S4 + a^2 b^2 c^2 S1 S2^2 S4 + b^4 c^2 S1 S2^2 S4 -
    a^2 c^4 S1 S2^2 S4 - b^2 c^4 S1 S2^2 S4 - a^2 b^2 d^2 S1 S2^2 S4 - b^4 d^2 S1 S2^2 S4 + a^2 c^2 d^2 S1 S2^2 S4 +
    b^2 c^2 d^2 S1 S2^2 S4 + a^2 b^2 c^2 S0 S1 S3 S4 + b^4 c^2 S0 S1 S3 S4 - a^2 c^4 S0 S1 S3 S4 -
    b^2 c^4 S0 S1 S3 S4 + a^2 b^2 d^2 S0 S1 S3 S4 + b^4 d^2 S0 S1 S3 S4 - a^2 c^2 d^2 S0 S1 S3 S4 -
    b^2 c^2 d^2 S0 S1 S3 S4 + a^2 b^2 c^2 S1^2 S3 S4 - b^4 c^2 S1^2 S3 S4 - a^2 c^4 S1^2 S3 S4 + b^2 c^4 S1^2 S3 S4 +
    a^2 b^2 d^2 S1^2 S3 S4 - b^4 d^2 S1^2 S3 S4 - a^2 c^2 d^2 S1^2 S3 S4 + b^2 c^2 d^2 S1^2 S3 S4 +
    a^2 b^2 c^2 S0 S2 S3 S4 - b^4 c^2 S0 S2 S3 S4 + a^2 c^4 S0 S2 S3 S4 - b^2 c^4 S0 S2 S3 S4 +
    a^2 b^2 d^2 S0 S2 S3 S4 - b^4 d^2 S0 S2 S3 S4 + a^2 c^2 d^2 S0 S2 S3 S4 - b^2 c^2 d^2 S0 S2 S3 S4 +
    a^2 b^2 c^2 S1 S2 S3 S4 + b^4 c^2 S1 S2 S3 S4 + a^2 c^4 S1 S2 S3 S4 + b^2 c^4 S1 S2 S3 S4 +
    a^2 b^2 d^2 S1 S2 S3 S4 + b^4 d^2 S1 S2 S3 S4 + a^2 c^2 d^2 S1 S2 S3 S4 + b^2 c^2 d^2 S1 S2 S3 S4))
```

FullSimplify[%28]

```
-((4 a^2 b^2 E0 S0 S1 (d^2 r^2 (S2 - S3) (S3 - S4) +
      c^4 (S2 - S3) (S3 + S4) + c^2 (S2 + S3) (d^2 (S3 - S4) + r^2 (S3 + S4))) Cos[θ]) /
  (r (b^2 (S0 - S1) (c^2 (S1 + S2) (d^2 (S2 + S3) (S3 - S4) + c^2 (S2 - S3) (S3 + S4)) +
        b^2 (S1 - S2) (d^2 (S2 - S3) (S3 - S4) + c^2 (S2 + S3) (S3 + S4))) +
      a^2 (S0 + S1) (c^2 (S1 - S2) (d^2 (S2 + S3) (S3 - S4) + c^2 (S2 - S3) (S3 + S4)) +
        b^2 (S1 + S2) (d^2 (S2 - S3) (S3 - S4) + c^2 (S2 + S3) (S3 + S4))))))
```

V3[r, θ]

```
-((8 b^2 c^2 E0 r S0 S2 (a^2 S1 S3 + a^2 S1 S4) Cos[θ]) /
   (a^2 b^2 c^2 S0 S1 S2 S3 + b^4 c^2 S0 S1 S2 S3 + a^2 c^4 S0 S1 S2 S3 + b^2 c^4 S0 S1 S2 S3 +
    a^2 b^2 d^2 S0 S1 S2 S3 + b^4 d^2 S0 S1 S2 S3 + a^2 c^2 d^2 S0 S1 S2 S3 + b^2 c^2 d^2 S0 S1 S2 S3 +
    a^2 b^2 c^2 S1^2 S2 S3 - b^4 c^2 S1^2 S2 S3 + a^2 c^4 S1^2 S2 S3 - b^2 c^4 S1^2 S2 S3 + a^2 b^2 d^2 S1^2 S2 S3 -
```

$b^4 d^2 S1^2 S2 S3 + a^2 c^2 d^2 S1^2 S2 S3 - b^2 c^2 d^2 S1^2 S2 S3 + a^2 b^2 c^2 S0 S2^2 S3 -$
$b^4 c^2 S0 S2^2 S3 - a^2 c^4 S0 S2^2 S3 + b^2 c^4 S0 S2^2 S3 + a^2 b^2 d^2 S0 S2^2 S3 - b^4 d^2 S0 S2^2 S3 -$
$a^2 c^2 d^2 S0 S2^2 S3 + b^2 c^2 d^2 S0 S2^2 S3 + a^2 b^2 c^2 S1 S2^2 S3 + b^4 c^2 S1 S2^2 S3 -$
$a^2 c^4 S1 S2^2 S3 - b^2 c^4 S1 S2^2 S3 + a^2 b^2 d^2 S1 S2^2 S3 + b^4 d^2 S1 S2^2 S3 - a^2 c^2 d^2 S1 S2^2 S3 -$
$b^2 c^2 d^2 S1 S2^2 S3 + a^2 b^2 c^2 S0 S1 S3^2 + b^4 c^2 S0 S1 S3^2 - a^2 c^4 S0 S1 S3^2 - b^2 c^4 S0 S1 S3^2 -$
$a^2 b^2 d^2 S0 S1 S3^2 - b^4 d^2 S0 S1 S3^2 + a^2 c^2 d^2 S0 S1 S3^2 + b^2 c^2 d^2 S0 S1 S3^2 +$
$a^2 b^2 c^2 S1^2 S3^2 - b^4 c^2 S1^2 S3^2 - a^2 c^4 S1^2 S3^2 + b^2 c^4 S1^2 S3^2 - a^2 b^2 d^2 S1^2 S3^2 +$
$b^4 d^2 S1^2 S3^2 + a^2 c^2 d^2 S1^2 S3^2 - b^2 c^2 d^2 S1^2 S3^2 + a^2 b^2 c^2 S0 S2 S3^2 - b^4 c^2 S0 S2 S3^2 +$
$a^2 c^4 S0 S2 S3^2 - b^2 c^4 S0 S2 S3^2 - a^2 b^2 d^2 S0 S2 S3^2 + b^4 d^2 S0 S2 S3^2 - a^2 c^2 d^2 S0 S2 S3^2 +$
$b^2 c^2 d^2 S0 S2 S3^2 + a^2 b^2 c^2 S1 S2 S3^2 + b^4 c^2 S1 S2 S3^2 + a^2 c^4 S1 S2 S3^2 + b^2 c^4 S1 S2 S3^2 -$
$a^2 b^2 d^2 S1 S2 S3^2 - b^4 d^2 S1 S2 S3^2 - a^2 c^2 d^2 S1 S2 S3^2 - b^2 c^2 d^2 S1 S2 S3^2 +$
$a^2 b^2 c^2 S0 S1 S2 S4 + b^4 c^2 S0 S1 S2 S4 + a^2 c^4 S0 S1 S2 S4 + b^2 c^4 S0 S1 S2 S4 -$
$a^2 b^2 d^2 S0 S1 S2 S4 - b^4 d^2 S0 S1 S2 S4 - a^2 c^2 d^2 S0 S1 S2 S4 - b^2 c^2 d^2 S0 S1 S2 S4 +$
$a^2 b^2 c^2 S1^2 S2 S4 - b^4 c^2 S1^2 S2 S4 + a^2 c^4 S1^2 S2 S4 - b^2 c^4 S1^2 S2 S4 - a^2 b^2 d^2 S1^2 S2 S4 +$
$b^4 d^2 S1^2 S2 S4 - a^2 c^2 d^2 S1^2 S2 S4 + b^2 c^2 d^2 S1^2 S2 S4 + a^2 b^2 c^2 S0 S2^2 S4 -$
$b^4 c^2 S0 S2^2 S4 - a^2 c^4 S0 S2^2 S4 + b^2 c^4 S0 S2^2 S4 - a^2 b^2 d^2 S0 S2^2 S4 + b^4 d^2 S0 S2^2 S4 +$
$a^2 c^2 d^2 S0 S2^2 S4 - b^2 c^2 d^2 S0 S2^2 S4 + a^2 b^2 c^2 S1 S2^2 S4 + b^4 c^2 S1 S2^2 S4 -$
$a^2 c^4 S1 S2^2 S4 - b^2 c^4 S1 S2^2 S4 - a^2 b^2 d^2 S1 S2^2 S4 - b^4 d^2 S1 S2^2 S4 + a^2 c^2 d^2 S1 S2^2 S4 +$
$b^2 c^2 d^2 S1 S2^2 S4 + a^2 b^2 c^2 S0 S1 S3 S4 + b^4 c^2 S0 S1 S3 S4 - a^2 c^4 S0 S1 S3 S4 -$
$b^2 c^4 S0 S1 S3 S4 + a^2 b^2 d^2 S0 S1 S3 S4 + b^4 d^2 S0 S1 S3 S4 - a^2 c^2 d^2 S0 S1 S3 S4 -$
$b^2 c^2 d^2 S0 S1 S3 S4 + a^2 b^2 c^2 S1^2 S3 S4 - b^4 c^2 S1^2 S3 S4 - a^2 c^4 S1^2 S3 S4 + b^2 c^4 S1^2 S3 S4 +$
$a^2 b^2 d^2 S1^2 S3 S4 - b^4 d^2 S1^2 S3 S4 - a^2 c^2 d^2 S1^2 S3 S4 + b^2 c^2 d^2 S1^2 S3 S4 +$
$a^2 b^2 c^2 S0 S2 S3 S4 - b^4 c^2 S0 S2 S3 S4 + a^2 c^4 S0 S2 S3 S4 - b^2 c^4 S0 S2 S3 S4 +$
$a^2 b^2 d^2 S0 S2 S3 S4 - b^4 d^2 S0 S2 S3 S4 + a^2 c^2 d^2 S0 S2 S3 S4 - b^2 c^2 d^2 S0 S2 S3 S4 +$
$a^2 b^2 c^2 S1 S2 S3 S4 + b^4 c^2 S1 S2 S3 S4 + a^2 c^4 S1 S2 S3 S4 + b^2 c^4 S1 S2 S3 S4 +$
$a^2 b^2 d^2 S1 S2 S3 S4 + b^4 d^2 S1 S2 S3 S4 + a^2 c^2 d^2 S1 S2 S3 S4 + b^2 c^2 d^2 S1 S2 S3 S4)) -$
$(8 b^2 E0 S0 S2 (a^2 c^2 d^2 S1 S3 - a^2 c^2 d^2 S1 S4) \mathrm{Cos}[\theta]) /$
$(r (a^2 b^2 c^2 S0 S1 S2 S3 + b^4 c^2 S0 S1 S2 S3 + a^2 c^4 S0 S1 S2 S3 + b^2 c^4 S0 S1 S2 S3 +$
$a^2 b^2 d^2 S0 S1 S2 S3 + b^4 d^2 S0 S1 S2 S3 + a^2 c^2 d^2 S0 S1 S2 S3 + b^2 c^2 d^2 S0 S1 S2 S3 +$
$a^2 b^2 c^2 S1^2 S2 S3 - b^4 c^2 S1^2 S2 S3 + a^2 c^4 S1^2 S2 S3 - b^2 c^4 S1^2 S2 S3 + a^2 b^2 d^2 S1^2 S2 S3 -$
$b^4 d^2 S1^2 S2 S3 + a^2 c^2 d^2 S1^2 S2 S3 - b^2 c^2 d^2 S1^2 S2 S3 + a^2 b^2 c^2 S0 S2^2 S3 -$
$b^4 c^2 S0 S2^2 S3 - a^2 c^4 S0 S2^2 S3 + b^2 c^4 S0 S2^2 S3 + a^2 b^2 d^2 S0 S2^2 S3 - b^4 d^2 S0 S2^2 S3 -$
$a^2 c^2 d^2 S0 S2^2 S3 + b^2 c^2 d^2 S0 S2^2 S3 + a^2 b^2 c^2 S1 S2^2 S3 + b^4 c^2 S1 S2^2 S3 -$
$a^2 c^4 S1 S2^2 S3 - b^2 c^4 S1 S2^2 S3 + a^2 b^2 d^2 S1 S2^2 S3 + b^4 d^2 S1 S2^2 S3 - a^2 c^2 d^2 S1 S2^2 S3 -$
$b^2 c^2 d^2 S1 S2^2 S3 + a^2 b^2 c^2 S0 S1 S3^2 + b^4 c^2 S0 S1 S3^2 - a^2 c^4 S0 S1 S3^2 - b^2 c^4 S0 S1 S3^2 -$
$a^2 b^2 d^2 S0 S1 S3^2 - b^4 d^2 S0 S1 S3^2 + a^2 c^2 d^2 S0 S1 S3^2 + b^2 c^2 d^2 S0 S1 S3^2 +$
$a^2 b^2 c^2 S1^2 S3^2 - b^4 c^2 S1^2 S3^2 - a^2 c^4 S1^2 S3^2 + b^2 c^4 S1^2 S3^2 - a^2 b^2 d^2 S1^2 S3^2 +$
$b^4 d^2 S1^2 S3^2 + a^2 c^2 d^2 S1^2 S3^2 - b^2 c^2 d^2 S1^2 S3^2 + a^2 b^2 c^2 S0 S2 S3^2 - b^4 c^2 S0 S2 S3^2 +$
$a^2 c^4 S0 S2 S3^2 - b^2 c^4 S0 S2 S3^2 - a^2 b^2 d^2 S0 S2 S3^2 + b^4 d^2 S0 S2 S3^2 - a^2 c^2 d^2 S0 S2 S3^2 +$
$b^2 c^2 d^2 S0 S2 S3^2 + a^2 b^2 c^2 S1 S2 S3^2 + b^4 c^2 S1 S2 S3^2 + a^2 c^4 S1 S2 S3^2 + b^2 c^4 S1 S2 S3^2 -$
$a^2 b^2 d^2 S1 S2 S3^2 - b^4 d^2 S1 S2 S3^2 - a^2 c^2 d^2 S1 S2 S3^2 - b^2 c^2 d^2 S1 S2 S3^2 +$
$a^2 b^2 c^2 S0 S1 S2 S4 + b^4 c^2 S0 S1 S2 S4 + a^2 c^4 S0 S1 S2 S4 + b^2 c^4 S0 S1 S2 S4 -$
$a^2 b^2 d^2 S0 S1 S2 S4 - b^4 d^2 S0 S1 S2 S4 - a^2 c^2 d^2 S0 S1 S2 S4 - b^2 c^2 d^2 S0 S1 S2 S4 +$

$$a^2 b^2 c^2 S1^2 S2 S4 - b^4 c^2 S1^2 S2 S4 + a^2 c^4 S1^2 S2 S4 - b^2 c^4 S1^2 S2 S4 - a^2 b^2 d^2 S1^2 S2 S4 +$$
$$b^4 d^2 S1^2 S2 S4 - a^2 c^2 d^2 S1^2 S2 S4 + b^2 c^2 d^2 S1^2 S2 S4 + a^2 b^2 c^2 S0 S2^2 S4 -$$
$$b^4 c^2 S0 S2^2 S4 - a^2 c^4 S0 S2^2 S4 + b^2 c^4 S0 S2^2 S4 - a^2 b^2 d^2 S0 S2^2 S4 + b^4 d^2 S0 S2^2 S4 +$$
$$a^2 c^2 d^2 S0 S2^2 S4 - b^2 c^2 d^2 S0 S2^2 S4 + a^2 b^2 c^2 S1 S2^2 S4 + b^4 c^2 S1 S2^2 S4 -$$
$$a^2 c^4 S1 S2^2 S4 - b^2 c^4 S1 S2^2 S4 - a^2 b^2 d^2 S1 S2^2 S4 - b^4 d^2 S1 S2^2 S4 + a^2 c^2 d^2 S1 S2^2 S4 +$$
$$b^2 c^2 d^2 S1 S2^2 S4 + a^2 b^2 c^2 S0 S1 S3 S4 + b^4 c^2 S0 S1 S3 S4 - a^2 c^4 S0 S1 S3 S4 -$$
$$b^2 c^4 S0 S1 S3 S4 + a^2 b^2 d^2 S0 S1 S3 S4 + b^4 d^2 S0 S1 S3 S4 - a^2 c^2 d^2 S0 S1 S3 S4 -$$
$$b^2 c^2 d^2 S0 S1 S3 S4 + a^2 b^2 c^2 S1^2 S3 S4 - b^4 c^2 S1^2 S3 S4 - a^2 c^4 S1^2 S3 S4 + b^2 c^4 S1^2 S3 S4 +$$
$$a^2 b^2 d^2 S1^2 S3 S4 - b^4 d^2 S1^2 S3 S4 - a^2 c^2 d^2 S1^2 S3 S4 + b^2 c^2 d^2 S1^2 S3 S4 +$$
$$a^2 b^2 c^2 S0 S2 S3 S4 - b^4 c^2 S0 S2 S3 S4 + a^2 c^4 S0 S2 S3 S4 - b^2 c^4 S0 S2 S3 S4 +$$
$$a^2 b^2 d^2 S0 S2 S3 S4 - b^4 d^2 S0 S2 S3 S4 + a^2 c^2 d^2 S0 S2 S3 S4 - b^2 c^2 d^2 S0 S2 S3 S4 +$$
$$a^2 b^2 c^2 S1 S2 S3 S4 + b^4 c^2 S1 S2 S3 S4 + a^2 c^4 S1 S2 S3 S4 + b^2 c^4 S1 S2 S3 S4 +$$
$$a^2 b^2 d^2 S1 S2 S3 S4 + b^4 d^2 S1 S2 S3 S4 + a^2 c^2 d^2 S1 S2 S3 S4 + b^2 c^2 d^2 S1 S2 S3 S4))$$

```
FullSimplify[%30]
```

$$-\Big(\big(8\, a^2 b^2 c^2\, E0\, S0\, S1\, S2 \left(d^2 (S3 - S4) + r^2 (S3 + S4)\right) \mathrm{Cos}[\theta]\big) \Big/$$
$$\Big(r \Big(b^2 (S0 - S1) \Big(c^2 (S1 + S2) \left(d^2 (S2 + S3) (S3 - S4) + c^2 (S2 - S3) (S3 + S4)\right) +$$
$$b^2 (S1 - S2) \left(d^2 (S2 - S3) (S3 - S4) + c^2 (S2 + S3) (S3 + S4)\right)\Big) +$$
$$a^2 (S0 + S1) \Big(c^2 (S1 - S2) \left(d^2 (S2 + S3) (S3 - S4) + c^2 (S2 - S3) (S3 + S4)\right) +$$
$$b^2 (S1 + S2) \left(d^2 (S2 - S3) (S3 - S4) + c^2 (S2 + S3) (S3 + S4)\right)\Big)\Big)\Big)\Big)$$

```
V4[r, θ]
-((16 a^2 b^2 c^2 E0 r S0 S1 S2 S3 Cos[θ]) /
   (a^2 b^2 c^2 S0 S1 S2 S3 + b^4 c^2 S0 S1 S2 S3 + a^2 c^4 S0 S1 S2 S3 + b^2 c^4 S0 S1 S2 S3 +
     a^2 b^2 d^2 S0 S1 S2 S3 + b^4 d^2 S0 S1 S2 S3 + a^2 c^2 d^2 S0 S1 S2 S3 + b^2 c^2 d^2 S0 S1 S2 S3 +
     a^2 b^2 c^2 S1^2 S2 S3 - b^4 c^2 S1^2 S2 S3 + a^2 c^4 S1^2 S2 S3 - b^2 c^4 S1^2 S2 S3 + a^2 b^2 d^2 S1^2 S2 S3 -
     b^4 d^2 S1^2 S2 S3 + a^2 c^2 d^2 S1^2 S2 S3 - b^2 c^2 d^2 S1^2 S2 S3 + a^2 b^2 c^2 S0 S2^2 S3 -
     b^4 c^2 S0 S2^2 S3 - a^2 c^4 S0 S2^2 S3 + b^2 c^4 S0 S2^2 S3 + a^2 b^2 d^2 S0 S2^2 S3 - b^4 d^2 S0 S2^2 S3 -
     a^2 c^2 d^2 S0 S2^2 S3 + b^2 c^2 d^2 S0 S2^2 S3 + a^2 b^2 c^2 S1 S2^2 S3 + b^4 c^2 S1 S2^2 S3 -
     a^2 c^4 S1 S2^2 S3 - b^2 c^4 S1 S2^2 S3 + a^2 b^2 d^2 S1 S2^2 S3 + b^4 d^2 S1 S2^2 S3 - a^2 c^2 d^2 S1 S2^2 S3 -
     b^2 c^2 d^2 S1 S2^2 S3 + a^2 b^2 c^2 S0 S1 S3^2 + b^4 c^2 S0 S1 S3^2 - a^2 c^4 S0 S1 S3^2 - b^2 c^4 S0 S1 S3^2 -
     a^2 b^2 d^2 S0 S1 S3^2 - b^4 d^2 S0 S1 S3^2 + a^2 c^2 d^2 S0 S1 S3^2 + b^2 c^2 d^2 S0 S1 S3^2 +
     a^2 b^2 c^2 S1^2 S3^2 - b^4 c^2 S1^2 S3^2 - a^2 c^4 S1^2 S3^2 + b^2 c^4 S1^2 S3^2 - a^2 b^2 d^2 S1^2 S3^2 +
     b^4 d^2 S1^2 S3^2 + a^2 c^2 d^2 S1^2 S3^2 - b^2 c^2 d^2 S1^2 S3^2 + a^2 b^2 c^2 S0 S2 S3^2 - b^4 c^2 S0 S2 S3^2 +
     a^2 c^4 S0 S2 S3^2 - b^2 c^4 S0 S2 S3^2 - a^2 b^2 d^2 S0 S2 S3^2 + b^4 d^2 S0 S2 S3^2 - a^2 c^2 d^2 S0 S2 S3^2 +
     b^2 c^2 d^2 S0 S2 S3^2 + a^2 b^2 c^2 S1 S2 S3^2 + b^4 c^2 S1 S2 S3^2 + a^2 c^4 S1 S2 S3^2 + b^2 c^4 S1 S2 S3^2 -
     a^2 b^2 d^2 S1 S2 S3^2 - b^4 d^2 S1 S2 S3^2 - a^2 c^2 d^2 S1 S2 S3^2 - b^2 c^2 d^2 S1 S2 S3^2 +
     a^2 b^2 c^2 S0 S1 S2 S4 + b^4 c^2 S0 S1 S2 S4 + a^2 c^4 S0 S1 S2 S4 + b^2 c^4 S0 S1 S2 S4 -
     a^2 b^2 d^2 S0 S1 S2 S4 - b^4 d^2 S0 S1 S2 S4 - a^2 c^2 d^2 S0 S1 S2 S4 - b^2 c^2 d^2 S0 S1 S2 S4 +
     a^2 b^2 c^2 S1^2 S2 S4 - b^4 c^2 S1^2 S2 S4 + a^2 c^4 S1^2 S2 S4 - b^2 c^4 S1^2 S2 S4 - a^2 b^2 d^2 S1^2 S2 S4 +
     b^4 d^2 S1^2 S2 S4 - a^2 c^2 d^2 S1^2 S2 S4 + b^2 c^2 d^2 S1^2 S2 S4 + a^2 b^2 c^2 S0 S2^2 S4 -
     b^4 c^2 S0 S2^2 S4 - a^2 c^4 S0 S2^2 S4 + b^2 c^4 S0 S2^2 S4 - a^2 b^2 d^2 S0 S2^2 S4 + b^4 d^2 S0 S2^2 S4 +
     a^2 c^2 d^2 S0 S2^2 S4 - b^2 c^2 d^2 S0 S2^2 S4 + a^2 b^2 c^2 S1 S2^2 S4 + b^4 c^2 S1 S2^2 S4 -
     a^2 c^4 S1 S2^2 S4 - b^2 c^4 S1 S2^2 S4 - a^2 b^2 d^2 S1 S2^2 S4 - b^4 d^2 S1 S2^2 S4 + a^2 c^2 d^2 S1 S2^2 S4 +
     b^2 c^2 d^2 S1 S2^2 S4 + a^2 b^2 c^2 S0 S1 S3 S4 + b^4 c^2 S0 S1 S3 S4 - a^2 c^4 S0 S1 S3 S4 -
     b^2 c^4 S0 S1 S3 S4 + a^2 b^2 d^2 S0 S1 S3 S4 + b^4 d^2 S0 S1 S3 S4 - a^2 c^2 d^2 S0 S1 S3 S4 -
     b^2 c^2 d^2 S0 S1 S3 S4 + a^2 b^2 c^2 S1^2 S3 S4 - b^4 c^2 S1^2 S3 S4 - a^2 c^4 S1^2 S3 S4 + b^2 c^4 S1^2 S3 S4 +
     a^2 b^2 d^2 S1^2 S3 S4 - b^4 d^2 S1^2 S3 S4 - a^2 c^2 d^2 S1^2 S3 S4 + b^2 c^2 d^2 S1^2 S3 S4 +
     a^2 b^2 c^2 S0 S2 S3 S4 - b^4 c^2 S0 S2 S3 S4 + a^2 c^4 S0 S2 S3 S4 - b^2 c^4 S0 S2 S3 S4 +
     a^2 b^2 d^2 S0 S2 S3 S4 - b^4 d^2 S0 S2 S3 S4 + a^2 c^2 d^2 S0 S2 S3 S4 - b^2 c^2 d^2 S0 S2 S3 S4 +
     a^2 b^2 c^2 S1 S2 S3 S4 + b^4 c^2 S1 S2 S3 S4 + a^2 c^4 S1 S2 S3 S4 + b^2 c^4 S1 S2 S3 S4 +
     a^2 b^2 d^2 S1 S2 S3 S4 + b^4 d^2 S1 S2 S3 S4 + a^2 c^2 d^2 S1 S2 S3 S4 + b^2 c^2 d^2 S1 S2 S3 S4))

FullSimplify[%32]
-((16 a^2 b^2 c^2 E0 r S0 S1 S2 S3 Cos[θ]) /
   (b^2 (S0 - S1) (c^2 (S1 + S2) (d^2 (S2 + S3) (S3 - S4) + c^2 (S2 - S3) (S3 + S4)) +
        b^2 (S1 - S2) (d^2 (S2 - S3) (S3 - S4) + c^2 (S2 + S3) (S3 + S4))) +
     a^2 (S0 + S1) (c^2 (S1 - S2) (d^2 (S2 + S3) (S3 - S4) + c^2 (S2 - S3) (S3 + S4)) +
        b^2 (S1 + S2) (d^2 (S2 - S3) (S3 - S4) + c^2 (S2 + S3) (S3 + S4)))))

/* Step 3 : Expression for voltage (V3) on each side of the axonal membrane */
```

```
V3 = - ((8 a^2 b^2 c^2 E0 S0 S1 S2 (d^2 (S3 - S4) + r^2 (S3 + S4)) Cos[θ]) /
    (r (b^2 (S0 - S1) (c^2 (S1 + S2) (d^2 (S2 + S3) (S3 - S4) + c^2 (S2 - S3) (S3 + S4)) +
            b^2 (S1 - S2) (d^2 (S2 - S3) (S3 - S4) + c^2 (S2 + S3) (S3 + S4))) +
         a^2 (S0 + S1) (c^2 (S1 - S2) (d^2 (S2 + S3) (S3 - S4) + c^2 (S2 - S3) (S3 + S4)) +
            b^2 (S1 + S2) (d^2 (S2 - S3) (S3 - S4) + c^2 (S2 + S3) (S3 + S4))))))
```

```
r = c
```

```
c
```

```
V3out = - ((8 a^2 b^2 c^2 E0 S0 S1 S2 (d^2 (S3 - S4) + r^2 (S3 + S4)) Cos[θ]) /
    (r (b^2 (S0 - S1) (c^2 (S1 + S2) (d^2 (S2 + S3) (S3 - S4) + c^2 (S2 - S3) (S3 + S4)) +
            b^2 (S1 - S2) (d^2 (S2 - S3) (S3 - S4) + c^2 (S2 + S3) (S3 + S4))) +
         a^2 (S0 + S1) (c^2 (S1 - S2) (d^2 (S2 + S3) (S3 - S4) + c^2 (S2 - S3) (S3 + S4)) +
            b^2 (S1 + S2) (d^2 (S2 - S3) (S3 - S4) + c^2 (S2 + S3) (S3 + S4))))))
```

```
- ((8 a^2 b^2 c E0 S0 S1 S2 (d^2 (S3 - S4) + c^2 (S3 + S4)) Cos[θ]) /
    (b^2 (S0 - S1) (c^2 (S1 + S2) (d^2 (S2 + S3) (S3 - S4) + c^2 (S2 - S3) (S3 + S4)) +
          b^2 (S1 - S2) (d^2 (S2 - S3) (S3 - S4) + c^2 (S2 + S3) (S3 + S4))) +
       a^2 (S0 + S1) (c^2 (S1 - S2) (d^2 (S2 + S3) (S3 - S4) + c^2 (S2 - S3) (S3 + S4)) +
          b^2 (S1 + S2) (d^2 (S2 - S3) (S3 - S4) + c^2 (S2 + S3) (S3 + S4)))))
```

```
FullSimplify[V3out]
```

```
- ((8 a^2 b^2 c E0 S0 S1 S2 (d^2 (S3 - S4) + c^2 (S3 + S4)) Cos[θ]) /
    (b^2 (S0 - S1) (c^2 (S1 + S2) (d^2 (S2 + S3) (S3 - S4) + c^2 (S2 - S3) (S3 + S4)) +
          b^2 (S1 - S2) (d^2 (S2 - S3) (S3 - S4) + c^2 (S2 + S3) (S3 + S4))) +
       a^2 (S0 + S1) (c^2 (S1 - S2) (d^2 (S2 + S3) (S3 - S4) + c^2 (S2 - S3) (S3 + S4)) +
          b^2 (S1 + S2) (d^2 (S2 - S3) (S3 - S4) + c^2 (S2 + S3) (S3 + S4)))))
```

```
r = d
```

```
d
```

```
V3in = - ((8 a^2 b^2 c^2 E0 S0 S1 S2 (d^2 (S3 - S4) + r^2 (S3 + S4)) Cos[θ]) /
    (r (b^2 (S0 - S1) (c^2 (S1 + S2) (d^2 (S2 + S3) (S3 - S4) + c^2 (S2 - S3) (S3 + S4)) +
            b^2 (S1 - S2) (d^2 (S2 - S3) (S3 - S4) + c^2 (S2 + S3) (S3 + S4))) +
         a^2 (S0 + S1) (c^2 (S1 - S2) (d^2 (S2 + S3) (S3 - S4) + c^2 (S2 - S3) (S3 + S4)) +
            b^2 (S1 + S2) (d^2 (S2 - S3) (S3 - S4) + c^2 (S2 + S3) (S3 + S4))))))
```

```
- ((8 a^2 b^2 c^2 E0 S0 S1 S2 (d^2 (S3 - S4) + d^2 (S3 + S4)) Cos[θ]) /
    (d (b^2 (S0 - S1) (c^2 (S1 + S2) (d^2 (S2 + S3) (S3 - S4) + c^2 (S2 - S3) (S3 + S4)) +
            b^2 (S1 - S2) (d^2 (S2 - S3) (S3 - S4) + c^2 (S2 + S3) (S3 + S4))) +
         a^2 (S0 + S1) (c^2 (S1 - S2) (d^2 (S2 + S3) (S3 - S4) + c^2 (S2 - S3) (S3 + S4)) +
            b^2 (S1 + S2) (d^2 (S2 - S3) (S3 - S4) + c^2 (S2 + S3) (S3 + S4))))))
```

```
FullSimplify[V3in]
-((16 a^2 b^2 c^2 d E0 S0 S1 S2 S3 Cos[θ]) /
    (b^2 (S0 - S1) (c^2 (S1 + S2) (d^2 (S2 + S3) (S3 - S4) + c^2 (S2 - S3) (S3 + S4)) +
          b^2 (S1 - S2) (d^2 (S2 - S3) (S3 - S4) + c^2 (S2 + S3) (S3 + S4))) +
       a^2 (S0 + S1) (c^2 (S1 - S2) (d^2 (S2 + S3) (S3 - S4) + c^2 (S2 - S3) (S3 + S4)) +
          b^2 (S1 + S2) (d^2 (S2 - S3) (S3 - S4) + c^2 (S2 + S3) (S3 + S4)))))
```

```
/* Step 4 : Transmembrane potential calculaton,
which is defined as the difference between V3in and V3out. */
```

```
Vm = V3in - V3out
(8 a^2 b^2 c E0 S0 S1 S2 (d^2 (S3 - S4) + c^2 (S3 + S4)) Cos[θ]) /
   (b^2 (S0 - S1) (c^2 (S1 + S2) (d^2 (S2 + S3) (S3 - S4) + c^2 (S2 - S3) (S3 + S4)) +
          b^2 (S1 - S2) (d^2 (S2 - S3) (S3 - S4) + c^2 (S2 + S3) (S3 + S4))) +
      a^2 (S0 + S1) (c^2 (S1 - S2) (d^2 (S2 + S3) (S3 - S4) + c^2 (S2 - S3) (S3 + S4)) +
          b^2 (S1 + S2) (d^2 (S2 - S3) (S3 - S4) + c^2 (S2 + S3) (S3 + S4)))) -
  (8 a^2 b^2 c^2 E0 S0 S1 S2 (d^2 (S3 - S4) + d^2 (S3 + S4)) Cos[θ]) /
   (d (b^2 (S0 - S1) (c^2 (S1 + S2) (d^2 (S2 + S3) (S3 - S4) + c^2 (S2 - S3) (S3 + S4)) +
             b^2 (S1 - S2) (d^2 (S2 - S3) (S3 - S4) + c^2 (S2 + S3) (S3 + S4))) +
        a^2 (S0 + S1) (c^2 (S1 - S2) (d^2 (S2 + S3) (S3 - S4) + c^2 (S2 - S3) (S3 + S4)) +
             b^2 (S1 + S2) (d^2 (S2 - S3) (S3 - S4) + c^2 (S2 + S3) (S3 + S4)))))
```

```
/* Expression of Vm in equation (11). */
```

```
FullSimplify[Vm]
(8 a^2 b^2 c (c - d) E0 S0 S1 S2 (d (-S3 + S4) + c (S3 + S4)) Cos[θ]) /
  (b^2 (S0 - S1) (c^2 (S1 + S2) (d^2 (S2 + S3) (S3 - S4) + c^2 (S2 - S3) (S3 + S4)) +
        b^2 (S1 - S2) (d^2 (S2 - S3) (S3 - S4) + c^2 (S2 + S3) (S3 + S4))) +
     a^2 (S0 + S1) (c^2 (S1 - S2) (d^2 (S2 + S3) (S3 - S4) + c^2 (S2 - S3) (S3 + S4)) +
        b^2 (S1 + S2) (d^2 (S2 - S3) (S3 - S4) + c^2 (S2 + S3) (S3 + S4))))
```
