# Supplementary material for: Shielding effects of myelin sheath on axolemma depolarization under transverse electric field stimulation
Source: PeerJ. 2018 Dec 3;6:e6020. doi: 10.7717/peerj.6020 (PMC6282940; doi:10.7717/peerj.6020)
Supplement: Supplemental Information 2 [file peerj-06-6020-s002.pdf]

```
/* Voltage drop across the myelin sheath of an myelinated axon,
under a transverse electric field exposure * /

/ * Step 1 : voltage distributation inside the myelin sheath. * /

V1 = - ((2 a^2 E0 S0 (c^2 r^2 (S1 - S2) (d^2 (S2 + S3) (S3 - S4) + c^2 (S2 - S3) (S3 + S4)) +
        b^4 (S1 - S2) (d^2 (S2 - S3) (S3 - S4) + c^2 (S2 + S3) (S3 + S4)) +
        b^2 (S1 + S2) (d^2 r^2 (S2 - S3) (S3 - S4) + c^4 (S2 - S3) (S3 + S4) +
           c^2 (S2 + S3) (d^2 (S3 - S4) + r^2 (S3 + S4)))) Cos[θ]) /
     (r (b^2 (S0 - S1) (c^2 (S1 + S2) (d^2 (S2 + S3) (S3 - S4) + c^2 (S2 - S3) (S3 + S4)) +
           b^2 (S1 - S2) (d^2 (S2 - S3) (S3 - S4) + c^2 (S2 + S3) (S3 + S4))) +
        a^2 (S0 + S1) (c^2 (S1 - S2) (d^2 (S2 + S3) (S3 - S4) + c^2 (S2 - S3) (S3 + S4)) +
           b^2 (S1 + S2) (d^2 (S2 - S3) (S3 - S4) + c^2 (S2 + S3) (S3 + S4)))))

- ((2 a^2 E0 S0 (c^2 r^2 (S1 - S2) (d^2 (S2 + S3) (S3 - S4) + c^2 (S2 - S3) (S3 + S4)) +
        b^4 (S1 - S2) (d^2 (S2 - S3) (S3 - S4) + c^2 (S2 + S3) (S3 + S4)) +
        b^2 (S1 + S2) (d^2 r^2 (S2 - S3) (S3 - S4) + c^4 (S2 - S3) (S3 + S4) +
           c^2 (S2 + S3) (d^2 (S3 - S4) + r^2 (S3 + S4)))) Cos[θ]) /
     (r (b^2 (S0 - S1) (c^2 (S1 + S2) (d^2 (S2 + S3) (S3 - S4) + c^2 (S2 - S3) (S3 + S4)) +
           b^2 (S1 - S2) (d^2 (S2 - S3) (S3 - S4) + c^2 (S2 + S3) (S3 + S4))) +
        a^2 (S0 + S1) (c^2 (S1 - S2) (d^2 (S2 + S3) (S3 - S4) + c^2 (S2 - S3) (S3 + S4)) +
           b^2 (S1 + S2) (d^2 (S2 - S3) (S3 - S4) + c^2 (S2 + S3) (S3 + S4)))))

r = a

a

V1out =
 - ((2 a^2 E0 S0 (c^2 r^2 (S1 - S2) (d^2 (S2 + S3) (S3 - S4) + c^2 (S2 - S3) (S3 + S4)) + b^4 (S1 - S2)
         (d^2 (S2 - S3) (S3 - S4) + c^2 (S2 + S3) (S3 + S4)) +
        b^2 (S1 + S2) (d^2 r^2 (S2 - S3) (S3 - S4) + c^4 (S2 - S3) (S3 + S4) +
           c^2 (S2 + S3) (d^2 (S3 - S4) + r^2 (S3 + S4)))) Cos[θ]) /
     (r (b^2 (S0 - S1) (c^2 (S1 + S2) (d^2 (S2 + S3) (S3 - S4) + c^2 (S2 - S3) (S3 + S4)) +
           b^2 (S1 - S2) (d^2 (S2 - S3) (S3 - S4) + c^2 (S2 + S3) (S3 + S4))) +
        a^2 (S0 + S1) (c^2 (S1 - S2) (d^2 (S2 + S3) (S3 - S4) + c^2 (S2 - S3) (S3 + S4)) +
           b^2 (S1 + S2) (d^2 (S2 - S3) (S3 - S4) + c^2 (S2 + S3) (S3 + S4)))))

- ((2 a E0 S0 (a^2 c^2 (S1 - S2) (d^2 (S2 + S3) (S3 - S4) + c^2 (S2 - S3) (S3 + S4)) +
        b^4 (S1 - S2) (d^2 (S2 - S3) (S3 - S4) + c^2 (S2 + S3) (S3 + S4)) +
        b^2 (S1 + S2) (a^2 d^2 (S2 - S3) (S3 - S4) + c^4 (S2 - S3) (S3 + S4) +
           c^2 (S2 + S3) (d^2 (S3 - S4) + a^2 (S3 + S4)))) Cos[θ]) /
     (b^2 (S0 - S1) (c^2 (S1 + S2) (d^2 (S2 + S3) (S3 - S4) + c^2 (S2 - S3) (S3 + S4)) +
         b^2 (S1 - S2) (d^2 (S2 - S3) (S3 - S4) + c^2 (S2 + S3) (S3 + S4))) +
      a^2 (S0 + S1) (c^2 (S1 - S2) (d^2 (S2 + S3) (S3 - S4) + c^2 (S2 - S3) (S3 + S4)) +
         b^2 (S1 + S2) (d^2 (S2 - S3) (S3 - S4) + c^2 (S2 + S3) (S3 + S4)))))
```

```
r = b
```

```
b
```

```
V1in =
 -((2 a^2 E0 S0 (c^2 r^2 (S1 - S2) (d^2 (S2 + S3) (S3 - S4) + c^2 (S2 - S3) (S3 + S4)) + b^4 (S1 - S2)
          (d^2 (S2 - S3) (S3 - S4) + c^2 (S2 + S3) (S3 + S4)) +
         b^2 (S1 + S2) (d^2 r^2 (S2 - S3) (S3 - S4) + c^4 (S2 - S3) (S3 + S4) +
            c^2 (S2 + S3) (d^2 (S3 - S4) + r^2 (S3 + S4)))) Cos[θ]) /
     (r (b^2 (S0 - S1) (c^2 (S1 + S2) (d^2 (S2 + S3) (S3 - S4) + c^2 (S2 - S3) (S3 + S4)) +
            b^2 (S1 - S2) (d^2 (S2 - S3) (S3 - S4) + c^2 (S2 + S3) (S3 + S4))) +
         a^2 (S0 + S1) (c^2 (S1 - S2) (d^2 (S2 + S3) (S3 - S4) + c^2 (S2 - S3) (S3 + S4)) +
            b^2 (S1 + S2) (d^2 (S2 - S3) (S3 - S4) + c^2 (S2 + S3) (S3 + S4))))))
```

```
-((2 a^2 E0 S0 (b^2 c^2 (S1 - S2) (d^2 (S2 + S3) (S3 - S4) + c^2 (S2 - S3) (S3 + S4)) +
        b^4 (S1 - S2) (d^2 (S2 - S3) (S3 - S4) + c^2 (S2 + S3) (S3 + S4)) +
        b^2 (S1 + S2) (b^2 d^2 (S2 - S3) (S3 - S4) + c^4 (S2 - S3) (S3 + S4) +
           c^2 (S2 + S3) (d^2 (S3 - S4) + b^2 (S3 + S4)))) Cos[θ]) /
   (b (b^2 (S0 - S1) (c^2 (S1 + S2) (d^2 (S2 + S3) (S3 - S4) + c^2 (S2 - S3) (S3 + S4)) +
           b^2 (S1 - S2) (d^2 (S2 - S3) (S3 - S4) + c^2 (S2 + S3) (S3 + S4))) +
        a^2 (S0 + S1) (c^2 (S1 - S2) (d^2 (S2 + S3) (S3 - S4) + c^2 (S2 - S3) (S3 + S4)) +
           b^2 (S1 + S2) (d^2 (S2 - S3) (S3 - S4) + c^2 (S2 + S3) (S3 + S4))))))
```

```
/ Step 2 : Voltage drop across the myelin sheath is
  defined as the voltage difference between the two sides. * /
```

```
Vmyelin = V1in - V1out
```

```
(2 a E0 S0 (a^2 c^2 (S1 - S2) (d^2 (S2 + S3) (S3 - S4) + c^2 (S2 - S3) (S3 + S4)) +
        b^4 (S1 - S2) (d^2 (S2 - S3) (S3 - S4) + c^2 (S2 + S3) (S3 + S4)) +
        b^2 (S1 + S2) (a^2 d^2 (S2 - S3) (S3 - S4) + c^4 (S2 - S3) (S3 + S4) +
           c^2 (S2 + S3) (d^2 (S3 - S4) + a^2 (S3 + S4)))) Cos[θ]) /
  (b^2 (S0 - S1) (c^2 (S1 + S2) (d^2 (S2 + S3) (S3 - S4) + c^2 (S2 - S3) (S3 + S4)) +
        b^2 (S1 - S2) (d^2 (S2 - S3) (S3 - S4) + c^2 (S2 + S3) (S3 + S4))) +
     a^2 (S0 + S1) (c^2 (S1 - S2) (d^2 (S2 + S3) (S3 - S4) + c^2 (S2 - S3) (S3 + S4)) +
        b^2 (S1 + S2) (d^2 (S2 - S3) (S3 - S4) + c^2 (S2 + S3) (S3 + S4)))) -
 (2 a^2 E0 S0 (b^2 c^2 (S1 - S2) (d^2 (S2 + S3) (S3 - S4) + c^2 (S2 - S3) (S3 + S4)) +
        b^4 (S1 - S2) (d^2 (S2 - S3) (S3 - S4) + c^2 (S2 + S3) (S3 + S4)) +
        b^2 (S1 + S2) (b^2 d^2 (S2 - S3) (S3 - S4) + c^4 (S2 - S3) (S3 + S4) +
           c^2 (S2 + S3) (d^2 (S3 - S4) + b^2 (S3 + S4)))) Cos[θ]) /
  (b (b^2 (S0 - S1) (c^2 (S1 + S2) (d^2 (S2 + S3) (S3 - S4) + c^2 (S2 - S3) (S3 + S4)) +
           b^2 (S1 - S2) (d^2 (S2 - S3) (S3 - S4) + c^2 (S2 + S3) (S3 + S4))) +
        a^2 (S0 + S1) (c^2 (S1 - S2) (d^2 (S2 + S3) (S3 - S4) + c^2 (S2 - S3) (S3 + S4)) +
           b^2 (S1 + S2) (d^2 (S2 - S3) (S3 - S4) + c^2 (S2 + S3) (S3 + S4)))))
```

```
FullSimplify[%6]
```

$$\left(2\, a\, (a - b)\, E0\, S0\, \left(b\, \left(c^2\, (S1 + S2)\, \left(d^2\, (S2 + S3)\, (-S3 + S4) - c^2\, (S2 - S3)\, (S3 + S4)\right) - b^2\, (S1 - S2)\, \left(d^2\, (S2 - S3)\, (S3 - S4) + c^2\, (S2 + S3)\, (S3 + S4)\right)\right) + a\, \left(c^2\, (S1 - S2)\, \left(d^2\, (S2 + S3)\, (S3 - S4) + c^2\, (S2 - S3)\, (S3 + S4)\right) + b^2\, (S1 + S2)\, \left(d^2\, (S2 - S3)\, (S3 - S4) + c^2\, (S2 + S3)\, (S3 + S4)\right)\right)\right) \mathrm{Cos}[\theta]\right) \Big/ \left(b^2\, (S0 - S1)\, \left(c^2\, (S1 + S2)\, \left(d^2\, (S2 + S3)\, (S3 - S4) + c^2\, (S2 - S3)\, (S3 + S4)\right) + b^2\, (S1 - S2)\, \left(d^2\, (S2 - S3)\, (S3 - S4) + c^2\, (S2 + S3)\, (S3 + S4)\right)\right) + a^2\, (S0 + S1)\, \left(c^2\, (S1 - S2)\, \left(d^2\, (S2 + S3)\, (S3 - S4) + c^2\, (S2 - S3)\, (S3 + S4)\right) + b^2\, (S1 + S2)\, \left(d^2\, (S2 - S3)\, (S3 - S4) + c^2\, (S2 + S3)\, (S3 + S4)\right)\right)\right)$$

/ * Step 3 : Validation : if myelin thickness is zero, there shall be no voltage drop across it. * /

```
a = b
```

b

```
Vmyelin
```

0
