# Supplementary material for: Shielding effects of myelin sheath on axolemma depolarization under transverse electric field stimulation
Source: PeerJ. 2018 Dec 3;6:e6020. doi: 10.7717/peerj.6020 (PMC6282940; doi:10.7717/peerj.6020)
Supplement: Supplemental Information 3 [file peerj-06-6020-s003.pdf]

/* Transmembrane potential acorss the axonal membrane,
in an unmyelinated axons that is exposed to a tranverse electric field * /

/ Step 1 : Vm for the myelinated axon can be further siplified for the unmyelinated axon,
if we assume S1 = S0, and S2 = S0. * /

$$\mathbf{Vm} = \left(8\, a^2 b^2 c\, (c - d)\, E0\, S0\, S1\, S2 \left(d \left(-S3 + S4\right) + c \left(S3 + S4\right)\right) \mathrm{Cos}[\theta]\right) \Big/ \left(b^2 \left(S0 - S1\right) \left(c^2 \left(S1 + S2\right) \left(d^2 \left(S2 + S3\right) \left(S3 - S4\right) + c^2 \left(S2 - S3\right) \left(S3 + S4\right)\right) + b^2 \left(S1 - S2\right) \left(d^2 \left(S2 - S3\right) \left(S3 - S4\right) + c^2 \left(S2 + S3\right) \left(S3 + S4\right)\right)\right) + a^2 \left(S0 + S1\right) \left(c^2 \left(S1 - S2\right) \left(d^2 \left(S2 + S3\right) \left(S3 - S4\right) + c^2 \left(S2 - S3\right) \left(S3 + S4\right)\right) + b^2 \left(S1 + S2\right) \left(d^2 \left(S2 - S3\right) \left(S3 - S4\right) + c^2 \left(S2 + S3\right) \left(S3 + S4\right)\right)\right)\right)$$

$$\left(8\, a^2 b^2 c\, (c - d)\, E0\, S0\, S1\, S2\, (d\, (-S3 + S4) + c\, (S3 + S4))\, \mathrm{Cos}[\theta]\right) \Big/ \left(b^2\, (S0 - S1) \left(c^2\, (S1 + S2) \left(d^2\, (S2 + S3)\, (S3 - S4) + c^2\, (S2 - S3)\, (S3 + S4)\right) + b^2\, (S1 - S2) \left(d^2\, (S2 - S3)\, (S3 - S4) + c^2\, (S2 + S3)\, (S3 + S4)\right)\right) + a^2\, (S0 + S1) \left(c^2\, (S1 - S2) \left(d^2\, (S2 + S3)\, (S3 - S4) + c^2\, (S2 - S3)\, (S3 + S4)\right) + b^2\, (S1 + S2) \left(d^2\, (S2 - S3)\, (S3 - S4) + c^2\, (S2 + S3)\, (S3 + S4)\right)\right)\right)$$

S1 = S0

S0

S2 = S0

S0

Vm

$$\frac{2\, c\, (c - d)\, E0\, S0\, (d\, (-S3 + S4) + c\, (S3 + S4))\, \mathrm{Cos}[\theta]}{d^2\, (S0 - S3)\, (S3 - S4) + c^2\, (S0 + S3)\, (S3 + S4)}$$

/* Step 2 : Validation,
Transmembrane potential shall be zero if the membrane has zero thickness * /

c = d

d

Vm

0
